# ﻿The Bolboceratidae, Geotrupidae (Coleoptera, Scarabaeoidea), and Scarabaeinae (Coleoptera, Scarabaeoidea, Scarabaeidae) fauna of Honduras: Taxonomy and distribution

**DOI:** 10.3897/zookeys.1255.150586

**Published:** 2025-10-10

**Authors:** José D. Rivera-Duarte, Renato P. Salomão, Ángel Solís, Mauricio Michel, Marcela I. Matamoros, Bert Kohlmann

**Affiliations:** 1 Laboratorio de Hidrobiología, Departamento de Ecología y Recursos Naturales, Escuela de Biología, Facultad de Ciencias, Universidad Nacional Autónoma de Honduras, Francisco Morazán, Honduras Universidad Nacional Autónoma de Honduras Francisco Morazán Honduras; 2 Facultad de Estudios Superiores Iztacala, Universidad Nacional Autónoma de México, Tlalnepantla de Baz, Mexico Universidad Nacional Autónoma de México Tlalnepantla de Baz Mexico; 3 BioAlfa Barcoding Project, Santo Domingo de Heredia, Costa Rica BioAlfa Barcoding Project Santo Domingo de Heredia Costa Rica; 4 Escuela Agrícola Panamericana, Departamento de Ciencia Agrícola y Producción, Colección de Insectos, Zamorano, Honduras Departamento de Ciencia Agrícola y Producción, Colección de Insectos Zamorano Honduras

**Keywords:** Central America, distribution atlas, endemics, identification key, Neotropics, new species, species revalidation, synonymization

## Abstract

A biodiversity and distribution analysis of the beetle families Bolboceratidae and Geotrupidae, and the subfamily Scarabaeinae (Scarabaeidae) of Honduras is presented for the first time. The checklist contains 98 species: two Bolboceratidae, one Geotrupidae, and 95 Scarabaeinae. The fauna of Honduras includes two genera of Bolboceratidae, one genus of Geotrupidae, and 22 of Scarabaeinae. *Onthophagus* is the most speciose genus, with 21 species, followed by *Canthon* (10 species) and *Copris* (9 species). Valid names and synonyms are provided. First country records, notes on presently recognized species, distribution maps, and biodiversity comparisons are included, and nomenclatural problems associated with species in question are discussed. *Phanaeus lempira* Kohlmann, Solís and Rivera, **sp. nov.** (Coleoptera: Scarabaeidae) is described as a new species. *Canthon sallei* Harold, 1863 and *Canthon spinosus* Harold, 1863 are reinstated as valid species. *Canthon cyanellus* LeConte, 1859 does not contain any subspecies. Two new synonymies are established: *Canthon cyanellus violetae* Halffter, 1961, **syn. nov.** of *Canthon sallei* Harold, 1863; *Canthon speciosus* Harold, 1868, **syn. nov.** of *Canthon spinosus* Harold, 1863.

## ﻿Introduction

The dung beetles of the subfamily Scarabaeinae comprise 6,939 described species and belong to the Scarabaeidae family, which contains a total of 36,455 species (Schoolmeesters 2025). Dung beetles are among the most studied and well-known insect groups (Hanski and Cambefort 1991; Nichols et al. 2007; Scholtz et al. 2009; Cupello et al. 2023). Although they are distributed worldwide, they are more abundant in the tropics than in other geographical regions (Gill 1991). Most Scarabaeinae use dung as their main food resource; there are other common diets, such as necrophagy, saprophagy, and mycetophagy (Simmons and Ridsdill-Smith 2011). As with their diet, Scarabaeinae nesting behavior has been well studied, and three main strategies have been described: rollers, tunnellers, and dweller species. Perhaps a fourth category should be established, the pushers, the ones that push dung with their heads and forward parts of their body. Nevertheless, these food relocation behaviors have evolved multiple times in multiple phyletic lineages (Halffter and Edmonds 1982; Emlen and Philips 2006; Huerta et al. 2023). Because of their feeding and nesting behavior, dung beetles play vital ecological roles, including nutrient recycling, soil turnover, secondary seed dispersal, and livestock parasite control (Nichols et al. 2008; Andresen and Urrea-Galeano 2022). Although the largest and dominant dung beetle genera are all still incompletely or fully unrevised (Cupello et al. 2023), their quick responsiveness to habitat alterations make dung beetles highly effective biological indicators (Favila and Halffter 1997; Kohlmann et al. 2007), an application that has been rising since the 1990s (Rivera and Favila 2022).

Bolboceratidae and Geotrupidae are part of the Scarabaeoidea superfamily and comprise approximately 641 and 499 species, respectively (see Zunino 1984; Howden 1985a, b; Scholtz and Browne 1996; Browne and Scholtz 2002; Schoolmeesters 2025). Although Geotrupidae are predominantly coprophagous, some species feed on organic matter (e.g., acorns, fungi, grapevine leaves) and carrion (Pérez-Ramos et al. 2007; Pérez-Villamares et al. 2016). Geotrupidae exhibits its highest diversity in subtropical and temperate ecosystems, but they can also be found in tropical regions (Trotta-Moreu et al. 2007; Arriaga-Jiménez et al. 2023). Furthermore, Geotrupidae species show various patterns of spatial distribution, ranging from eurytopic species to those specialized in conserved (e.g., pine-oak forests) or disturbed habitats (e.g., pasturelands, plantations) (Kohlmann 1991; Trotta-Moreu et al. 2007; Arriaga-Jiménez et al. 2023). Regarding Bolboceratidae, some species are mycetophagids, feeding on the fungus mycelium, while others may be aphagous (Nikolajev 1987). Adult beetles dig vertical holes and use the soil humus for larval feeding (Howden 1982). Bolboceratidae is a cosmopolitan family, with its highest taxonomic diversity in the southern hemisphere.

Despite the popularity of dung beetles and related groups as a subject of study in Latin America (see Favila et al. 2023), research in Honduras is considerably limited. To date, the country has only two scientific articles (Rivera and Cantarero 2011; Rivera and Schlein 2014), one identification manual (Creedy and Mann 2012), and four undergraduate theses (Jut Solórzano 2012; Cerritos García and Ochoa Cadena 2017; Alvarado López and Osorio Kattan 2020; Engle 2020) that are focused on dung beetle biological and ecological aspects. The knowledge of dung beetle diversity and distribution in Honduras is thus primarily confined to these studies, supplemented by a few taxonomic revisions of various genera that occasionally include species collected within Honduran territory (e.g., Solís and Kohlmann 2004; Edmonds and Zídek 2010, 2012; Darling and Génier 2018; Moctezuma and Halffter 2021a). To date, no specific research has been conducted on the Bolboceratidae and Geotrupidae families in Honduras, and similar to Scarabaeinae species, previous records for the country originate from taxonomic revisions that include some species collected from the region.

This work presents an updated checklist of the Bolboceratidae, Geotrupidae, and Scarabaeidae (Scarabaeinae) beetle fauna of Honduras. Additionally, we include the description of a new species of *Phanaeus* and a taxonomic review of *Canthon sallei*, *C. spinosus*, and *Sisyphus mexicanus*. This study aims to lay the groundwork for future research on dung beetles in Honduras, encouraging a greater understanding of their diversity and biogeography in the Americas.

## ﻿Materials and methods

The checklist of dung beetles of Honduras was compiled from three main sources: (1) fieldwork involving systematic collections using pitfall traps baited with human feces, conducted across various sites in the central, western, northeastern, and eastern regions of the country during 2023 and 2024 (see below, Study sites and Dung beetle sampling); (2) a review of the entomological collection at the Escuela Agrícola Panamericana El Zamorano; and (3) a thorough review of reliable bibliographic sources focused on records of specimens collected within Honduras and their sampling localities.

### ﻿Study sites

Honduras is located in the center of the Central American isthmus, between 13°33'16" and 15°01'08"N and between 83°08'89" and 89°21'49"W. It is bordered to the northwest by Guatemala, to the southwest by El Salvador, to the north by the Caribbean Sea, to the south by the Golfo de Fonseca of the Pacific Ocean, and to the east by Nicaragua. It is the second-largest country in Central America, with a territorial extension of 112,492 km^2^ and a forested area that represents 56% of the territory (6,315 million hectares), of which 36% is protected within the 91 declared protected areas. These include terrestrial, aquatic, and coastal marine ecosystems (Vreugdenhil et al. 2002).

Due to volcanic activity and orogenic processes in past times, it is the most mountainous territory in the region, with two-thirds of its territory covered by mountains (63%) and a smaller proportion of plains along its coasts (37%) (Mejía Ordóñez and House 2002). Such orographic conditions create regions with distinct and contrasting precipitation and temperature patterns. Mejía Ordóñez and House (2002) classify the territory into three large geomorphic regions: the Pacific Coastal Plain, the Northern Coastal Plain, and the Mountainous Region made up of the Northern, Central, and Southern ridges.

**Figure 1.**
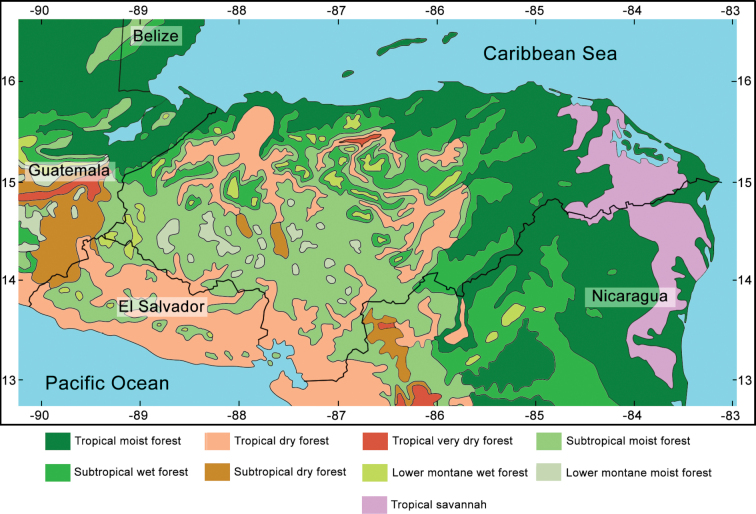
Natural life zones of Honduras.

Honduras has average annual temperatures in typical ranges of the tropical zone between 18 and 30 °C. The climate is characterized by a precipitation regime with two distinct seasons: the dry season, from November to May, and the rainy season, from June to October, resulting in an average annual precipitation of 800–3,200 mm (ICF 2022). Such climatic conditions determine the different life zones of the country (Fig. 1), which range from tropical pine savannas to low montane rainforests (cloud forest) and subtropical rainforests (pine forest) (ICF 2022). Sampling was performed in seven regions of Honduras representing contrasting ecosystems of the country: tropical dry forests, tropical cloud forests, tropical rainforests, and subtropical pine forests (Fig. 1).

*Celaque Mountain National Park* (PNMC, 14°34'N, 88°40'W) is located in the southwestern region of Honduras and covers an area of 26,393 hectares. The park spans parts of the departments of Lempira (19,630.53 ha), Ocotepeque (5,943.75 ha), and Copán (804.14 ha). The altitude in the region varies from 1,400 to 2,849 m a.s.l., with the latter representing the highest point in the country. The area receives annual rainfall between 1,600 mm and 1,800 mm, with relative humidity ranging from 74% to 78%. Furthermore, temperatures fluctuate between a maximum of 35 °C and a minimum of 18 °C. The dry season extends from November to April, with January and February being the driest months (ICF and MAPANCE 2016). According to the Holdridge classification, two life zones are identified within the PNMC: the subtropical humid forest, which covers 40% of the area in the buffer zones and features species such as *Liquidambar styraciflua* L. sweetgum, *Clethra* spp., and *Nectandra* spp.; and the lower montane humid forest, which covers ~60% of the PNMC’s area, including the park’s highest elevations, where species such as *Pinus maximinoi* H.E. Moore and *Pinus oocarpa* Schiede ex Schltdl are found (ICF and MAPANCE 2016).

*La Tigra National Park* (PNLT; 14°09'N, 87°08'W) is located in the central region of Honduras, in the department of Francisco Morazán, northeast of the capital, Tegucigalpa, M.D.C. It covers a total area of 24,040 hectares, spanning the municipalities of the Central District, Valle de Ángeles, Santa Lucía, and Cantarranas (ICF 2013a). La Tigra is part of the San Juancito mountain range, with elevations ranging from 1,200 to 2,290 m a.s.l. The annual average temperature varies between 13.6 °C and 22.8 °C. Two primary life zones are identified in the area. The first is the lower montane wet subtropical forest, which acts as a transitional zone between dry forest and pine forest. This zone is located at elevations ranging from 1,300 to 1,800 m a.s.l. and receives annual precipitation of 1,500–2,000 mm. The vegetation is predominantly dominated by *Pinus maximinoi*, often in association with *L. styraciflua*. The second life zone is the lower montane, which comprises cloud forests found above 1,800 m a.s.l. This zone experiences an average annual precipitation of ~ 2,000 mm and is characterized by broadleaf forest cover, including species such as giant oak (*Quercus* sp.) and various species of “aguacatillo” (*Nectandra* sp., *Ocotea* sp., *Persea* sp.) (ICF 2013a).

Cerro Azul Meámbar National Park (PANACAM; 14°50'N, 87°47'W) is located in the central region of Honduras, east of Lake Yojoa. The park encompasses an area of 31,339 hectares, covering the municipalities of Meámbar, Siguatepeque, and Taulabé in the department of Comayagua, as well as the municipality of Santa Cruz de Yojoa in the department of Cortés. The altitude within the park ranges from 400 to 2,090 m a.s.l., with mean annual precipitation ranging from 1,600–2,000 mm and average temperatures between 8 °C and 25 °C, depending on the elevation (ICF 2012). The area is predominantly composed of montane and submontane evergreen forests (Mejía Ordóñez and House 2002), featuring tree species such as *P. oocarpa*, *Arbutus xalapensis* Kunth, *Magnolia hondurensis* A.M.Molina, and *Acacia farnesiana* (L.).

*Habitat/Species Management Refuge of the Honduran Colibrí Esmeralda* (Olanchito, 15°25'N, 86°52'W) is a reserve located in Olanchito municipality, Yoro department. This reserve consists primarily of lowland tropical dry forests, with an average elevation of 400 m a.s.l., mean temperatures ranging from 20°C to 33°C, and an average annual rainfall of 500 mm (ASESORA 2009). The vegetation is composed mainly of cacti (e.g., *Opuntia hondurensis* Standl., *Stenocereus yunckeri* (Standl.) M. Bravo and Sánchez-Mej), shrubs (e.g., *Aphelandra deppeana* Schltdl. & Cham., *Lonchocarpus trifolius* Standl. & L.O. Williams.), and trees (e.g., *Guaiacum sanctum* L., *Leucaena lempirana* C.E.Hughes), forming a mosaic of open and closed canopy vegetation (House 2004). The region is highly seasonal, with a rainy period (mean monthly rainfall: 545 mm) from May to October and a dry period (mean monthly rainfall: 187 mm) from November to April (ASESORA 2009). Although the reserve is conserved, within its surroundings, there are patches of anthropogenic activities dedicated to livestock practices and Honduran Army field training.

National Park Sierra de Agalta (PNSA, 14°54'N, 85°52'W, with elevations ranging from 600 to 1,000 m a.s.l.) and its surrounding agricultural regions (14°46'N, 85°46'W, ca 320 m a.s.l.) encompass the municipalities of Catacamas, Culmí, Santa María del Real, and Gualaco, located in the Olancho department, east of Honduras. The mean temperature ranges from 25.2°C to 28°C, with an annual rainfall of 1,368 mm (ICF 2002). The vegetation in the park includes tropical moist forests in the lowland areas, gradually transitioning to lower montane wet forests (cloud forests) at higher elevations (Portillo et al. 2013). The rainy season extends from May to October, with a mean monthly rainfall of 383 mm, while the dry season occupies the remainder of the year, with a mean monthly rainfall of 124 mm (data obtained from Catacamas Municipality in Weather and Climate 2024). Although the portion of the National Park is moderately conserved, featuring small human settlements (i.e., villages) within its boundaries, the surrounding agricultural region consists of small forest remnants interspersed within a livestock-dominated landscape.

Brus Laguna is a municipality located in northeastern Honduras, within the Department of Gracias a Dios (15°44'N, 84°29'W). It borders the Departments of Olancho to the west and Colón to the north. Brus Laguna, with an area of 321,234.82 hectares, constitutes 38.59% of the Reserva del Hombre y Río Plátano Biosphere Reserve (RHRP-BR), one of the most important protected areas in the Mesoamerican Biological Corridor and one of the largest in Honduras, covering 832,338.963 hectares (ICF 2013b). The region’s altitude varies from 0 to 710 m a.s.l., with an average temperature of 25 °C (minimums of 23 °C and maximums of 33 °C). Annual precipitation ranges from 2,000–4,000 mm, with the rainy season spanning from May to November (ICF 2013b). The main ecosystems of the surveyed areas include tropical moist forests and tropical flooded forests of pine savannas. The former is composed of a layer of mature broadleaf trees up to 35 meters tall, with dominant species such as *Swietenia macrophylla* King, *Cedrela odorata* L., and *Cordia alliodora* (Ruiz & Pav.) Oken. On the other hand, the pine savanna is dominated by *Pinus caribaea* Morelet, which grows alongside grasses, palms, and some fire-resistant trees. The pines can reach a height of 15–20 m, and the canopy is covered by a dense layer of herbaceous vegetation and grasses, with swamp vegetation (ICF 2013b).

### ﻿Dung beetle sampling

Dung beetles were surveyed during June and July 2023, which comprised the rainy season. The surveyed regions were at least 130 km from each other. In each region, sampling was performed in four sites at least 2 km apart, except for Brus Laguna, where only three sites were sampled, totaling *n* = 23 study sites. To obtain a trustworthy sample of dung beetles from each study site, ten pitfall traps baited with ~ 25 g of fresh human feces were installed in a linear transect, with a distance of ca 40 m from the nearest ones (methodology adapted from Rivera and Favila 2022; Mora-Aguilar et al. 2023). Pitfall traps consisted of a 1,000-ml plastic cup that was filled with ~ 250 ml of a mixture of water, neutral detergent (~ 5 ml), and salt (~ 10 g); such mixture served to capture and conserve the dung beetles that fell in the traps. Above the plastic cup, a 50-ml recipient was attached with the attractive bait; a plastic lid was attached to the pitfall trap to avoid the entrance of rainwater and litter. After two days, the pitfall traps were checked, collecting and storing dung beetles in vials with 70% ethanol.

Complementary samplings were conducted at the Universidad Nacional Autónoma de Honduras (UNAH) between March and July 2024, covering the end of the dry season and the beginning of the rainy season. Nine dry forest patches were selected, in which four or five pitfall traps were installed per patch. The traps were arranged in linear transects, spaced 40 meters apart, and baited with 25 g of fresh human feces. Finally, a single sampling event was conducted in September 2024 at Los Olingos Lodge, located within a lowland tropical moist forest in the community of Agua Chiquita in the Department of Atlántida (15°47'1.74"N, 87°38'19.17"W), using ten pitfall traps baited with human feces.

Species identifications of the collected specimens were performed with the aid of taxonomic keys (e.g., Solís and Kohlmann 2002, 2004, 2013; Edmonds and Zídek 2012; Darling and Génier 2018; Cupello et al. 2023; Hernández et al. 2024a, b) and information by two of the coauthors of this study (BK and ÁS).

### ﻿Photography

Several habitus’ photos were taken by the co-author MM using a Canon EOS Rebel T5i camera mounted on a LEICA EZ4 stereoscope. Another set of photos was taken by ÁS using an Olympus E-M10 Mark III digital camera mounted on a WeMacro focus stacking rail, and then processed with Helicon Focus 8.2.7 Lite stacking software. Finally, BK took some detailed photos using an Adonstar 249S-M Digital Microscope system.

## ﻿Results

**Figure 2.**
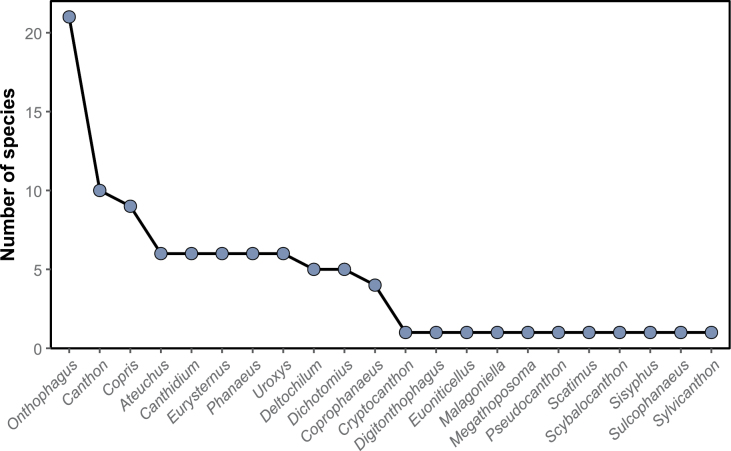
Rank-abundance curve with the dung beetle genera distribution according to the number of species recorded for each genus.

We recorded two species of Bolboceratidae, one species of Geotrupidae, and 95 of Scarabaeinae in Honduras. Among these, four Scarabaeine species are endemic to the country, while two species have been introduced from the Old World (*Digitonthophagus gazella* and *Euoniticellus intermedius*). All these taxa belong to 25 genera and ten tribes. *Onthophagus* is the most species-rich genus, with 21 species, followed by *Canthon* and *Copris*, with ten and nine taxa, respectively (Fig. 2). Thirty-one species are registered for the first time in Honduras. Four species are registered as new and await description.

### ﻿Checklist, taxonomic notes, and remarks

In the present list, the following marks are used as appropriate: * = endemic species to the country, ** = introduced species, NR = new record, CS = Species collected in the field samplings, RL = species reported in the reviewed literature for Honduras, EZ = species with Honduran specimens housed in the collection of Escuela Agrícola Panamericana Zamorano. In the checklist, the classification of Bolboceratidae follows Scholtz and Browne (1996); the Geotrupinae tribal and generic classification follows Zunino (1984); the Scarabaeinae tribal classification follows Cupello et al. (2023). The checklist includes revised material from specimens collected during the 2023–2024 surveys, with taxonomic notes and additional remarks on selected species. The newly collected material is indicated below by species; this material is being housed in the Univeridad Nacional Autónoma de Honduras (**UNAH**) in Tegucigalpa.

#### ﻿BOLBOCERATIDAE

**Athyreini**

##### *Neoathyreus excavatus* (Castelnau, 1840) (RL)

*Neoathyreus quadridentatus* Howden, 1964.

Mexico, Guatemala, Honduras, El Salvador, Costa Rica, Panama, Colombia, Venezuela, Guyana, Suriname, French Guiana, Peru, Brazil, Trinidad and Tobago.

Castelnau 1840; Howden and Martínez 1978; Howden and Gill 1984; Howden 1985; Horgan 2002; Hielkema 2017; Pablo-Cea et al. 2023; Morales Morales et al. 2024.

#### ﻿Bolboceratini

##### Eucanthus (Bolbelasmus) arcuatus (Bates, 1887) (RL)

*Bolboceras arcuatus* Bates, 1887.

Mexico, Guatemala, Honduras, El Salvador, Nicaragua, Costa Rica, Panama.

Bates 1887; Howden 1964; Horgan 2008; Pablo-Cea et al. 2023; Morales Morales et al. 2024.

#### ﻿GEOTRUPIDAE

**Ceratotrupini**

##### *Haplogeotrupes guatemalensis tridentatus* Howden, 1974 (NR, CS, EZ)

Fig. 3A

**Figure 3.**
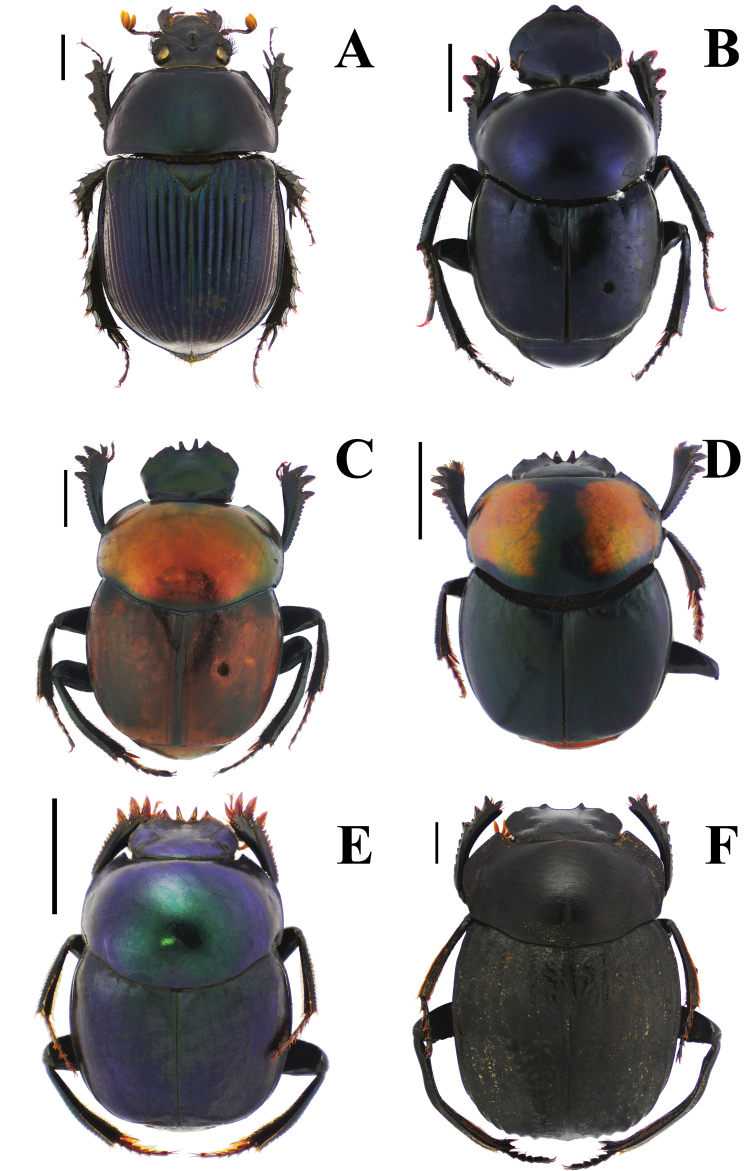
Habitus. A. *Haplogeotrupes guatemalensis tridentatus*, Parque Nacional Montaña de Celaque, Departamento de Lempira; B. *Canthon chiapas*, Refugio Hábitat/Especie Colibrí Esmeralda Hondureño (Olanchito), Departamento de Yoro; C. *Canthon sallei*, Refugio Hábitat/Especie Colibrí Esmeralda Hondureño (Olanchito), Departamento de Yoro; D. *Canthon sallei*, Brus Lagunas, Rio Platano Biosphere Reserve; E. *Canthon spinosus*, Agua Chiquita, Departamento de Atlántida; F. *Deltochilum scabriusculum*, Parque Nacional Sierra de Agalta, Departamento de Olancho. Scale bar: 2 mm.

*Geotrupes guatemalensis tridentatus* Howden, 1974.

Honduras, El Salvador.

Howden 1974; Pablo-Cea et al. 2023.

**Material examined. Honduras (22): Lempira**: Gracias, Parque Nacional Montaña de Celaque; 14°33'53"N, 88°37'54"W, 1622 m; 16.VI.2023; JD Rivera, R Salomão and M Michel leg.; pitfall trap, human dung; UNAH (4). Same locality; 14°29'5"N, 88°40'11"W, 2030 m; 17.VI.2023; JD Rivera, R Salomão and M Michel leg.; pitfall trap, human dung; UNAH (18).

**Remarks.** This subspecies was initially described based on four specimens from a cloud forest in Monte Cristo, El Salvador, collected in 1971 at 2300 m on horse dung (Howden 1974). Fifty-three years later, this taxon has been collected for a second time and it is a first for Honduras. The Honduran specimens were collected in both pine–oak and cloud forests. This subspecies is part of a polytypic species formed by *H. g. guatemalensis* (Bates, 1887) and *H. g. unidentatus* (Howden, 1974). *Haplogeotrupes* includes a second species that is troglophile, *reddelli* (Howden, 1967), from the Xóchitl cave in Puebla, Mexico, living inside the dung galleries of rodent nests. Zunino (1984), in his genus-level systematics of the Geotrupinae, considers *Haplogeotrupes* and its sister genus *Halffterius* to be the first branch to separate from the American geotrupid tree (Ceratotrupini). This branching cannot be regarded as recent and could have started already during the Paleogene (Zunino 1984).

#### ﻿SCARABAEIDAE

**Scarabaeinae**

**Ateuchini**

##### *Ateuchus candezei* (Harold, 1868) (RL)

*Choeridium poropyge* Bates, 1887.

Mexico, Guatemala, Belize, Honduras, Nicaragua, Costa Rica, Panama, Colombia.

Harold 1868a; Kohlmann 2000, 2003; Solís and Kohlmann 2012; Cupello 2022; Hernández et al. 2024a.

##### *Ateuchus colossus* Moctezuma, Sánchez-Huerta & Halffter, 2018 (RL)

Mexico, Honduras, El Salvador.

Moctezuma et al. 2018; Cupello 2022; Pablo-Cea et al. 2023.

##### *Ateuchus guatemalensis* (Bates, 1887) (RL, EZ)

*Ateuchus benitojuarezi* Moctezuma, Sánchez-Huerta & Halffter, 2018.

Mexico, Guatemala, Honduras, Nicaragua.

Bates 1887; Kohlmann 2000; Creedy and Mann 2012; Cupello 2022; Hernández et al. 2024a.

##### *Ateuchus perpusillus* Kohlmann, 2000* (RL)

Guatemala, Honduras.

Kohlmann 2000; Cupello 2022.

##### *Ateuchus rodriguezi* (Preudhomme de Borre, 1886) (CS, RL, EZ)

*Choeridium ampliatum* Bates, 1887.

Mexico, Guatemala, Honduras, El Salvador, Nicaragua, Costa Rica, Panama.

Preudhomme de Borre 1886; Bates 1887; Kohlmann 2000, 2003; Rivera and Cantarero 2011; Creedy and Mann 2012; Solís and Kohlmann 2012; Cupello 2022; Pablo-Cea et al. 2023; Hernández et al. 2024a.

**Material examined. Honduras (17): Olancho**: Catacamas, Parque Nacional Sierra de Agalta; 14°54'6"N, 85°52'25"W, 483 m; 3.VII.2023; JD Rivera and R Salomão leg.; pitfall trap, human dung; UNAH (1). **Yoro**: Olanchito, Refugio Hábitat/Especie Colibrí Esmeralda Hondureño; 15°27'53"N, 86°52'52"W, 412 m; 29.VI.2023; JD Rivera and R Salomão leg.; pitfall trap, human dung; UNAH (8). Same locality; 15°27'9"N, 86°53'41"W, 338 m; 29.VI.2023; JD Rivera and R Salomão leg.; pitfall trap, human dung; UNAH (4). Same locality; 15°25'41"N, 86°53'38"W, 242 m; 30.VI.2023; JD Rivera and R Salomão leg.; pitfall trap, human dung; UNAH (4).

##### *Ateuchus* sp.* (RL)

Honduras.

Cupello 2022.

##### *Scatimus ovatus* Harold, 1862 (NR, EZ)

*Scatimus patruelis* Preudhomme de Borre, 1866; *Scatimus quadridentatus* Balthasar, 1939b.

Mexico, Belize, El Salvador, Honduras, Nicaragua, Costa Rica, Panama, Colombia.

Harold 1862; Bates 1887; Balthasar 1939b; Génier and Kohlmann 2003; Kohlmann 2003; Hernández et al. 2020; Pablo-Cea et al. 2023.

#### ﻿Coprini

##### *Copris aspericollis* Gillet, 1910 (NR, EZ)

Guatemala, El Salvador, Honduras.

Gillet 1910; Matthews 1959, 1962; Pablo-Cea et al. 2023.

##### *Copris brevicornis* Darling & Génier, 2018 (RL)

Honduras, Nicaragua, Costa Rica, Panama.

Darling and Génier 2018; Maes et al. 2020.

##### *Copris costaricensis dolichocerus* Matthews, 1961 (CS, RL, EZ)

Mexico, Guatemala, Honduras.

Matthews 1962; Rivera and Cantarero 2011.

**Material examined. Honduras (40): Francisco Morazán**: Distrito Central, Universidad Nacional Autónoma de Honduras – Ciudad Universitaria; 14°5'2"N, 87°11'32"W, 990 m; 20.VII.2024; JD Rivera leg.; pitfall trap, human dung; UNAH (40).

##### *Copris laeviceps* Harold, 1869 (CS, RL, EZ)

Mexico, Guatemala, Belize, Honduras, El Salvador, Nicaragua, Costa Rica.

von Harold 1869; Bates 1887; Matthews 1962; Kohlmann 2003; Creedy and Mann 2012; Solís and Kohlmann 2012; Darling and Génier 2018; Maes et al. 2020; Pablo-Cea et al. 2023.

**Material examined. Honduras (9): Gracias a Dios**: Brus Lagunas, Reserva de Biosfera y el Hombre Río Plátano; 15°41'40"N, 84°33'43"W, 9 m; 8.VII.2023; JD Rivera and R Salomão leg.; pitfall trap, human dung; UNAH (1). Same locality; 15°42'45"N, 84°33'35"W, 13 m; 8.VII.2023; JD Rivera and R Salomão leg.; pitfall trap, human dung; UNAH (2). **Olancho**: Catacamas, Parque Nacional Sierra de Agalta; 14°55'1"N, 85°52'57"W, 586 m; 3.VII.2023; JD Rivera and R Salomão leg.; pitfall trap, human dung; UNAH (5). Catacamas, Aldea La Concepción; 14°45'57"N, 85°48'6"W, 320 m; 4.VII.2023; JD Rivera and R Salomão leg.; pitfall trap, human dung; UNAH (1).

##### *Copris lugubris* Boheman, 1858 (CS, RL, EZ)

Mexico, Guatemala, Belize, Honduras, El Salvador, Nicaragua, Costa Rica, Panama.

Boheman 1858; Bates 1887; Matthews 1962; Kohlmann 2003; Rivera and Cantarero 2011; Creedy and Mann 2012; Solís and Kohlmann 2012; Darling and Génier 2018; Maes et al. 2020; Pablo-Cea et al. 2023.

**Material examined. Honduras (11): Francisco Morazán**: Distrito Central, Universidad Nacional Autónoma de Honduras – Ciudad Universitaria; 14°5'2"N, 87°11'32"W, 990 m; 20.VII.2024; JD Rivera leg.; pitfall trap, human dung; UNAH (8). **Olancho**: Catacamas, Aldea La Concepción; 14°47'2"N, 85°45'32"W, 321 m; 4.VII.2023; JD Rivera and R Salomão leg.; pitfall trap, human dung; UNAH (1). **Yoro**: Olanchito, Refugio Hábitat/Especie Colibrí Esmeralda Hondureño; 15°27'53"N, 86°52'52"W, 412 m; 29.VI.2023; JD Rivera and R Salomão leg.; pitfall trap, human dung; UNAH (1). Same locality; 15°26'47"N, 86°53'14"W, 327 m; 29.VI.2023; JD Rivera and R Salomão leg.; pitfall trap, human dung; UNAH (1).

##### *Copris maesi* Ratcliffe, 1998 (RL)

Nicaragua, Honduras.

Ratcliffe 1998; Maes et al. 2020.

##### *Copris matthewsi pacificus* Delgado & Kohlmann, 2001 (NR, EZ)

Mexico, El Salvador, Honduras.

Delgado and Kohlmann 2001; Kohlmann 2003; Kohlmann et al. 2003; Pablo-Cea et al. 2023.

##### *Copris nubilosus* Kohlmann, Cano & Delgado, 2003* (RL)

Guatemala, Honduras.

Kohlmann et al. 2003; Creedy and Mann 2012.

##### *Copris* sp.* (RL)

Honduras.

Creedy and Mann 2012.

#### ﻿Deltochilini

##### *Canthon angustatus* Harold, 1867 (NR, EZ)

*Geocanthon angustatus* (Harold): Pereira and Martínez 1956.

Mexico, Guatemala, Belize, Honduras, Nicaragua, Costa Rica, Panama, Colombia, Ecuador, Peru.

Harold 1867b; Bates 1887; Howden and Young 1981; Medina et al. 2001; Solís and Kohlmann 2002, 2012; Maes et al. 2020.

##### *Canthon championi* Bates, 1887 (NR, CS)

Mexico, Guatemala, Honduras, El Salvador, Nicaragua.

Bates 1887; Rivera-Cervantes and Halffter 1999; Maes et al. 2020.

**Material examined. Honduras (210): Comayagua**: Siguatepeque, Parque Nacional Cerro Azul Meámbar; 14°50'40"N, 87°57'12"W, 748 m; 15.VII.2023; JD Rivera leg.; pitfall trap, human dung; UNAH (4). Same locality; 14°52'50"N, 87°54'59"W, 712 m; 16.VII.2023; JD Rivera leg.; pitfall trap, human dung; UNAH (50). **Olancho**: Catacamas, Parque Nacional Sierra de Agalta; 14°54'6"N, 85°52'25"W, 483 m; 3.VII.2023; JD Rivera and R Salomão leg.; pitfall trap, human dung; UNAH (3). Same locality; 14°55'1"N, 85°52'57"W, 586 m; 3.VII.2023; JD Rivera and R Salomão leg.; pitfall trap, human dung; UNAH (1). Catacamas, Aldea La Concepción; 14°45'57"N, 85°48'6"W, 320 m; 4.VII.2023; JD Rivera and R Salomão leg.; pitfall trap, human dung; UNAH (1). **Yoro**: Olanchito, Refugio Hábitat/Especie Colibrí Esmeralda Hondureño; 15°27'53"N, 86°52'52"W, 412 m; 29.VI.2023; JD Rivera and R Salomão leg.; pitfall trap, human dung; UNAH (80). Same locality; 15°27'9"N, 86°53'41"W, 338 m; 29.VI.2023; JD Rivera and R Salomão leg.; pitfall trap, human dung; UNAH (46). Same locality; 15°25'41"N, 86°53'38"W, 243 m; 30.VI.2023; JD Rivera and R Salomão leg.; pitfall trap, human dung; UNAH (25).

##### *Canthon chiapas* Robinson, 1948 (CS, EZ)

Fig. 3B

Mexico, Honduras, Nicaragua, Costa Rica, Panama.

Robinson 1948; Halffter 1961, 2003; Rivera-Gasperín et al. 2025; Moctezuma et al. 2025.

**Material examined. Honduras (659): Yoro**: Olanchito, Refugio Hábitat/Especie Colibrí Esmeralda Hondureño; 15°27'9"N, 86°53'41"W, 338 m; 29.VI.2023; JD Rivera and R Salomão leg.; pitfall trap, human dung; UNAH (74). Same locality; 15°25'41"N, 86°53'38"W, 243 m; 30.VI.2023; JD Rivera and R Salomão leg.; pitfall trap, human dung; UNAH (355). Same locality; 15°25'56"N, 86°52'31"W, 255 m; 30.VI.2023; JD Rivera and R Salomão leg.; pitfall trap, human dung; UNAH (230).

**Remarks.**Moctezuma et al. (2025) have recently published descriptions of two new species of the *Canthon indigaceus* species group from Honduras and Costa Rica, *Canthon hondurensis* and *Canthon woodruffi*, respectively. *Canthon chiapas*, also a member of the aforementioned species group, is under analysis by ÁS and BK for Central America. Their analyses suggest that the two species described by Moctezuma et al. (2025) are conspecific with *Canthon chiapas*. In the present paper we consider *C. chiapas* (sensu Rivera-Gasperín et al. 2025) as the valid species for Central America.

##### *Canthon deyrollei* Harold, 1868 (RL, EZ)

Honduras, El Salvador, Nicaragua, Costa Rica, Panama, Colombia.

Harold 1868b; Solís and Kohlmann 2002, 2012; Rivera and Cantarero 2011; Maes et al. 2020; Pablo-Cea et al. 2023.

##### *Canthon euryscelis* Bates, 1887 (CS, RL, EZ)

*Geocanthon euryscelis* (Bates): Halffter 1961.

Mexico, Guatemala, Belize, Honduras, El Salvador, Nicaragua, Costa Rica, Panama.

Bates 1887; Howden 1966; Howden and Young 1981; Rivera-Cervantes and Halffter 1999; Solís and Kohlmann 2002, 2012; Creedy and Mann 2012; Maes et al. 2020.

**Material examined. Honduras (144): Atlántida**: Tela, Los Olingos Lodge; 15°46'59"N, 87°38'9"W, 12 m; 18.VII.2024; JD Rivera leg.; pitfall trap, human dung; UNAH (50). **Gracias a Dios**: Brus Lagunas, Reserva de Biosfera y el Hombre Río Plátano; 15°41'40"N, 84°33'43"W, 9 m; 8.VII.2023; JD Rivera and R Salomão leg.; pitfall trap, human dung; UNAH (50). Same locality; 15°42'45"N, 84°33'35"W, 13 m; 8.VII.2023; JD Rivera and R Salomão leg.; pitfall trap, human dung; UNAH (44).

##### *Canthon meridionalis* (Martínez, Halffter & Halffter, 1964) (CS, RL, EZ)

*Glaphyrocanthon viridis meridionalis* Martínez, Halffter & Halffter, 1964.

Honduras, El Salvador, Nicaragua, Costa Rica, Panama.

Martínez et al. 1964; Rivera-Cervantes and Halffter 1999; Solís and Kohlmann 2002; 2012, Rivera and Cantarero 2011; Maes et al. 2020; Pablo-Cea et al. 2023.

**Material examined. Honduras (159): Comayagua**: Siguatepeque, Parque Nacional Cerro Azul Meámbar; 14°50'40"N, 87°57'12"W, 748 m; 15.VII.2023; JD Rivera leg.; pitfall trap, human dung; UNAH (1). Same locality; 14°52'50"N, 87°54'59"W, 712 m; 16.VII.2023; JD Rivera leg.; pitfall trap, human dung; UNAH (14). **Francisco Morazán**: Distrito Central, Universidad Nacional Autónoma de Honduras – Ciudad Universitaria; 14°5'2"N, 87°11'32"W, 990 m; 20.VII.2024; JD Rivera leg.; pitfall trap, human dung; UNAH (13). **Olancho**: Catacamas, Parque Nacional Sierra de Agalta; 14°54'6"N, 85°52'25"W, 483 m; 3.VII.2023; JD Rivera and R Salomão leg.; pitfall trap, human dung; UNAH (1). **Yoro**: Olanchito, Refugio Hábitat/Especie Colibrí Esmeralda Hondureño; 15°27'53"N, 86°52'52"W, 412 m; 29.VI.2023; JD Rivera and R Salomão leg.; pitfall trap, human dung; UNAH (65). Same locality; 15°27'9"N, 86°53'41"W, 338 m; 29.VI.2023; JD Rivera and R Salomão leg.; pitfall trap, human dung; UNAH (21). Same locality; 15°25'41"N, 86°53'38"W, 243 m; 30.VI.2023; JD Rivera and R Salomão leg.; pitfall trap, human dung; UNAH (20). Same locality; 15°25'57"N, 86°52'31"W, 255 m; 30.VI.2023; JD Rivera and R Salomão leg.; pitfall trap, human dung; UNAH (24).

##### *Canthon morsei* Howden, 1966 (NR, CS)

Mexico, El Salvador, Honduras, Costa Rica, Panama, Colombia, Ecuador.

Howden 1966; Howden and Young 1981; Solís and Kohlmann 2002, 2012; Halffter 2003; Pablo-Cea et al. 2023.

**Material examined. Honduras**: (1) **Olancho**: Catacamas, Parque Nacional Sierra de Agalta; 14°55'1"N, 85°52'57"W; 3.VII.2023; 586 m; JD Rivera and R Salomão leg.; pitfall trap, human dung; UNAH.

##### *Canthon sallei* Harold, 1863, reinstated (CS, RL, EZ)

Fig. 3C, D

*Canthon cyanellus sallei* Schmidt, 1922.

*Canthon cyanellus violetae* Halffter, 1961, syn. nov.

Mexico, Honduras, Nicaragua, Costa Rica, Panama, Colombia.

*Canthon sallei*Harold 1863: 174 (description); Harold 1868b: 39 (genus key); von Harold 1869: 993 (catalogue); Bates 1887: 26 (revision of Central American Scarabaeinae); Pittier and Biolley 1895: 13 (species list); Gillet 1911: 33 (catalogue); Schmidt 1920: 123 (redescription); Balthasar 1939b: 211 (genus key); Martínez 1988: 89 (species list).

*Canthon cyanellus sallei* — Schmidt 1922: 74 (new status); Halffter 1961: 259, 265 (monograph); Howden 1966: 727 (species list); Halffter and Martínez 1977: 90 (description and species list); Martínez 1988: 89 (species list); Medina et al. 2001: 136 (species list); Nolasco et al. 2017: 181, 2020: 2, 2023: 2 (phylogeography and morphology).

*Canthon cyanellum sallei* — Blackwelder 1944: 199 (catalogue); Gacharná 1951: 221 (catalogue).

*Canthon cyanellus violetae* Halffter, 1961: 259 (description); Howden 1966: 727 (species list); Halffter and Martínez 1977: 90 (species list and description); Nolasco et al. 2017: 181, 2020: 2, 2023: 2 (phylogeography and morphology).

**Type material.** Syntypes deposited at the Natural History Museum, London, were studied from photographs.

**Material examined (624): Honduras (21). Gracias a Dios**: Brus Lagunas, Reserva de Biosfera y el Hombre Río Plátano; Rivera and Salomão *leg.*; (1) 9 m; 8.VII.2023; (1); **Lempira.** Celaque, Parque Nacional de Montaña Celaque, Rivera and Salomão *leg.*; 2.8 (1) 1283 m; **Olancho**, Parque Nacional Sierra de Agalta, Rivera and Salomão *leg.*; 3.VII.2023, 1.4 (1) 479 m; Aldea La Concepción, Catacamas; 3.1 (1) 323 m, 3.3 (1) 337 m, 3.10 (1) 315 m: **Yoro**, Olanchito, Área de Manejo del Hábitat / Especie Colibrí Esmeralda Hondureño, Rivera and Salomão *leg.*; 29.VI.2023, 2.7 (1) 346 m, 3.1 (2) 252 m; 30.VI.2023, 3.1 (1) 252 m, 3.5 (3) 246 m, 3.7 (1) 243 m, 3.8 (1) 243 m, 4.3 (1) 251 m, 4.5 (1) 256 m, 4.7 (1) 258 m, 4.8 (3) 257 m. **NICARAGUA (9). Managua.** Reserva Privada El Bajo, 649–709 m, 27-28.XI.2019, B. Hernández *leg.*, Sendero Húmedo (6), Sendero Ensueño, (2); Dep. Chinandega. Estero Real, Cervantes, 35 m, 22.VI.2019. B. Hernández *leg.* (1). **COSTA RICA (580). Alajuela.** Caño Negro, en el Pueblo, 10.8938°N, 84.78884°W, 20 m, Martínez Kattia *leg.*, 10.X.1992, (1); Cebadilla, 9.95653°N, 84.34518°W, 540 m, Solís & Alvarado *leg.*, 25.VIII.1985, (1); Guatuso, 10.6395°N, 84.90911°W, 300 m, Rodríguez Gladys *leg.*, 23.II.1998, (2); Rio Barranca, 450 m, Asolis001582, A. Solís *leg.*, 19.VI.2020, (1); Rio San Lorencito, 5 km N de Colonia Palmarena, 10.2266°N, 84.63249°W, 900 m, I Scarabaeidae course *leg.*, 13.VI.1993, (1); **Guanacaste.** 3 km NO de Nacaome, P. N. Barra Honda, 10.1682°N, 85.37365°W, 100 m, Reyes Marielos *leg.*, 5.VII.1992, (6); 3.VIII.1992, (5); 14.IX.1992, (1); 3.V.1993, (5); 1.VII.1993, (1); 2.VII.1993, (1); 1.X.1993, (2); 1.XI.1993, (4); 1.VIII.1994, (1); 1.X.1994, (1); 1.XI.1994, (3); 1.IV.1995, (6); 1.VII.1995, (3); 1.IX.1996, (1); A.C.A, La Cruz La Garita Est Los Almendros, 11.0338°N, 85.52479°W, 280 m, López Elba *leg.*, 1.VII.1992, (8); 28.VII.1992, (3); 24.VIII.1992, (4); 23.IX.1992, (6); 23.X.1992, (3); 21.XI.1992, (3); 11.VI.1993, (4); 7.IX.1993, (1); Martínez Kattia *leg.*, 13.X.1993, (1); 300 m, López Elba *leg.*, 3.VIII.1993, (1); 3.X.1993, (3); 7.VII.1994, (1); 4.IX.1994, (2); 2.X.1994, (2); 4.XI.1994, (3); 16.VII.1995, (3); 22.VIII.1995, (13); 28.X.1995, (1); Bagaces, 10.3491°N, 85.35234°W, 10 m, Acevedo Daniel *leg.*, 1.VI.1991, (1); 1.VIII.1991, (1); Chavarría Ulises *leg.*, 1.VIII.1991, (1); 4.V.1992, (1); 10.3666°N, 85.38326°W, 25 m, Porras Wendy *leg.*, 25.VIII.1999, (2); 30.IX.1999, (3); 29.XI.1999, (2); Bagaces, Pque Nal Palo Verde, Línea Vieja, 10.3609°N, 85.33640°W, 10 m, Porras Wendy *leg.*, 30.X.1999, (1); 6.V.2000, (2); Cañas, 10.3477°N, 85.35036°W, 50 m, Solís Ángel *leg.*, 6.VII.1985, (1); Cerro El Hacha, 12 km SE de La Cruz, 10.9831°N, 85.54106°W, 300 m, Solís Ángel *leg.*, 23.VII.1986, (3); 27.VII.1986, (4); Cerro Loros., 9.92947°N, 85.38518°W, 500 m, Porras Wendy *leg.*, 8.XI.2002, (1); Est Los Almendros, 12 Km Carretera a Santa Cecilia, 11.0342°N, 85.52754°W, 280 m, López Elba *leg.*, 8.V.1994, (3); 15.VIII.1994, (6); 1.VI.1995, (2); 4.I.1996, (15); 14.V.1996, (5); 15.VII.1996, (3); 10.X.1996, (9); 9.XI.1996, (3); 23.XI.1996, (4); Estación Experimental Horizontes, 10.7054°N, 85.57930°W, 125 m, Moraga Calixto *leg.*, 27.VI.1993, (1); Estación Lomas Barbudal, 10.4950°N, 85.37794°W, 30 m, Acevedo Daniel *leg.*, 1.VII.1991, (2); Estación Murciélago, 8Km S.W. de Cuajiniquil, 10.9019°N, 85.73101°W, 100 m, Cano Carolina *leg.*, 15.V.1993, (2); Quesada Freddy *leg.*, 18.V.1993, (1); Estación Santa Rosa. Sendero Indio Desnudo., 10.8388°N, 85.62777°W, 300 m, Briceño Duvalier *leg.*, 24.X.2000, (1); Finca Jenny, 30 Km N de Liberia, 10.8740°N, 85.57997°W, 240 m, Araya Elda *leg.*, 10.VIII.1996, (2); 10.X.1996, (1); Finca Loaiciga, 6 Km S. de Santa Cecilia, 11.0125°N, 85.42771°W, 500 m, Moraga Calixto *leg.*, 6.I.1992, (1); Ríos Petrona *leg.*, 6.I.1992, (5); Finca YAFA, 11.0402°N, 85.50468°W, 320 m, Cano Carolina *leg.*, 19.I.1992, (1); La Cruz, 10.9831°N, 85.54106°W, 300 m, López Elba *leg.*, 1.IX.1991, (5); 1.X.1991, (3); 1.XI.1991, (3); 350 m, López Elba *leg.*, 1.XI.1991, (1); Solis & Alvarado *leg.*, 15.I.1988, (3); 10.9891°N, 85.42583°W, 700 m, Moraga Calixto *leg.*, 1.VI.1994, (8); 1.VIII.1994, (2); La Cruz, Estación Murciélago 8Km S.W. de Cuajiniquil, 10.9019°N, 85.73101°W, 70 m, Cano Carolina *leg.*, 3.X.1994, (5); Quesada Freddy *leg.*, 3.X.1994, (4); La Cruz, Pque Nal Guanacaste Lado SSW Cerro El Hacha, 10.9931°N, 85.54293°W, 350 m, Chacón Isidro *leg.*, 26.VII.1986, (7); La Cruz, Pque Nal Santa Rosa, 10.8563°N, 85.61191°W, 290 m, Janzen Daniel *leg.*, 1.XII.1982, (1); 1.VI.1984, (3); La Cruz, Santa Elena, P.N. Santa Rosa Estación Murciélago, 10.9019°N, 85.73101°W, 100 m, Cano Carolina *leg.*, 3.XII.1993, (1); Quesada Freddy *leg.*, 16.VI.1993, (2); 70 m, Cano Carolina *leg.*, 16.VI.1993, (4); 4.VIII.1994, (1); Quesada Freddy *leg.*, 6.VI.1994, (1); 4.VIII.1994, (1); 31.X.1994, (1); La Garita Est Los Almendros, 11.0338°N, 85.52479°W, 280 m, López Elba *leg.*, 7.I.1993, (1); Liberia, 10.8364°N, 85.61549°W, 300 m, Janzen Daniel *leg.*, 15.IX.1975, (1); 22.VI.1981, (1); 1.VII.1982, (1); 1.V.1985, (1); Moraga Roster *leg.*, 1.VII.1992, (5); Parataxónomos III curso *leg.*, 3.VI.1992, (8); 13.VI.1992, (2); Pereira Guillermo *leg.*, 1.VII.1992, (1); Liberia, P.N. Santa Rosa, 10.8364°N, 85.61550°W, 300 m, Zumbado Manuel *leg.*, 25.VI.1999, (1); Liberia, P.N.S.R. Playa Naranjo, 10.8027°N, 85.67479°W, 25.5 m, Alcázar Eliden *leg.*, 1.I.1991, (8); 1.II.1991, (5); Nicoya, Estación Barra Honda, 10.1698°N, 85.37913°W, 100 m, Porras Wendy *leg.*, 22.VI.2000, (1); 3.VIII.2000, (2); Nicoya, Estación Barra Honda””, 10.1698°N, 85.37913°W, 100 m, Porras Wendy *leg.*, 2.IX.2000, (1); Nicoya, San Antonio, Estación Corral de Piedra, 10.2385°N, 85.33080°W, 16 m, Gamboa Billen *leg.*, 3.VII.2005, (1); P. N. Barra Honda, 200 m, Asolis002536, A. Solís *leg.*, 18.V.2021, (1); P. N. Barra Honda., 10.1701°N, 85.34719°W, 100 m, Zumbado Manuel *leg.*, 30.V.1993, (1); P.N. Barra Honda, 10.1710°N, 85.34720°W, 150 m, Unknown *leg.*, 8.VII.2000, (3); P.N. Barra Honda. Los Mesones, 10.1700°N, 85.35084°W, 350 m, Reyes Marielos *leg.*, 1.VII.1995, (3); P.N. Guanacaste. Finca Jenny., 10.8663°N, 85.57720°W, 240 m, Araya Elda *leg.*, 20.V.1994, (1); P.N. Guanacaste. La Cruz. Estación Biológica Maritza., 10.9625°N, 85.49524°W, 600 m, Parataxónomos II curso *leg.*, 1.VIII.1990, (7); P. N. Santa Rosa, Bosque Húmedo, A. Solís *leg.*, 6.VI.2016, (1); P.N. Santa Rosa, Área Administrativa.””, 10.8399°N, 85.62648°W, 300 m, Janzen Daniel *leg.*, 1.VIII.1984, (1); Pococí, Río Sardinas, 10.6440°N, 83.74200°W, 10 m, Araya Flor *leg.*, 10.XI.1993, (1); Pococí, Río Sardinas Barra del Colorado, 10.6440°N, 83.74200°W, 10 m, Araya Flor *leg.*, 25.VIII.1992, (1); 1.IX.1992, (3); 18.VI.1993, (1); 16.VII.1993, (1); 15 m, Araya Flor *leg.*, 18.IV.1993, (2); 2.I.1994, (1); 16.III.1994, (1); Pococí, Río Sardinas Barra del Colorado””, 10.6440°N, 83.74200°W, 10 m, Araya Flor *leg.*, 11.XII.1992, (1); R.V.S. Bosque Diriá. Santa Cruz. Poza Redonda., 10.1737°N, 85.59588°W, 200 m, Barton Salazar Raúl *leg.*, 24.XI.1998, (1); Santa Cecilia, 11.0645°N, 85.41737°W, 325 m, Moraga Calixto *leg.*, 1.VI.1994, (5); Santa Cruz, Est B. N. Diriá, 10.1342°N, 85.63359°W, 970 m, Cárdenas Yow *leg.*, 15.IX.2002, (1); Sector Las Pailas, 4.5 Km SW del Volcán Rincón de la Vieja, 10.7767°N, 85.35191°W, 800 m, Rodriguez Gladys *leg.*, 15.VII.1992, (1); Tilarán, Tierras Morenas, 10.5710°N, 85.02594°W, 685 m, Rodriguez Gladys *leg.*, 1.XII.1994, (1); **Heredia.** Cinco Esquinas de Carrizal, 10.1092°N, 84.15359°W, 1800 m, Barrantes Gilbert *leg.*, 8.V.1988, (1); Sarapiquí, Est Biol La Selva 3 Km S de Puerto Viejo, 10.4311°N, 84.00333°W, 75 m, OET INBio ALAS project *leg.*, 1.XI.1992, (1); 1.VIII.1995, (2); Limón, A.C.L.A.C, San Miguel Albergue CASACODE Send Cerillos, 9.56835°N, 82.64647°W, 20 m, Lobo Manuel *leg.*, 23.II.1999, (1); 50 m, Zumbado Manuel *leg.*, 23.II.1999, (1); Amubri, Sendero Soki, 9.51482°N, 82.95537°W, 70 m, Gallardo Gerardina *leg.*, 2.V.1994, (2); 6.VI.1994, (3); 1.I.1995, (1); 4.IV.1995, (5); 9.VIII.1995, (1); 1.XI.1995, (1); 2.III.1996, (1); 1.IX.1996, (1); 1.VI.1997, (1); Mora Andrés *leg.*, 3.IV.1995, (2); Cerro Tortuguero, 10.5848°N, 83.52920°W, 50 m, Chavarría Ulises *leg.*, 1.VII.1990, (1); 60.5 m, Delgado Roberto *leg.*, 1.I.1992, (1); Est. Miramar, R. B. Hitoy Cerere, 9.63336°N, 83.00342°W, 500 m, Carballo Gerardo *leg.*, 1.VIII.1994, (3); **Limón.** Río Blanco, 9.99556°N, 83.10838°W, 34 m, Solís Manuel *leg.*, 11.IV.2011, (1); Limón, Valle de la Estrella, 9.67103°N, 83.02614°W, 160 m, Arana Wilfredo *leg.*, 22.IV.1999, (1); Pococí, Colorado Estación Cuatro Esquinas, 10.5395°N, 83.50648°W, 5 m, Delgado Roberto *leg.*, 1.XI.1990, (3); 23.IV.1991, (2); 1.IX.1991, (1); 1.I.1993, (1); Solano José *leg.*, 1.VI.1990, (2); Pococí, Estación Cuatro Esquinas, 10.5395°N, 83.50648°W, 5 m, Aguilar Reinaldo *leg.*, 1.IV.1989, (1); Pococí, Estación Cuatro Esquinas., 10.5395°N, 83.50648°W, 5 m, Delgado Roberto *leg.*, 1.IX.1992, (1); Pococí, Colorado, 10.5942°N, 83.71651°W, 150 m, Rojas Elías *leg.*, 26.VII.1992, (1); 1.VI.1993, (1); Pococí, Sector Cedrales de la Rita, 10.5273°N, 83.72578°W, 10 m, Rojas Elías *leg.*, 1.VIII.1995, (2); 1.XI.1995, (1); 1.I.1996, (1); 1.VIII.1996, (1); R.B. Hitoy Cerere, 9.67537°N, 83.02677°W, 120 m, Carballo Gerardo *leg.*, 1.VII.1993, (2); R.V.S. Gandoca Manzanillo. Centro Operativo Manzanillo., 9.63258°N, 82.65905°W, 50.5 m, Quesada Freddy *leg.*, 6.I.1993, (1); Taylor Karla *leg.*, 22.X.1992, (1); Talamanca, Amubri, 9.51934°N, 82.95627°W, 70 m, Gallardo Gerardina *leg.*, 12.X.1992, (1); 2.IX.1993, (1); 1.II.1994, (2); 24.VII.1994, (1); 1.VIII.1994, (1); 1.X.1994, (1); Talamanca, Amubri, 9.51934°N, 82.95627°W, 70 m, Gallardo Gerardina *leg.*, 16.VIII.1992, (1); 12.XI.1992, (1); Talamanca, Bratsi, 9.51934°N, 82.95627°W, 70 m, Gallardo Gerardina *leg.*, 5.I.1993, (1); Talamanca, Valle La Estrella, 9.67176°N, 83.02770°W, 100 m, Carballo Gerardo *leg.*, 1.VI.1994, (1); Guzmán Rosa *leg.*, 19.IV.1992, (1); López Elba *leg.*, 19.IV.1992, (1); 120 m, Quesada Freddy *leg.*, 20.VI.1992, (2); **Puntarenas.** Carara, sendero Laguna Meándrica, 9.80544°N, 84.58399°W, 50 m, Guzmán Rosa *leg.*, 1.V.1994, (2); Cóbano, R.N.A. Cabo Blanco, Est San Miguel, 9.58892°N, 85.12397°W, 250 m, Porras Wendy *leg.*, 18.V.2001, (2); 300 m, Alvarado Francisco *leg.*, 27.X.1997, (1); Est. San Miguel, 3 Km. NW. de Cabo Blanco, 9.58121°N, 85.13670°W, 100 m, Ramírez Marvin *leg.*, 1.IX.1993, (38); 1.XI.1993, (6); Est. San Miguel, Sendero Maven, 9.63186°N, 85.12681°W, 300 m, Alvarado Francisco *leg.*, 29.X.1997, (58); Garabito, Res Biol Carara, Est Quebrada Bonita, 9.76745°N, 84.60811°W, 50 m, Bello Erick *leg.*, 1.VI.1990, (1); 1.VII.1990, (3); Guzmán Rosa *leg.*, 18.X.1992, (2); Saborío Juan Carlos *leg.*, 1.VII.1992, (1); 1.XI.1992, (1); Zúñiga Rodolfo *leg.*, 1.XI.1989, (1); 1.XII.1989, (1); 9.77423°N, 84.60812°W, 100 m, Guzmán Rosa *leg.*, 1.I.1994, (1); 1.IV.1995, (1); Garabito, Tárcoles, Cruce de quebradas, 9.77649°N, 84.60311°W, 0 m, Quesada Eddie *leg.*, 5.XI.1990, (1); Guacimal, Finca Buen Amigo Monteverde 4Km S. de la Reserva, 10.2767°N, 84.79661°W, 1040 m, Fuentes Zobeida *leg.*, 1.IX.1992, (1); Jacó, La Catarata, 9.57276°N, 84.59156°W, 75 m, Zumbado Manuel *leg.*, 24.XI.2008, (1); 26.XI.2008, (5); Los Ángeles. Paquera, 9.79028°N, 85.04518°W, 120 m, Alvarado Francisco *leg.*, 23.VI.1998, (10); Paquera, Rio Grande, 9.87133°N, 84.98519°W, 120 m, Alvarado Francisco *leg.*, 17.VI.1998, (4); Puntarenas, Isla San Lucas, Puesto Isla San Lucas, 9.94652°N, 84.90556°W, 50 m, Azofeifa José *leg.*, 13.IX.2005, (1); Puntarenas. Reserva Karen Mogensen, Sendero Principal, 9.86709°N, 85.05995°W, 315 m, Briceño Duvalier *leg.*, 23.VI.2003, (13); Porras Wendy *leg.*, 22.VI.2003, (2); 335 m, Cárdenas Yow *leg.*, 3.VII.2003, (1); 350 m, Cárdenas Yow *leg.*, 20.IX.2003, (2); 8.X.2003, (1); 400 m, Porras Wendy *leg.*, 24.VI.2003, (1); R. Priv. Karen Mogensen, Sendero Tres Ríos, 9.86314°N, 85.06274°W, 315 m, Briceño Duvalier *leg.*, 26.VI.2003, (1); 400 m, Porras Wendy *leg.*, 26.VI.2003, (1); R.N.A. Cabo Blanco, 9.59256°N, 85.11487°W, 300 m, Alvarado Francisco *leg.*, 16.V.1997, (4); Reserva Privada Karen Mogensen, Quebrada Pérez, 9.86437°N, 85.06496°W, 315 m, Cárdenas Yow *leg.*, 23.VI.2003, (8); Sector Laguna Meándrica., 9.79821°N, 84.58125°W, 100 m, Zúñiga Rodolfo *leg.*, 1.VI.1990, (2); San José, Est. Bijagual, 600 m. N de Bijagualito, 9.74308°N, 84.54476°W, 500 m, Saborío Juan Carlos *leg.*, 1.VII.1995, (1). **Panama (15). Bocas del Toro.** Almirante, 58 km norte de Boquete, 9.300°N, -82.400°W; Changuinola District, 9.250°N, -82.650°W. **Colón.** Alhajuela, en la mitad del trayecto entre Panamá y Colón, 9.2166°N, -79.6333°W; Montura Canoa, Extremo norte del Lago Gatún, 9.350°N, -79.800°W; Frijoles, 12 km WNW de Gamboa, 9.1666°N, -79.7916°W; Pipeline Road, 5 km NW de Gamboa, 9.150°N, -79.7166°W; Río Trinidad, Panamá, 9.0166°N, -79.9666°W. **Panamá.** Corozal, 3.5 km NW de Ancón Hill, 8.9833°N, -79.5666°W; Curundú, 3 km N de Ancón Hill, 8.9833°N, -79.55°W; Madden Forest, 9 km E de Gamboa, 9.100°N, -79.6166°W; Summit Park, 8 km SE de Gamboa, 9.0666°N, -79.650°W. **Panamá Oeste.** La Campana, 5.5 km WSW de Cermeno, 8.7166°N, -79.900°W; Fort Kobbe Army Reservation, 6.5 km SW de Ancón Hill, 8.900°N, -79.5833°W; Isla Barro Colorado, Panamá, 9.150°N, -79.850°W. **Veraguas.** 1.74 km al este de Paso Real, Panamá, Leg: A. Solís and C. Víquez, 8.1996°N, -81.2343°W.

**Diagnosis.** Dorsal surface ranges from completely glossy orange-yellow, to orange-yellow and green, to completely green, sometimes with blackish maculae on pronotum and/or elytra (Fig. 3C, D); somber head; pygidium orange-yellow; last abdominal ventrite bicolored, center black and lateral borders orange-yellow (Fig. 8A, B). Body length 6–10 mm. Clypeus with four teeth, two central clypeal teeth long and slender, slightly curved at their base (Fig. 3C, D). Ventral tooth of pronotal anterolateral border absent. Pronotal surface smooth, finely punctate. Eighth elytral stria with basal carina variable in length. Prescutellar impression variable in depth. Pygidium orange-yellow with fine punctuation, bearing no setae or very short and fine setae; with strong basal carina (Fig. 8C, D). Male pygidial length/width ratio 0.66 (Fig. 8).

**Remarks.** This species was originally described by von Harold (1863) from Granada, Nicaragua. Later, Schmidt (1922) reclassified it as a variety (ambiguous term of classical Linnean taxonomy for a heterogeneous group of phenomena, including nongenetic variation of the phenotype; Mayr and Ashlock 1991: 432) of *Canthon cyanellus* LeConte, 1859, without providing any explanation for this taxonomic decision. Such treatment was maintained by Robinson (1948), Halffter (1961, 2003), Howden (1966), and subsequent authors at a subspecific level. Robinson (1948: 96–97) considered that *C. cyanellus* reached Brazil and Peru and that the distinct subspecies could only be recognized by differences in coloration because no discrepancies in genitalia were apparent. Halffter (1961) proposed the following subspecies and varieties within *Canthon cyanellus*: *C. c. cyanellus* LeConte, 1859; *C. c. violetae* Halffter, 1961; *C. c. sallei* Harold, 1863, C. c. sallei var. triangulatus Schmidt, 1922; and C. c. sallei var. gutticollis Schmidt, 1922. Halffter (1961: 262), following Robinson (1948), recognized that the subspecies were delimited on how color variants were geographically structured. Halffter (1961) recognized three subspecies for *C. cyanellus* as geographical races. That is, allopatric populations distinguishable on the basis of genetically based phenotypic differences. Quoting Halffter (1961: 262): “changes in colouration correspond to defined geographical regions, with a gradual increase in the dark green parts from the south to the north”. In other words, Halffter’s polytypic classification of *C. cyanellus* was based on the assumption that color variation in the species was geographically structured; that is, the different color phena were not found sympatrically (or at least only over a narrow zone of intergradation). This was what he claimed his data showed in 1961. To complement this view, Halffter also enumerated subspecies-level (and, therefore, supposedly geographical) differences in the level of pronotal and elytral punctation, in the presence of the humeral carina, in the presence of the scutellar impression (in the key, he said instead the prescutellar impression), and the shape of the metatibiae. According to his taxonomy, the subspecies violetae was intermediate both geographically and phenotypically between *cyanellus*, to the north, and *sallei*, to the south.

This view started to collapse with the observation by Solís and Kohlmann (2002) that the color phenon typifying ssp. cyanellus, the northern (Mexican) subspecies, was also found sympatrically with the color phenon typifying ssp. sallei, the southern (Central American) subspecies, in some localities in Costa Rica, well beyond the supposed zone of intergradation in southern Mexico. The south-north phenotypic divide proposed by Halffter (1961) was shown to be at least partly incorrect— partly because Halffter (1961) was still correct in noting that orange, the sallei phenon, is, by far, the dominant phenon in Central America. So, it was wrong to treat the subspecies as monomorphic, but correct about the geographical divide in relative preponderance. Furthermore, because the *cyanellus* phenon was supposedly present in two disjunct portions of the range of the species, Mexico, to the north, and Costa Rica, to the south, Solís and Kohlmann (2002) correctly considered it polytopic. Finally, Halffter (2003) maintained this taxonomic point of view in his last taxonomic treatment of *C. cyanellus*.

In his doctoral thesis, Nunes (2019) primarily relied on color and gloss characteristics to validate *C. sallei* as a distinct species. Vaz-de-Mello labeled two *C. sallei* specimens, housed in the Natural History Museum, London (Fig. 9), as lectotype and paralectotype in 2014. However, the lectotype designation is invalid because it has not been published. Nunes (2019) also indicated that *Canthon dentiger* Harold, 1868 is a synonym of *C. sallei* and designated a lectotype from Mataruin, Colombia, which is housed at the MFNB – Museum für Naturkunde Berlin, Leibniz-Institut für Evolutions- und Biodiversitätsforschung, Berlin, Germany. This lectotype designation is also invalid because the work was not published. Nunes (2019) gives a detailed description of the external morphology and male genitalia of this species and considers *C. sallei* to be distributed from southern Chiapas in Mexico to northwestern Colombia, which is basically the same distribution ascribed to the subspecies sallei by Halffter (1961) (Fig. 10). Interestingly, *C. sallei* is not present in the Pacific tropical rainforest that spans south Costa Rica and northern Panama. Its niche may be taken by *Canthon mutabilis*, a species that lives in this area and also feeds on carrion. Nolasco-Soto et al. (2020, 2023) found that the morphological variation of the aedeagus does not align with the genetic-phylogeographic structure of this taxon, nor with the subspecies and varieties taxonomy based on color proposed by Halffter (1961).

Dorsal color analysis by Solís and Kohlmann (2002) of *C. sallei* in Costa Rica indicated that the same population could present different color patterns in a single locality at the same time, and that these same color differences had been used as a diagnostic character for identifying different taxa by Halffter (1961) (*C. sallei*, *C. violetae*). Solís and Kohlmann (2002: 12) did not recognize the existence of subspecies for *C. cyanellus* in their study based mainly on color differences as had been proposed by Halffter (1961) and correctly recognized at the time that the population of *sallei* was polytopic following the recommendation made by Mayr and Ashlock (1991: 100) that:

When subspecies of a species differ only in a single diagnostic character involving color, size, or pattern, several unrelated and more or less widely separated populations may independently develop an identical phenotype. The evolutionist knows that such populations are not identical genetically, but since the subspecies is not an evolutionary concept, taxonomists sometimes combine such visually identical populations into a single subspecific taxon. A geographically heterogeneous subspecies of this type is called a polytopic subspecies. Usually, it is preferable not to recognize polytopic subspecies. In the absence of diagnostic differences, there is no legitimate excuse for recognizing several subspecies merely on the basis of locality.

The present study has shown that *C. sallei* is no longer polytopic, but polymorphic. Polychromatism is not limited to Costa Rica, but to the rest of Central America and southern Mexico. The reason the color phena do not qualify as subspecies under Mayr’s definition is no longer because they are polytopic, but because they are now known not to represent geographical races. Being polytopic does not prevent a phenon from representing a geographical race. Actually, before 1969, Mayr argued for the recognition of polytopic race as subspecies in all of his writings, including in his 1963 seminal book *Animal Species and Evolution* (Mayr 1963: 388–389; 1969: 193). Therefore, if one adopts a definition of subspecies as geographical races with no polymorphisms, Halffter’s (1961) color-based polytypic classification is invalid, and the subspecific names must be synonymized. The difference between the Halffter (1961) and the Solis and Kohlmann views (2002) does not concern the species level or the existence of variation, but how to treat this variation taxonomically within the species: Halffter (1961) saw the variation as the basis for a polytypic classification, whereas Solís and Kohlmann (2002) saw it as the basis for a polytopic classification.

After further study and analysis of several distribution areas, external morphological differences (pronotal punctuation, pygidial length, punctuation, and setae; pygidial and last ventrite coloration) have been recognized by the present authors (AS, BK), allowing the separation of different taxa within the *C. cyanellus* species complex. Specimens from Panama, Costa Rica, Nicaragua, and Honduras have been examined and found to be distinct species from the populations commonly distributed in Mexico. The populations of these Central American areas correspond to what was described as *C. sallei* by von Harold (1863). We do not have specimens from South America, preventing morphological comparisons with taxa from that region. However, we analyzed mtDNA differences among the Central and South American populations which are also color polymorphic (unpublished data).

In their analysis of type specimens of South American dung beetles, Vaz-de-Mello and Cupello (2018) include a photograph of the lectotype of Canthon sallei var. triangulatus Schmidt, 1920 (Columbien, La Garita Gebirge, W. Frische), which Schmidt (1920) also cited (erroneously) to be present in Honduras and Costa Rica. The typical inverted triangular *macula* of the pronotum clearly seen in the photograph by Vaz-de-Mello and Cupello (2018: fig. 45) has not been found by us in Central America and is probably circumscribed in its distribution to South America. The same seems to hold for Canthon sallei var. guticollis Schmidt, 1920 cited only from Colombia, which Nunes considers to be a new synonym of the elevated *C. triangulatus*. In his doctoral thesis, Nunes (2019) recognized the existence of *C. triangulatus* Schmidt, 1920, from Brazil, Colombia, Trinidad and Tobago, and Venezuela and separated it from *C. sallei* using the pronotal mark and gloss in his identification key.

Regarding common taxa recorded for Mexico, Nunes (2019) lists *C. cyanellus*, *C. spinosus* (as *speciosus*), *C. violetae*, and a new species and subspecies, as well as a fringe penetration of *C. sallei* into southwestern Chiapas. *Canthon sallei* can be distinguished from *C. spinosus* (see the taxonomic history of *C. spinosus* below in the next species treatment). *Canthon cyanellus spinosus* has been treated as a junior synonym of *Canthon cyanellus cyanellus* since Halffter (1961) and is revalidated here), as already mentioned by von Harold (1868b), by its yellow pygidium with fine punctation and either absent or very short, fine setae (Fig. 8). In contrast, *C. spinosus* has a green or blue pygidium with an evident punctuation bearing short and thick setae (Fig. 11). *Canthon sallei* has the last abdominal ventrite black with an orange-yellow area of variable length at the extremes of the segment and none or very small setae (Fig. 8); in contrast, *C. spinosus* has a unicolor dark (not black) last abdominal ventrite with short thick setae (Fig. 11). The male pygidial length/width ratio of *C. sallei* is 0.66, whereas *C. spinosus* has a longer pygidium with a ratio of 0.72 (Figs 8, 11). Because of these differences in morphological characters, *C. sallei* is elevated back to its former species status. The characters used previously by Halffter (1961) of humeral elytral carina, scutellar impression, and pronotal punctuation are variable and as such do not make good taxonomic characters.

Nunes (2019: 64) presents in his doctoral thesis a key for distinguishing taxa within the *C. cyanellus* species group. However, in the first couplet, he mistakenly separates *C. cyanellus* and *C. spinosus* (as *speciosus*) from *C. sallei*, stating that the dorsal coloration of the first two species is uniformly green or blue. This is inaccurate, as *C. sallei*—as noted by Solís and Kohlmann (2002: 31)—exhibits a wide range of dorsal coloration, from yellow-reddish to entirely green. This variant can be easily separated because of its yellow pygidium versus a blue or green one in *C. cyanellus* or *C. spinosus*. In couplet 2 of his key, Nunes (2019: 64) mistakenly indicated that *C. sallei* does not have a carina at the eighth elytral stria; *C. sallei* has this carina, which varies in length.

*Canthon cyanellus violetae* was described by Halffter in 1961 based on one female specimen from the Pacific slope of Chiapas. Halffter (1961) based this subspecies on green colored elytra, a reddish-orange pronotum and pygidium, except at its blackish-green pygidial border. To try to clarify the relationship between both taxa, one of us (BK) tried to study the holotype, looking for it in the Halffter Collection that has now been incorporated into the IEXA Insect Collection “Dr. Miguel Ángel Morón Ríos”, Xalapa, Mexico. However, neither the curator nor BK could find the holotype or other specimens belonging to this taxon. Sadly, the curator had no information about the whereabouts of this holotype or additional material or if it had been loaned.

Nunes (2019) cites the three species (*C. sallei*, *C. violetae*, *C.* sp. nov.) coexisting in the same type locality of *C. violetae* of El Vergel, Motozintla, on the Pacific slope of Chiapas. However, the taxonomic situation between *C. sallei* and *C. violetae* has been resolved by Nolasco-Soto et al. (2023). In their fig. 9, they presented the results of color variation in relation to the formation of clades analyzed by a Bayesian consensus tree. In this analysis, Nolasco-Soto et al. (2023) obtained the SPS (southern Pacific slope) clade, comprising populations from El Vergel and Raymundo Enríquez. These populations, characterized by specimens with yellow pygidia, exhibit the full range of dorsal coloration—from green elytra, which Halffter (1961) and Nunes (2019) associated with *C. violetae*, to orange elytra, previously attributed to *C. sallei*. The coexistence of all these color forms in one locality at the same time had already been reported by Solís and Kohlmann (2002) for Costa Rica. We have found the same situation for Honduras in this study. Nolasco-Soto et al. (2023) interpreted this situation in Chiapas to be a possible hybridization zone between *C. sallei* and *C. violetae*, or that several codominant genes (sic, it should be epistasis, codominance is present in alleles) control color variation.

Our interpretation of the existence of the great color variation reported for the localities of *C. violetae* in El Vergel and Raymundo Enríquez by Nolasco-Soto et al. (2023: figs 3, 4, 9; table 2) is simpler. It is also consistent with one of the explanations invoked for *Bolbites* by Cupello et al. (2022: 1009), of a single polymorphic locus in an undivided deme (a single species). First, a previous study (Solís and Kohlmann 2002) and this one concerning Honduras have found the coexistence of specimens with green and orange elytra (representing *C. violetae* and *C. sallei*, respectively) in the same locality, at the same time. Second, the genetic-phylogeographic analysis by Nolasco-Soto et al. (2023: figs 3, 4, 9) shows that specimens with green and orange elytra from the same locality form the same genetic-phylogeographic clade (SPS). Considering that Nunes (2019) indicated the color of the elytra as the only difference between *C. sallei* and *C. violetae*, this leads to the conclusion that their two-subspecies/species taxonomy is wrong. We have a single polymorphic monotypic species. We agree with the comment made by Cupello et al. (2022: 1012):

For phanaeines at least, W. D. Edmonds’ works, which initially used the subspecies category but later abandoned it, have shown that a plethora of subspecies names previously established to refer to local colour variants have little explicative or operational value. They are, hence, best treated as junior synonyms of older names. This should be likely true for many other groups of dung beetles, as exemplified by Solís and Kohlmann (2002) and Nolasco-Soto et al. (2017, 2020) who independently demonstrated the artificiality of Halffter’s (1961) subspecies classification for the colour variants of *Canthon cyanellus* LeConte, 1859.

We thus consider that *C. violetae* is the junior synonym of *C. sallei*, and recommend that work conducted and published under the name of *C. violetae* should be reassigned to *C. sallei*.

Specimens from the Pacific slope stemming from the Halffter collection were also analyzed from Guerrero and Morelos in the Balsas River Depression and found to be a new taxon resembling *C. spinosus* and belonging to the *C. cyanellus* species group, as Nunes (2019) had already suggested. It awaits description as a new species.

##### *Canthon spinosus* Harold, 1863, reinstated (NR)

Fig. 3E

*Canthon speciosus* Harold, 1868, syn. nov.

Mexico, Guatemala, Belize, Honduras.

*Canthon spinosus*von Harold 1863, 1868, 1869, Bates 1887, Gillet 1911, Nunes 2019, Nolasco-Soto et al. 2023.

*Canthon spinosus* Harold, 1863: 174 (description, catalogue); Harold 1868: 42 (generic key); Gemminger and von Harold , 1869: 994 (catalogue); Bates 1887: 27 (catalogue); Gillet 1911: 29 (catalogue); Balthasar 1939a (taxonomy); Halffter 1961 (synonymy); Howden 1966 (synonymy); Nolasco-Soto et al. 2023: table 2 (phylogeography).

*Canthon speciosus*Harold 1868b: 41 (description and generic key); Harold 1869: 994 (catalogue); Bates 1887: 27 (revision of Central American Scarabaeinae); Gillet 1911: 29 (catalogue), Nolasco-Soto et al. 2023: table 2 (phylogeography).

*Canthon cyanellus speciosus* — Balthasar 1939b: 211 (new status); Balthasar 1951: 328 (catalogue); Halffter 1961: 262 (synonymy); Howden 1966: 727 (species list).

**Material examined (254). Honduras (4). Atlántida.** Tela, Los Olingos Lodge, 9/XI/2024, Cebo heces humanas, Bosque tropical húmedo, 20 m altura, 15°46'59" N, 87°38'9" W, José Daniel Rivera y Mauro Zavala cols. (4). **Mexico (250). Campeche.** Calakmul. Reserva de la Biósfera Calakmul. VII-14 18°19'49.13"N, 89°49'20.16"W Capello, M.V. & Escobar Hdez., F., col. (25). **Chiapas.** Lacanjá-Chansayab, 300 m, 6/VIII/1977, carroña en selva, col. B. Kohlmann (1); Yaxchilán 12.VII.81. E. Fuentes, col. Alt. 140 m. Selva *Brosimum* Necrotrampa (4). Palenque. Casa arquólogos. 17/VI/1993 G y V Halffter Col. Borde cebo-excremento 13–18 horas (13). **Oaxaca.** La Nueva Esperanza. 3-VIII-1980 C. Huerta, G. Quintero, E. Rivera Cols. (2), 14-V-80 140 m g Quintero, C. Castillo, M.L. Castillo, E. Rivera, Col. (2); Santa María Chimalapa, San Francisco La Paz. 21.VI. 2016. coprotrampa. 17°5'33.3"N, 94°9'52.6"W potrero 245 m Víctor Moctezuma Col. (51); 29,VI.2016 88 m. 17°5'29.9"N, 94°6'21.8"W (37). **Veracruz.** Palma Sola, 18–22/VIII/1976, D. Edmonds y B. Kohlmann cols. (7), 22/VI/1972, G. Halffter y P. Reyes cols. (7), 29/V/1975, P. Reyes, R. Blume, C. Huerta col., pastizal con restos de selva subcaducifolia al. 320 m, cebo pescado noche-día (1); Palma Sola, Barranca del Río Platanar, 22–31/VIII/1976 (3); Los Tuxtlas, 31/VIIII/1978, Est. Biol. Trop. UNAM, trampa pescado, M.A. Morón col. (7); Carretera a Actopan, Bocana pasando la desv. Ídolos, 20/IX/1994, L. Arellano y R. Sánchez cols., alt. 240 m., selva baja subcaducifolia, Trampa necro permanente (30); Presidio, F. Islas, VIII/1940 (6), IX/1940 (2); Tezonapa, F. Islas, 9/IV/1941(1). 700 m al NE de Apazapan NTP-80 Alt. 340 m 30.VII.92 Selva Baja cadu. Luis Quiroz, col. (45); Jalcomulco 17-Jul-90 Col. L. Arellano Selva Baja Alt 450 m NTP 80 (6). Rancho El Benjamín Nanchital IV-V-2016 Pastizal - B. tropical perennifolio J. Pelayo col. (76).

**Diagnosis.** Dorsal surface glossy green or glossy blue; somber head; pygidium dark blue or dark green; last abdominal ventrite unicolored, continuously somber (Fig. 3E). Body length 6–10 mm. Clypeus with four teeth, the two central clypeal teeth are slightly curved at their base (Fig. 3E). The ventral tooth of the pronotal anterolateral border absent. Pronotal surface smooth and finely punctured. Eighth elytral stria with basal carina almost reaching the basal elytral half. Prescutellar impression variable in depth. Pygidium green or blue with evident punctuation, bearing short and thick setae; with a strong basal carina (Fig. 11). Male pygidial length/width ratio 0.72.

**Remarks.***Canthon spinosus* was initially described by von Harold (1863) from southern Mexico. In 1868 Harold described *C. speciosus* based on a single female from Mexico too and indicated a certain closeness of this species to *C. sallei*. Later, Bates (1887: 27) mentioned *C. spinosus* from southern Mexico and *C. speciosus* from Guatemala and concluded that they are most probably the same species. Interestingly, Schmidt (1922) makes no mention of these species at all in his work. Balthasar (1939a: 211) considered *C. speciosus* (Mexico, Guatemala) to be a variety of *C. cyanellus* (USA) but made no mention of *C. spinosus*. Subsequently, Halffter (1961) synonymized *C. speciosus* and *C. spinosus* with *C. cyanellus cyanellus*. Later, Howden (1966: 727) followed suit and also considered *C. speciosus* and *C. spinosus* as synonyms of *C. c. cyanellus*. Finally, Nunes (2019: 73), in his doctoral thesis, considered *C. speciosus* a valid taxon at the species level and *C. spinosus* to be a senior synonym of *C. speciosus* without mentioning any specific reason.

Nunes (2019) separated *C. spinosus* (as *speciosus*) from the other taxa belonging to the *C. cyanellus* species group using dorsal color, the absence of a metafemoral posterior margin, and the pronotal surface. Further, *C. spinosus* has a smooth pronotal dorsal surface, while *C. cyanellus* has a rugous dorsal surface. Nunes (2019) gave a detailed description of the external morphology and male genitalia of this species and considered *C. spinosus* (as *speciosus*) to be distributed from central Veracruz, Mexico to Petén, Guatemala. Lectotypes were labeled for *C. speciosus* and *C. spinosus* by Vaz-de-Mello in 2014 at the Muséum National d’Histoire Naturelle, Paris, but as they have not been formally designated they remain syntypes. The morphological differences between *C. sallei* and *C. spinosus* are detailed in the section above treating *C. sallei*. Because of these differences in morphological characters, we elevate *Canthon spinosus* back to its former species status.

We disagree with Nunes (2019: fig. 16), however, that the beetle populations of the Yucatan Peninsula belong to *C. spinosus*. This is incorrect as they belong to a new related species that is awaiting description, and it differs from *C. spinosus* by having a longer pygidium and a smaller size. Capello and Halffter (2019) commented that the populations collected in Calakmul, Campeche (at the base of the Yucatan Peninsula), which they considered (erroneously, in our opinion) to pertain to *C. cyanellus*, presented two different size forms, one big and a small one, each actually corresponding, respectively, to *C. spinosus* and the new species. Both species meet in this locality, with the small species being the only one to advance further North into the Yucatan Peninsula. Nolasco-Soto et al. (2023) made the genetic-phylogeographic analysis of these populations and recovered two genetic-phylogeographic lines from Calakmul. One branch forms one lineage with specimens from Calakmul, Los Chimalapas, Oaxaca, and Palenque, Chiapas, in southeastern Mexico (*C. spinosus* considered as *C. speciosus* by Nolasco-Soto et al. 2023). In contrast, another lineage includes individuals from Calakmul and Chamela, Jalisco, located on the Pacific coast. This makes sense because tropical deciduous forests are present in Jalisco and Yucatan, and both areas likely had a common beetle and vegetation-type origin. Nolasco-Soto et al. (2023) reported a considerable number of genetic-phylogeographic lineages in their study of the *C. cyanellus* species complex from Mexico to Colombia; however, it is important to interpret them with caution, particularly in light of the geographic sampling gaps – such as data from Central America – and the absence of well-defined diagnostic characters observed in their earlier analyses (Nolasco-Soto et al. 2017, 2020, 2023). Moreover, if the individual dispersal range is short, a broken phylogeographic pattern can be expected (Cupello et al. 2022: 1020–1021). A taxonomic study of the *C. cyanellus* species group is underway and shall deal with this systematics situation in more detail.

According to Nunes (2019), *C. cyanellus* is approximately distributed from southern Texas to Papantla, in northern Veracruz. Nunes (2019) proposed the existence of a *C. cyanellus* subspecies from Tamaulipas because it has a green color, which, according to him, has only been found circumscribed to the locality of the biological reserve of “El Cielo” *versus* the blue *C. cyanellus* specimens. Actually, this green form had already been collected in other localities by the Mexican entomologists Federico Islas in 1941 in San Luis Potosí and Gonzalo Halffter in 1955 in Tamaulipas. Robinson (1948: 97) had already indicated that the Texas populations of *C. cyanellus* presented a mixture of green and blue specimens, so it comes as no surprise, as Nunes (2019: 67) tries to show in his study, the existence of two dorsal body colors, blue and green. We have found that *C. cyanellus* is distributed from Texas to Veracruz and the piedmont of Hidalgo and northern Puebla. Furthermore, according to Nunes (2019), the monochromatic species of this group found along the Atlantic coastline of Mexico (i.e., *C. cyanellus* and *C. spinosus*, formerly cited as *C. speciosus*) always show both a green and a blue form. This is part of color variation as can be found in *C. spinosus* and does not merit the creation of a subspecies. Color variation is here not geographically structured. As such, color variation does not delimit geographical races; it presents itself in the form of polymorphisms (i.e., as intrapopulation variation). *Canthon cyanellus* has a pronotal microsculpture that gives the impression of a creased surface; whereas *C. spinosus* has a smooth surface. It is interesting to note that *C. cyanellus* presents a very similar chorology as *Ateuchus texanus* (Robinson, 1948). *Ateuchus texanus* is distributed from central Texas, and as it enters Mexico, it moves into the piedmont of the Sierra Madre Oriental (Kohlmann 1984).

*Canthon spinosus* is distributed from central Veracruz to the northwestern Caribbean coast of Honduras. Accordingly, research conducted over the past 65 years on *C. cyanellus*—particularly studies based on material collected in central Veracruz (e.g., Palma Sola, the surroundings of Xalapa, Los Tuxtlas, Catemaco, Jalcomulco)— should be reattributed to *C. spinosus*, whose range encompasses these localities. This reassignment should be considered when citing findings from these studies in future research.

Finally, the species of the *C. cyanellus* species complex can be considered a superspecies. A superspecies has been defined by Mayr (1931) as a monophyletic group of closely related and largely or entirely allopatric species. Mayr (1931) introduced this English name as an international equivalent of the original German name, *Artenkreis*, coined by Rensch (1929), meaning a group of allopatric species, but mistranslated as “circle of species”. The component species of a superspecies is called an allospecies according to Amadon (1967). So we could indicate that the *cyanellus* superspecies is composed of *Canthon cyanellus*, *C. sallei*, *C. spinosus*, *C. triangulatus*, and three more new species that await description.

##### *Canthon vazquezae* Martínez, Halffter & Halffter, 1964 (RL, EZ)

*Glaphyrocanthon viridis vazquezae* Martínez, Halffter & Halffter, 1964.

Mexico, Honduras, Nicaragua, Costa Rica, Panama.

Martínez et al. 1964; Solís and Kohlmann 2002, 2012; Rivera-Cervantes and Halffter 1999; Creedy and Mann 2012.

##### *Deltochilum carrilloi* González & Vaz-de-Mello, 2015 (RL, EZ)

Mexico, Guatemala, Belize, Honduras.

González and Vaz-de-Mello 2015.

##### *Deltochilum lobipes* Bates, 1887 (CS, EZ)

Mexico, Guatemala, Belize, Honduras, El Salvador, Nicaragua, Costa Rica, Colombia.

Bates 1887; Howden 1966; Medina et al. 2001; Solís and Kohlmann 2012; González and Vaz-de-Mello 2015; Pablo-Cea et al. 2023.

**Material examined. Honduras (24): Comayagua**: Siguatepeque, Parque Nacional Cerro Azul Meámbar; 14°53'33"N, 87°51'21"W, 720 m; 15.VII.2023; JD Rivera leg.; pitfall trap, human dung; UNAH (1). **Gracias a Dios**: Brus Laguna, Reserva de Biosfera y el Hombre Río Plátano; 15°41'40"N, 84°33'43"W, 9 m; 8.VII.2023; JD Rivera and R Salomão leg.; pitfall trap, human dung; UNAH (1). Same locality; 15°42'28"N, 84°30'22"W, 2 m; 9.VII.2023; JD Rivera and R Salomão leg.; pitfall trap, human dung; UNAH (5). **Olancho**: Catacamas, Parque Nacional Sierra de Agalta; 14°55'1"N, 85°52'57"W, 586 m; 3.VII.2023; JD Rivera and R Salomão leg.; pitfall trap, human dung; UNAH (1). **Yoro**: Olanchito, Refugio Hábitat/Especie Colibrí Esmeralda Hondureño; 15°27'53"N, 86°52'52"W, 412 m; 29.VI.2023; JD Rivera and R Salomão leg.; pitfall trap, human dung; UNAH (1). Same locality; 15°27'9"N, 86°53'41"W, 338 m; 29.VI.2023; JD Rivera and R Salomão leg.; pitfall trap, human dung; UNAH (2). Same locality; 15°25'41"N, 86°53'38"W, 243 m; 30.VI.2023; JD Rivera and R Salomão leg.; pitfall trap, human dung; UNAH (5). Same locality; 15°25'57"N, 86°52'31"W, 255 m; 30.VI.2023; JD Rivera and R Salomão leg.; pitfall trap, human dung; UNAH (8).

##### *Deltochilum mexicanum* Burmeister, 1848 (CS, RL, EZ)

Mexico, Guatemala, Belize, Honduras, El Salvador, Nicaragua.

Burmeister 1848; Howden 1966; Creedy and Mann 2012; Maes et al. 2020; Pablo-Cea et al. 2023.

**Material examined. Honduras (1): Lempira**: Gracias, Parque Nacional Montaña de Celaque; 14°29'5"N, 88°40'11"W, 2030 m; 17.VI.2023; JD Rivera, R Salomão and M Michel leg.; pitfall trap, human dung; UNAH (1).

##### *Deltochilum pseudoparile* Paulian, 1938 (CS, RL, EZ)

Mexico, Honduras, Nicaragua, Costa Rica, Panama.

Paulian 1938; Howden 1966; Howden and Young 1981; Creedy and Mann 2012; Solís and Kohlmann 2012.

**Material examined. Honduras (1): Comayagua**: Siguatepeque, Parque Nacional Cerro Azul Meámbar; 14°52'21"N, 87°54'7"W, 993 m; 16.VII.2023; JD Rivera leg.; pitfall trap, human dung; UNAH (1).

##### *Deltochilum scabriusculum* Bates, 1887 (NR, CS)

Fig. 3F

*Deltochilum scabriusculum montanum* Howden, 1966.

Mexico, Guatemala, Belize, Honduras, El Salvador, Nicaragua, Costa Rica.

Bates 1887; Howden 1966; Génier 2012; Solís and Kohlmann 2012; Maes et al. 2020; Pablo-Cea et al. 2023.

**Material examined. Honduras (3): Olancho**: Catacamas, Parque Nacional Sierra de Agalta; 14°54'6"N, 85°52'25"W, 483 m; 3.VII.2023; JD Rivera and R Salomão leg.; pitfall trap, human dung; UNAH (2). **Yoro**: Olanchito, Refugio Hábitat/Especie Colibrí Esmeralda Hondureño; 15°27'9"N, 86°53'41"W, 338 m; 29.VI.2023; JD Rivera and R Salomão leg.; pitfall trap, human dung; UNAH (1).

##### *Malagoniella yucateca* (Harold, 1863) (NR, CS)

Fig. 4A

**Figure 4.**
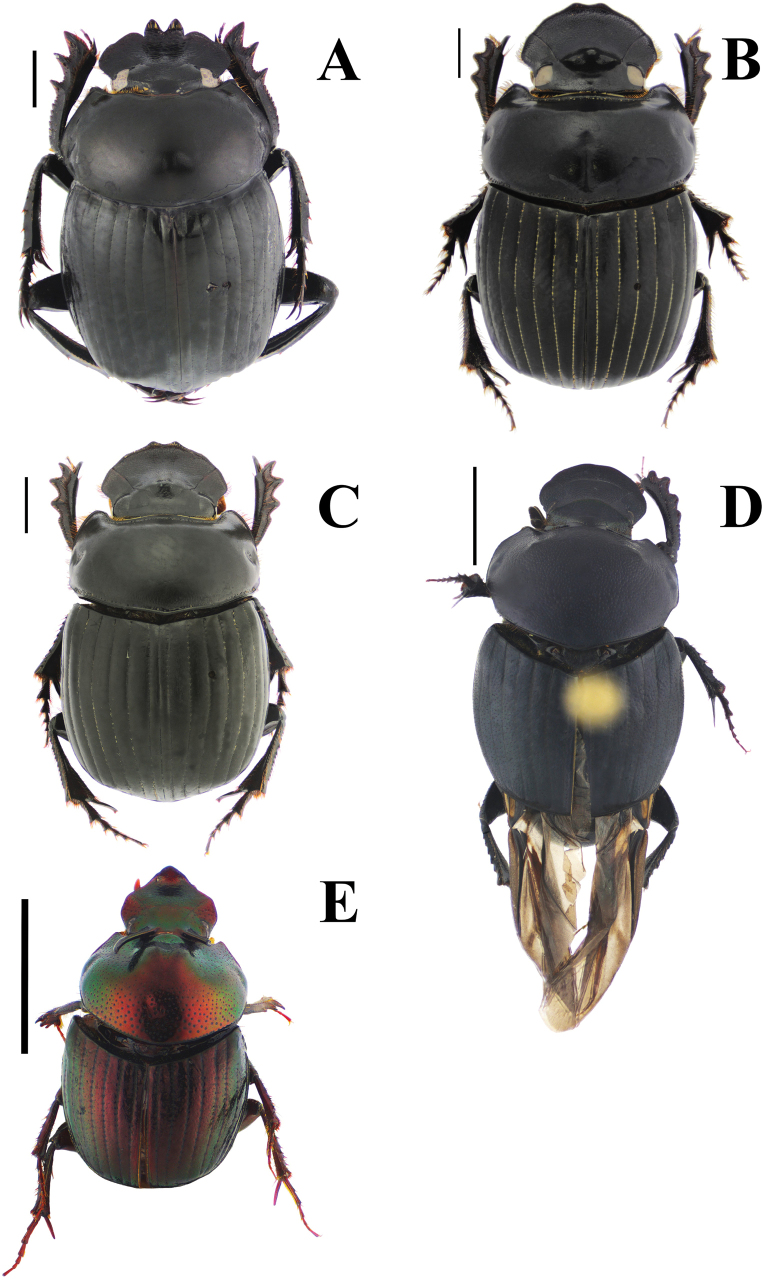
Habitus. A. *Malagoniella yucateca*, Refugio Hábitat/Especie Colibrí Esmeralda Hondureño (Olanchito), Departamento de Yoro; B. *Dichotomius enioi*, Parque Nacional Sierra de Agalta, Departamento de Olancho; C. *Dichotomius yucatanus*, Parque Nacional Cerro Azul Meambar, Departamento de Comayagua; Yoro; D. *Onthophagus salvadorensis*, Parque Nacional Montaña de Celaque, Departamento de Lempira; E. *Onthophagus yucatanus*, Parque Nacional Sierra de Agalta, Departamento de Olancho. Scale bar: 2 mm.

*Megathopa yucateca* Harold, 1863.

United States, Mexico, Honduras, El Salvador, Nicaragua, Costa Rica.

Harold 1863; Martínez 1961; Solís and Kohlmann 2012; Maes et al. 2020; Pablo-Cea et al. 2023.

**Material examined. Honduras (2): Yoro**: Olanchito, Refugio Hábitat/Especie Colibrí Esmeralda Hondureño; 15°27'53"N, 86°52'52"W, 412 m; 29.VI.2023; JD Rivera and R Salomão leg.; pitfall trap, human dung; UNAH (1). Same locality; 15°25'57"N, 86°52'31"W, 255 m; 30.VI.2023; JD Rivera and R Salomão leg.; pitfall trap, human dung; UNAH (1).

##### *Megathoposoma candezei* (Harold 1873) (NR, CS, EZ)

*Megathopa candezei* Harold, 1873.

*Megathoposoma deltochiloides* Balthasar 1939.

Mexico, Guatemala, Honduras, Nicaragua, Costa Rica, Panama.

Harold 1873; Wille 1973; Howden and Young 1981; Solís and Kohlmann 2012.

**Material examined. Honduras (1): Olancho**: Catacamas, Parque Nacional Sierra de Agalta; 14°55'1"N, 85°52'57"W, 586 m; 3.VII.2023; JD Rivera and R Salomão leg.; pitfall trap, human dung; UNAH (1).

##### *Pseudocanthon perplexus* (LeConte, 1847) (CS)

United States, Mexico, Guatemala, Honduras, El Salvador, Nicaragua, Costa Rica, Panama, Colombia, Venezuela, Brazil.

LeConte 1847; Howden and Young 1981; Solís and Kohlmann 2012; Maes et al. 2020; Capello and Halffter 2019; Pablo-Cea et al. 2023.

**Material examined. Honduras (43): Atlántida**: Tela, Parque Nacional Jeannette Kawas; 15°47'48"N, 87°33'21"W, 12 m; 18.VII.2024; JD Rivera leg.; pitfall trap, human dung; UNAH (2). **Gracias a Dios**: Brus Laguna, Reserva de Biosfera y el Hombre Río Plátano; 15°42'28"N, 84°30'22"W, 2 m; 9.VII.2023; JD Rivera and R Salomão leg.; pitfall trap, human dung; UNAH (9). **Olancho**: Catacamas, Aldea La Concepción; 14°45'57"N, 85°48'6"W, 320 m; 4.VII.2023; JD Rivera and R Salomão leg.; pitfall trap, human dung; UNAH (21). Same locality; 14°47'2"N, 85°45'32"W, 321 m; 4.VII.2023; JD Rivera and R Salomão leg.; pitfall trap, human dung; UNAH (11).

##### *Scybalocanthon moniliatus* (Bates, 1887) (NR, EZ)

*Canthon moniliatus* Bates, 1887.

Honduras, Nicaragua, Costa Rica, Panama, Peru, Brazil, Bolivia.

Bates 1887; Halffter and Martínez 1977; Howden and Young 1981; Solís and Kohlmann 2002, 2012; Silva and Valois 2019.

##### *Sylvicanthon aequinoctialis* (Harold, 1868) (CS, RL, EZ)

*Canthon aequinoctialis* Harold, 1868.

Honduras, Nicaragua, Costa Rica, Panama, Colombia.

Harold 1868b; Bates 1887; Howden and Young 1981; Solís and Kohlmann 2012; Cupello and Vaz-de-Mello 2018.

**Material examined. Honduras (132): Atlántida**: Tela, Parque Nacional Jeannette Kawas; 15°47'48"N, 87°33'21"W, 12 m; 18.VII.2024; JD Rivera leg.; pitfall trap, human dung; UNAH (42). **Gracias a Dios**: Brus Laguna, Reserva de Biosfera y el Hombre Río Plátano; 15°41'40"N, 84°33'43"W, 9 m; 8.VII.2023; JD Rivera and R Salomão leg.; pitfall trap, human dung; UNAH (33). Same locality; 15°42'45"N, 84°33'35"W, 13 m; 8.VII.2023; JD Rivera and R Salomão leg.; pitfall trap, human dung; UNAH (54). **Olancho**: Catacamas, Parque Nacional Sierra de Agalta; 14°54'6"N, 85°52'25"W, 483 m; 3.VII.2023; JD Rivera and R Salomão leg.; pitfall trap, human dung; UNAH (3).

#### ﻿Dichotomiini

##### *Dichotomius annae* Kohlmann & Solís, 1997 (CS, RL, EZ)

Mexico, Guatemala, Honduras, El Salvador, Nicaragua, Costa Rica, Panama.

Kohlmann and Solís 1997; Rivera and Cantarero 2011; Creedy and Mann 2012; Solís and Kohlmann 2012; Pablo-Cea et al. 2023.

**Material examined. Honduras (60): Comayagua**: Siguatepeque, Parque Nacional Cerro Azul Meámbar; 14°50'40"N, 87°57'12"W, 748 m; 15.VII.2023; JD Rivera leg.; pitfall trap, human dung; UNAH (1). Same locality; 14°52'50"N, 87°54'59"W, 712 m; 16.VII.2023; JD Rivera leg.; pitfall trap, human dung; UNAH (1). **Francisco Morazán**: Distrito Central, Parque Nacional La Tigra; 14°12'42"N, 87°8'51"W, 1464 m; 22.VI.2023; JD Rivera, R Salomão and M Michel leg.; pitfall trap, human dung; UNAH (1). Same locality; 14°12'42"N, 87°8'51"W, 1464 m; 22.VI.2023; JD Rivera, R Salomão and M Michel leg.; pitfall trap, human dung; UNAH (9). **Lempira**: Gracias, Parque Nacional Montaña de Celaque; 14°33'53"N, 88°37'54"W, 1271 m; 16.VI.2023; JD Rivera, R Salomão and M Michel leg.; pitfall trap, human dung; UNAH (15). Same locality; 14°29'5"N, 88°40'11"W, 2030 m; 17.VI.2023; JD Rivera, R Salomão and M Michel leg.; pitfall trap, human dung; UNAH (12). Same locality; 14°31'29"N, 88°39'2"W, 1650.5 m; 16.VI.2023; JD Rivera, R Salomão and M Michel leg.; pitfall trap, human dung; UNAH (17). **Olancho**: Catacamas, Parque Nacional Sierra de Agalta; 14°54'6"N, 85°52'25"W, 483 m; 3.VII.2023; JD Rivera and R Salomão leg.; pitfall trap, human dung; UNAH (1). Same locality; 14°55'1"N, 85°52'57"W, 586 m; 3.VII.2023; JD Rivera and R Salomão leg.; pitfall trap, human dung; UNAH (1). Catacamas, Aldea La Concepción; 14°47'2"N, 85°45'32"W, 321 m; 4.VII.2023; JD Rivera and R Salomão leg.; pitfall trap, human dung; UNAH (1). **Yoro**: Olanchito, Refugio Hábitat/Especie Colibrí Esmeralda Hondureño; 15°27'53"N, 86°52'52"W, 412 m; 29.VI.2023; JD Rivera and R Salomão leg.; pitfall trap, human dung; UNAH (1).

##### *Dichotomius chamuco* Solís & Kohlmann, 2025 (CS, EZ)

*Dichotomius satanas* (Harold, 1867)

Mexico, Guatemala, Honduras.

Rivera and Cantarero 2011; Creedy and Man 2012; Solís and Kohlmann 2025.

**Material examined. Honduras (48): Comayagua**: Siguatepeque, Parque Nacional Cerro Azul Meámbar; 14°50'40"N, 87°57'12"W, 748 m; 15.VII.2023; JD Rivera leg.; pitfall trap, human dung; UNAH (8). Same locality; 14°52'21"N, 87°54'7"W, 993 m; 16.VII.2023; JD Rivera leg.; pitfall trap, human dung; UNAH (7). Same locality; 14°52'50"N, 87°54'59"W, 712 m; 16.VII.2023; JD Rivera leg.; pitfall trap, human dung; UNAH (14). **Lempira**: Gracias, Parque Nacional Montaña de Celaque; 14°33'41"N, 88°39'8"W, 1622 m; 16.VI.2023; JD Rivera, R Salomão and M Michel leg.; pitfall trap, human dung; UNAH (5). Same locality; 14°33'53"N, 88°37'54"W, 1271 m; 16.VI.2023; JD Rivera, R Salomão and M Michel leg.; pitfall trap, human dung; UNAH (14).

##### *Dichotomius enioi* Montoya & Vaz-de-Mello, 2021 (NR, CS, EZ)

Fig. 4B

Mexico, Guatemala, Honduras.

Montoya and Vaz-de-Mello 2021; Solís and Kohlmann 2022.

**Material examined. Honduras (58): Comayagua**: Siguatepeque, Parque Nacional Cerro Azul Meámbar; 14°52'50"N, 87°54'59"W, 712 m; 16.VII.2023; JD Rivera leg.; pitfall trap, human dung; UNAH (15). **Olancho**: Catacamas, Parque Nacional Sierra de Agalta; 14°54'6"N, 85°52'25"W, 483 m; 3.VII.2023; JD Rivera and R Salomão leg.; pitfall trap, human dung; UNAH (7). Same locality; 14°55'1"N, 85°52'57"W, 586 m; 3.VII.2023; JD Rivera and R Salomão leg.; pitfall trap, human dung; UNAH (9). Catacamas, Aldea La Concepción; 14°45'57"N, 85°48'6"W, 320 m; 4.VII.2023; JD Rivera and R Salomão leg.; pitfall trap, human dung; UNAH (1). Same locality; 14°47'2"N, 85°45'32"W, 321 m; 4.VII.2023; JD Rivera and R Salomão leg.; pitfall trap, human dung; UNAH (6). **Yoro**: Olanchito, Refugio Hábitat/Especie Colibrí Esmeralda Hondureño; 15°27'53"N, 86°52'52"W, 412 m; 29.VI.2023; JD Rivera and R Salomão leg.; pitfall trap, human dung; UNAH (20).

##### *Dichotomius pisuicas* Solís & Kohlmann, 2025 (CS, EZ)

*Dichotomius satanas* (Harold, 1867)

Honduras, Nicaragua, Costa Rica, Panama.

Reyes Martínez and Suazo Euceda 2016; Solís and Kohlmann 2025.

**Material examined. Honduras (22): Olancho**: Catacamas, Parque Nacional Sierra de Agalta; 14°54'6"N, 85°52'25"W, 483 m; 3.VII.2023; JD Rivera and R Salomão leg.; pitfall trap, human dung; UNAH (5). Same locality; 14°55'1"N, 85°52'57"W, 586 m; 3.VII.2023; JD Rivera and R Salomão leg.; pitfall trap, human dung; UNAH (16). Catacamas, Aldea La Concepción; 14°47'2"N, 85°45'32"W, 321 m; 4.VII.2023; JD Rivera and R Salomão leg.; pitfall trap, human dung; UNAH (1).

##### *Dichotomius yucatanus* (Bates, 1887) (CS, RL, EZ)

Fig. 4C

*Pinotus yucatanus* Bates, 1887.

Mexico, Honduras, El Salvador, Nicaragua, Costa Rica.

Bates 1887; Howden and Young 1981; Kohlmann and Solís 1997; Kohlmann 2003; Solís and Kohlmann 2012; Maes et al. 2020.

**Material examined. Honduras (36): Francisco Morazán**: Distrito Central, Universidad Nacional Autónoma de Honduras – Ciudad Universitaria; 14°5'2"N, 87°11'32"W, 990 m; 20.VII.2024; JD Rivera leg.; pitfall trap, human dung; UNAH (20). **Olancho**: Catacamas, Aldea La Concepción; 14°45'57"N, 85°48'6"W, 320 m; 4.VII.2023; JD Rivera and R Salomão leg.; pitfall trap, human dung; UNAH (5). Same locality; 14°47'2"N, 85°45'32"W, 321 m; 4.VII.2023; JD Rivera and R Salomão leg.; pitfall trap, human dung; UNAH (2). **Yoro**: Olanchito, Refugio Hábitat/Especie Colibrí Esmeralda Hondureño; 15°27'53"N, 86°52'52"W, 412 m; 29.VI.2023; JD Rivera and R Salomão leg.; pitfall trap, human dung; UNAH (9).

#### ﻿Eurysternini

##### *Eurysternus angustulus* Harold, 1869 (NR, CS, EZ)

Mexico, Guatemala, Belize, Honduras.

von Harold 1869; Jessop 1985; Génier 2009.

**Material examined. Honduras (3): Olancho**: Catacamas, Parque Nacional Sierra de Agalta; 14°54'6"N, 85°52'25"W, 483 m; 3.VII.2023; JD Rivera and R Salomão leg.; pitfall trap, human dung; UNAH (1). Same locality; 14°55'1"N, 85°52'57"W; 3.VII.2023; 586 m; JD Rivera and R Salomão leg.; pitfall trap, human dung; UNAH (2).

##### *Eurysternus caribaeus* (Herbst, 1789) (CS, RL, EZ)

*Scarabaeus caribaeus* Herbst, 1789.

*Eurysternus peruanus* Harold, 1875; *Eurysternus nebulosus* Kirsch, 1871; *Eurysternus planus* Dalman, 1824.

Mexico, Guatemala, Belize, Honduras, Nicaragua, Costa Rica, Panama, Colombia, Venezuela, Guyana, Suriname, French Guiana, Ecuador, Peru, Brazil, Bolivia, Paraguay, Argentina, Trinidad and Tobago.

Herbst 1789; Howden and Young 1981; Jessop 1985; Génier 2009; Solís and Kohlmann 2012; Maes et al. 2020.

**Material examined. Honduras (11): Olancho**: Catacamas, Parque Nacional Sierra de Agalta; 14°54'6"N, 85°52′25"W, 483 m; 3.VII.2023; JD Rivera and R Salomão leg.; pitfall trap, human dung; UNAH (5). Same locality; 14°55'1"N, 85°52'57"W, 586 m; 3.VII.2023; JD Rivera and R Salomão leg.; pitfall trap, human dung; UNAH (6).

##### *Eurysternus foedus* Guérin-Méneville, 1830 (RL, EZ)

*Eurysternus claudicans* Kirsch, 1871.

Mexico, Guatemala, Belize, Honduras, Nicaragua, Costa Rica, Panama, Colombia, Venezuela, French Guiana, Ecuador, Peru, Brazil, Bolivia.

Guérin-Méneville 1844; Howden and Young 1981; Jessop 1985; Génier 2009; Creedy and Mann 2012; Maes et al. 2020.

##### *Eurysternus magnus* Castelnau, 1840 (CS, RL, EZ)

Mexico, Guatemala, Belize, Honduras, El Salvador, Nicaragua, Costa Rica, Panama.

Castelnau 1840; Jessop 1985; Génier 2009; Rivera and Cantarero 2011; Creedy and Mann 2012; Solís and Kohlmann 2012.

**Material examined. Honduras (9): Comayagua**: Siguatepeque, Parque Nacional Cerro Azul Meámbar; 14°52'21"N, 87°54'7"W, 993 m; 16.VII.2023; JD Rivera leg.; pitfall trap, human dung; UNAH (1). **Francisco Morazán**: Distrito Central, Parque Nacional La Tigra; 14°11'38"N, 87°8'34"W, 1439 m; 22.VI.2023; JD Rivera, R Salomão and M Michel leg.; pitfall trap, human dung; UNAH (1). Same locality; 14°12'42"N, 87°8'51"W, 1464 m; 22.VI.2023; JD Rivera, R Salomão and M Michel leg.; pitfall trap, human dung; UNAH (3). **Lempira**: Gracias, Parque Nacional Montaña de Celaque; 14°33'53"N, 88°37'54"W, 1271 m; 16.VI.2023; JD Rivera, R Salomão and M Michel leg.; pitfall trap, human dung; UNAH (2). Same locality; 14°29'5"N, 88°40'11"W, 2030 m; 17.VI.2023; JD Rivera, R Salomão and M Michel leg.; pitfall trap, human dung; UNAH (2).

##### *Eurysternus mexicanus* (Harold, 1869) (CS, RL, EZ)

Mexico, Guatemala, Belize, Honduras, El Salvador, Nicaragua, Costa Rica, Panama, Colombia, Venezuela, Guyana, Trinidad and Tobago.

von Harold 1869; Jessop 1985; Génier 2009; Creedy and Mann 2012; Solís and Kohlmann 2012; Pablo-Cea et al. 2023.

**Material examined. Honduras (4): Olancho**: Catacamas, Parque Nacional Sierra de Agalta ; 14°54'6"N, 85°52'25"W, 483 m; 3.VII.2023; JD Rivera and R Salomão leg.; pitfall trap, human dung; UNAH (1). Same locality; 14°55'1"N, 85°52'57"W, 586 m; 3.VII.2023; JD Rivera and R Salomão leg.; pitfall trap, human dung; UNAH (1). **Yoro**: Olanchito, Refugio Hábitat/Especie Colibrí Esmeralda Hondureño; 15°27'53"N, 86°52'52"W, 412 m; 29.VI.2023; JD Rivera and R Salomão leg.; pitfall trap, human dung; UNAH (1). Same locality; 15°25'41"N, 86°53'38"W, 243 m; 30.VI.2023; JD Rivera and R Salomão leg.; pitfall trap, human dung; UNAH (1).

##### *Eurysternus obliteratus* Génier, 2009 (RL)

Mexico, Guatemala, Belize, Honduras.

Génier 2009; Creedy and Mann 2012.

#### ﻿Oniticellini

##### *Euoniticellus intermedius* (Reiche, 1850)** (RL, EZ)

*Oniticellus intermedius* Reiche, 1850; *Oniticellus speciosus* Costa, 1853; *Oniticellus clavatus* Roth, 1851; *Oniticellus pallens* Laporte, 1840.

United States, Mexico, Guatemala, Honduras, El Salvador, Nicaragua, Costa Rica, Panama, Africa, Australia.

Reiche 1852; Montes de Oca et al. 1994; Solís and Kohlmann 2012; Rivera and Schlein 2014; Pablo-Cea et al. 2023.

#### ﻿Onthophagini

##### *Digitonthophagus gazella* (Fabricius, 1787)** (RL, EZ)

*Scarabaeus gazella* Fabricius, 1787; *Onthophagus intermedius* Reiche, 1840 (not established; ambiguous); *Copris antilope* Fabricius, 1798; *Copris metallicus* Fabricius, 1798; *Scarabaeus dorcas* Olivier, 1789.

United States, Hawai, Mexico, Guatemala, El Salvador, Honduras, Nicaragua, Panama, Colombia, Venezuela, Brazil, Peru, Bolivia, Argentina, Paraguay, Uruguay, Dominican Republic, Guadeloupe, Jamaica, Angola, Botswana, Comoros, Congo, Kenya, Madagascar, Malawi, Mozambique, Namibia, South, Africa, Swaziland, Tanzania, Uganda, Zambia, Zimbabwe, Australia, New Caledonia, Papua, New Guinea, Vanuatu.

Fabricius 1787; Balthasar 1959; Kohlmann 1994; Rivera and Cantarero 2011; Génier and Moretto 2017.

##### *Onthophagus acuminatus* Harold, 1880 (NR, CS, EZ)

Mexico, Honduras, El Salvador, Nicaragua, Costa Rica, Panama, Colombia, Ecuador, Peru, Brazil.

Harold 1880; Howden and Young 1981; Kohlmann and Solís 2001; Pulido and Zunino 2007; Maes et al. 2020.

**Material examined. Honduras (136): Comayagua**: Siguatepeque, Parque Nacional Cerro Azul Meámbar; 14°50'40"N, 87°57'12"W, 748 m; 15.VII.2023; JD Rivera leg.; pitfall trap, human dung; UNAH (1). Same locality; 14°52'21"N, 87°54'7"W, 993 m; 16.VII.2023; JD Rivera leg.; pitfall trap, human dung; UNAH (5). Same locality; 14°52'50"N, 87°54'59"W, 712 m; 16.VII.2023; JD Rivera leg.; pitfall trap, human dung; UNAH (29). **Gracias a Dios**: Brus Lagunas, Reserva de Biosfera y el Hombre Río Plátano; 15°41'40"N, 84°33'43"W, 9 m; 8.VII.2023; JD Rivera and R Salomão leg.; pitfall trap, human dung; UNAH (69). Same locality; 15°42'45"N, 84°33'35"W, 13 m; 8.VII.2023; JD Rivera and R Salomão leg.; pitfall trap, human dung; UNAH (27). Same locality; 15°42'28"N, 84°30'22"W, 2 m; 9.VII.2023; JD Rivera and R Salomão leg.; pitfall trap, human dung; UNAH (1). **Olancho**: Catacamas, Aldea La Concepción; 14°45'57"N, 85°48'6"W, 320 m; 4.VII.2023; JD Rivera and R Salomão leg.; pitfall trap, human dung; UNAH (4).

##### *Onthophagus anthracinus* Harold, 1873 (NR, CS, RL)

Mexico, Guatemala, Honduras, Nicaragua, Costa Rica, Panama.

Harold 1873; Bates 1887; Pittier and Biolley 1895; Howden and Cartwright 1963; Kohlmann and Solís 2001; Pulido and Zunino 2007; Hernández et al. 2024b.

**Material examined. Honduras (1): Lempira**: Gracias, Parque Nacional Montaña de Celaque; 14°33'53"N, 88°37'54"W, 1271 m; 16.VI.2023; JD Rivera, R Salomão and M Michel leg.; pitfall trap, human dung; UNAH (1).

##### *Onthophagus batesi* Howden & Cartwright, 1963 (CS, RL, EZ)

United States, Mexico, Guatemala, Belize, Honduras, El Salvador, Nicaragua, Costa Rica, Panama, Colombia.

Howden and Cartwright 1963; Kohlmann and Solís 2001; Medina et al. 2001; Pulido and Zunino 2007; Rivera and Cantarero 2011; Solís and Kohlmann 2012.

**Material examined. Honduras (165): Atlántida**: Tela, Parque Nacional Jeannette Kawas; 15°47'48"N, 87°33'21"W, 12 m; 18.VII.2024; JD Rivera leg.; pitfall trap, human dung; UNAH (114). **Francisco Morazán**: Distrito Central, Universidad Nacional Autónoma de Honduras – Ciudad Universitaria; 14°5'2"N, 87°11'32"W, 990 m; 20.VII.2024; JD Rivera leg.; pitfall trap, human dung; UNAH (35). **Gracias a Dios**: Brus Lagunas, Reserva de Biosfera y el Hombre Río Plátano; 15°42'45"N, 84°33'35"W, 13 m; 8.VII.2023; JD Rivera and R Salomão leg.; pitfall trap, human dung; UNAH (3). **Olancho**: Catacamas, Parque Nacional Sierra de Agalta; 14°54'6"N, 85°52'25"W, 483 m; 3.VII.2023; JD Rivera and R Salomão leg.; pitfall trap, human dung; UNAH (2). Catacamas, Aldea La Concepción; 14°45'57"N, 85°48'6"W, 320 m; 4.VII.2023; JD Rivera and R Salomão leg.; pitfall trap, human dung; UNAH (2). Same locality; 14°47'2"N, 85°45'32"W, 321 m; 4.VII.2023; JD Rivera and R Salomão leg.; pitfall trap, human dung; UNAH (9).

##### *Onthophagus breviconus* Génier & Howden, 1999* (RL, EZ)

Guatemala, Honduras.

Génier and Howden 1999; Creedy and Mann 2012.

##### *Onthophagus championi* Bates, 1887 (CS, RL, EZ)

Mexico, Guatemala, Honduras, El Salvador, Nicaragua, Costa Rica.

Bates 1887; Howden and Cartwright 1963; Kohlmann and Solís 2001; Pulido and Zunino 2007; Rivera and Cantarero 2011; Solís and Kohlmann 2012; Moctezuma and Halffter 2021b.

**Material examined. Honduras (103): Yoro**: Olanchito, Refugio Hábitat/Especie Colibrí Esmeralda Hondureño; 15°27'53"N, 86°52'52"W, 412 m; 29.VI.2023; JD Rivera and R Salomão leg.; pitfall trap, human dung; UNAH (15). Same locality; 15°27'9"N, 86°53'41"W, 338 m; 29.VI.2023; JD Rivera and R Salomão leg.; pitfall trap, human dung; UNAH (34). Same locality; 15°25'41"N, 86°53'38"W, 243 m; 30.VI.2023; JD Rivera and R Salomão leg.; pitfall trap, human dung; UNAH (16). Same locality; 15°25'57"N, 86°52'31"W, 255 m; 30.VI.2023; JD Rivera and R Salomão leg.; pitfall trap, human dung; UNAH (38).

##### *Onthophagus crinitus* Harold, 1869 (NR, CS, EZ)

Mexico, Guatemala, Belize, Honduras, El Salvador, Nicaragua, Costa Rica, Panama, Colombia.

von Harold 1869; Howden and Young 1981; Kohlmann and Solís 2001; Medina et al. 2001; Pulido and Zunino 2007; Solís and Kohlmann 2012; Maes et al. 2020; Pablo-Cea et al. 2023.

**Material examined. Honduras (68): Gracias a Dios**: Brus Lagunas, Reserva de Biosfera y el Hombre Río Plátano; 15°41'40"N, 84°33'43"W, 9 m; 8.VII.2023; JD Rivera and R Salomão leg.; pitfall trap, human dung; UNAH (64). Same locality; 15°42'45"N, 84°33'35"W, 13 m; 8.VII.2023; JD Rivera and R Salomão leg.; pitfall trap, human dung; UNAH (4).

##### *Onthophagus cyanellus* Bates, 1887 (CS, RL, EZ)

*Onthophagus mesoamericanus* Zunino & Halffter, 1988.

Mexico, Guatemala, Honduras, El Salvador, Nicaragua, Costa Rica, Panama.

Bates 1887; Pittier and Biolley 1895; Kohlmann and Solís 2001; Pulido and Zunino 2007; Creedy and Mann 2012; Kohlmann and Solís 2001, 2012; Maes et al. 2020; Pablo-Cea et al. 2023.

**Material examined. Honduras (2): Francisco Morazán**: Distrito Central, Parque Nacional La Tigra; 14°12'42"N, 87°8'51"W, 1464 m; 22.VI.2023; JD Rivera, R Salomão and M Michel leg.; pitfall trap, human dung; UNAH (2).

##### *Onthophagus gazellinus* Bates, 1887 (NR, EZ)

Honduras, Nicaragua, Costa Rica.

Bates 1887; Kohlmann and Solís 2001, 2012; Maes et al. 2020.

##### *Onthophagus hoepfneri* Harold, 1869 (CS, RL, EZ)

*Onthophagus arizonensis* Schaeffer, 1909.

United States, Mexico, El Salvador, Honduras, Nicaragua, Costa Rica.

von Harold 1869; Howden and Cartwright 1963; Kohlmann and Solís 2001; Rivera and Cantarero 2011; Maes et al. 2020; Pablo-Cea et al. 2023.

##### *Onthophagus incensus* Say, 1835 (CS, RL, EZ)

United States, Mexico, Guatemala, Honduras, El Salvador, Nicaragua, Costa Rica, Panama, Colombia, Venezuela, Ecuador.

Say 1835; Bates 1887; Pittier and Biolley 1895; Howden and Cartwright 1963; Kohlmann and Solís 2001; Pulido and Zunino 2007; Rivera and Cantarero 2011; Solís and Kohlmann 2012; Maes et al. 2020; Pablo-Cea et al. 2023.

**Material examined. Honduras (13): Francisco Morazán**: Distrito Central, Parque Nacional La Tigra; 14°11'38"N, 87°8'34"W, 1439 m; 22.VI.2023; JD Rivera, R Salomão and M Michel leg.; pitfall trap, human dung; UNAH (5). Same locality; 14°12'42"N, 87°8'51"W, 1464 m; 22.VI.2023; JD Rivera, R Salomão and M Michel leg.; pitfall trap, human dung; UNAH (6). **Lempira**: Gracias, Parque Nacional Montaña de Celaque; 14°33'53"N, 88°37'54"W, 1271 m; 16.VI.2023; JD Rivera, R Salomão and M Michel leg.; pitfall trap, human dung; UNAH (1). Same locality; 14°29'5"N, 88°40'11"W, 2030 m; 17.VI.2023; JD Rivera, R Salomão and M Michel leg.; pitfall trap, human dung; UNAH (1).

##### *Onthophagus landolti* Harold, 1880 (CS, RL, EZ)

*Onthophagus texanus* Schaeffer, 1914.

United States, Mexico, Guatemala, Belize, Honduras, El Salvador, Nicaragua, Costa Rica, Panama, Colombia, Venezuela.

Harold 1880; Pittier and Biolley 1895; Howden and Cartwright 1963; Howden and Young 1981; Medina et al. 2001; Pulido and Zunino 2007; Rivera and Cantarero 2011; Solís and Kohlmann 2012; Capello and Halffter 2019; Pablo-Cea et al. 2023.

**Material examined. Honduras (888): Francisco Morazán**: Distrito Central, Universidad Nacional Autónoma de Honduras – Ciudad Universitaria; 14°5'2"N, 87°11'32"W, 990 m; 20.VII.2024; JD Rivera leg.; pitfall trap, human dung; UNAH (20). **Lempira**: Gracias, Parque Nacional Montaña de Celaque; 14°29'5"N, 88°40'11"W, 2030 m; 17.VI.2023; JD Rivera, R Salomão and M Michel leg.; pitfall trap, human dung; UNAH (1). **Yoro**: Olanchito, Refugio Hábitat/Especie Colibrí Esmeralda Hondureño; 15°27'53"N, 86°52'52"W, 412 m; 29.VI.2023; JD Rivera and R Salomão leg.; pitfall trap, human dung; UNAH (220). Same locality; 15°27'9"N, 86°53'41"W, 338 m; 29.VI.2023; JD Rivera and R Salomão leg.; pitfall trap, human dung; UNAH (309). Same locality; 15°25'41"N, 86°53'38"W, 243 m; 30.VI.2023; JD Rivera and R Salomão leg.; pitfall trap, human dung; UNAH (114). Same locality; 15°25'57"N, 86°52'31"W, 255 m; 30.VI.2023; JD Rivera and R Salomão leg.; pitfall trap, human dung; UNAH (224).

##### *Onthophagus longimanus* Bates, 1887 (RL)

Mexico, Guatemala, Belize, Honduras.

Bates 1887; Pulido and Zunino 2007; Creedy and Mann 2012.

##### *Onthophagus nyctopus* Bates, 1887 (NR, EZ)

Honduras, Nicaragua, Costa Rica, Colombia, Perú.

Bates 1887; Kohlmann and Solís 2001; Medina et al. 2001; Hernández et al. 2024b.

##### *Onthophagus petenensis* Howden & Gill, 1993 (NR, RL)

Mexico, Guatemala, Belize, Honduras.

Howden and Gill 1993; Pulido and Zunino 2007; Creedy and Mann 2012.

##### *Onthophagus praecellens* Bates, 1887 (NR, CS)

Guatemala, Honduras, Nicaragua, Costa Rica, Panama, Colombia, Trinidad and Tobago.

Bates 1887; Pittier and Biolley 1895; Kohlmann and Solís 2001; Medina et al. 2001; Pulido and Zunino 2007; Solís and Kohlmann 2012; Maes et al. 2020.

**Material examined. Honduras (6): Comayagua**: Siguatepeque, Parque Nacional Cerro Azul Meámbar; 14°52'21"N, 87°54'7"W, 993 m; 16.VII.2023; JD Rivera leg.; pitfall trap, human dung; UNAH (6).

##### *Onthophagus rhinolophus* Harold, 1869 (CS, RL, EZ)

Mexico, Guatemala, Honduras, Nicaragua.

von Harold 1869; Bates 1887; Pulido and Zunino 2007; Capello and Halffter 2019; Maes et al. 2020.

**Material examined. Honduras (22): Gracias a Dios**: Brus Lagunas, Reserva de Biosfera y el Hombre Río Plátano; 15°41'40"N, 84°33'43"W, 9 m; 8.VII.2023; JD Rivera and R Salomão leg.; pitfall trap, human dung; UNAH (1). **Olancho**: Catacamas, Parque Nacional Sierra de Agalta; 14°54'6"N, 85°52'25"W, 483 m; 3.VII.2023; JD Rivera and R Salomão leg.; pitfall trap, human dung; UNAH (6). Same locality; 14°55'1"N, 85°52'57"W, 586 m; 3.VII.2023; JD Rivera and R Salomão leg.; pitfall trap, human dung; UNAH (15).

##### *Onthophagus salvadorensis* Zunino & Halffter, 1988 (NR, CS)

Fig. 4D

Honduras, El Salvador.

Zunino and Halffter 1988; Pulido and Zunino 2007; Pablo-Cea et al. 2023.

**Material examined. Honduras (2): Lempira**: Gracias, Parque Nacional Montaña de Celaque; 14°33'41"N, 88°39'8"W, 1622 m; 16.VI.2023; JD Rivera, R Salomão and M Michel leg.; pitfall trap, human dung; UNAH (2).

**Remarks.** As in the case of *Haplogeotrupes guatemalensis tridentatus*, Howden collected this species for the first time at 2300 m a.s.l. in Cerro Monte Cristo in El Salvador in 1971. Fifty-three years elapsed before we collected it again. It is also the first time this species has been collected in Honduras. As in the case of the geotrupids, this species was collected in the Parque Nacional de Montaña Celaque at 1560 m a.s.l. This is the southernmost known record for both species.

According to Zunino and Halffter (1988), this species belongs to the *O. cyanellus* species line, which is considered an element of ancient northern origin. Its diversification is believed to have occurred in ancient geological times, as evidenced by its distinct morphology, distribution, and ecological requirements (Zunino and Halffter 1988). This proposal aligns with the origin of the cloud forest habitat where this species resides. The cloud forest is believed to have an ancient Oligocene-Miocene origin (Rzedowski 1996). This vegetation type has also been recognized as an important center for plant and animal diversification, as well as an endemism center (Flores and Navarro 1993).

##### *Onthophagus* sp.* (RL)

Honduras.

Creedy and Mann 2012.

##### *Onthophagus stockwelli* Howden & Young, 1981 (NR, EZ)

Honduras, Nicaragua, Costa Rica, Panama, Colombia, Ecuador.

Howden and Young 1981; Kohlmann and Solís 2001; Pulido and Zunino 2007; Solís and Kohlmann 2012; Hernández et al. 2024b.

##### *Onthophagus viridivinosus* Kohlmann & Solis, 2001 (NR, CS)

Honduras, Nicaragua, Costa Rica.

Kohlmann and Solís 2001; Pulido and Zunino 2007; Solís and Kohlmann 2012; Hernández et al. 2024b.

**Material examined. Honduras (11): Gracias a Dios**: Brus Lagunas, Reserva de Biosfera y el Hombre Río Plátano; 15°42'45"N, 84°33'35"W, 13 m; 8.VII.2023; JD Rivera and R Salomão leg.; pitfall trap, human dung; UNAH (4). **Olancho**: Catacamas, Parque Nacional Sierra de Agalta; 14°55'1"N, 85°52'57"W, 586 m; 3.VII.2023; JD Rivera and R Salomão leg.; pitfall trap, human dung; UNAH (7).

##### *Onthophagus yucatanus* Delgado, Peraza & Deloya, 2006 (NR, CS)

Fig. 4E

Mexico, Guatemala, Belize, Honduras.

Delgado et al. 2006; Pulido and Zunino 2007; Capello and Halffter 2019.

**Material examined. Honduras (23): Atlántida**: Tela, Los Olingos Lodge; 15°46'59"N, 87°38'9"W, 20 m; 9.XI.2024; JD Rivera leg.; pitfall trap, human dung; UNAH (4). **Comayagua**: Siguatepeque, Parque Nacional Cerro Azul Meámbar; 14°52'50"N, 87°54'59"W, 712 m; 16.VII.2023; JD Rivera leg.; pitfall trap, human dung; UNAH (9). **Olancho**: Catacamas, Parque Nacional Sierra de Agalta; 14°54'6"N, 85°52'25"W, 483 m; 3.VII.2023; JD Rivera and R Salomão leg.; pitfall trap, human dung; UNAH (1). Same locality; 14°55'1"N, 85°52'57"W, 586 m; 3.VII.2023; JD Rivera and R Salomão leg.; pitfall trap, human dung; UNAH (5). **Yoro**: Olanchito, Refugio Hábitat/Especie Colibrí Esmeralda Hondureño; 15°27'53"N, 86°52'52"W, 412 m; 29.VI.2023; JD Rivera and R Salomão leg.; pitfall trap, human dung; UNAH (4).

#### ﻿Phanaeini

##### *Coprophanaeus boucardi* (Nevinson, 1891) (RL, EZ)

*Phanaeus boucardi* Nevinson, 1891; *Coprophanaeus pluto boucardi* (Nevinson); Arnaud 2002.

Mexico, Honduras, El Salvador, Nicaragua, Costa Rica.

Nevinson 1891; Edmonds and Zídek 2010; Sánchez-Hernández et al. 2019.

##### *Coprophanaeus corythus* (Harold, 1863) (CS, RL, EZ)

*Phanaeus corythus* Harold, 1863; *Phanaeus perseus* Harold, 1880; *Phanaeus telamon corythus* (Harold); *Coprophanaeus telamon corythus* (Harold); *Coprophanaeus telamon nevinsoni* Arnaud and Gámez 2002; Pereira and Martínez 1956; Arnaud 2002.

Mexico, Guatemala, Belize, Honduras, El Salvador, Nicaragua, Costa Rica, Panama, Colombia, Venezuela.

Harold 1863; Edmonds and Zídek 2010; Creedy and Mann 2012; Solís and Kohlmann 2012; Maes et al. 2020; Pablo-Cea et al. 2023.

**Material examined. Honduras (50): Comayagua**: Siguatepeque, Parque Nacional Cerro Azul Meámbar; 14°50'40"N, 87°57'12"W, 748 m; 15.VII.2023; JD Rivera leg.; pitfall trap, human dung; UNAH (2). Same locality; 14°52'50"N, 87°54'59"W, 712 m; 16.VII.2023; JD Rivera leg.; pitfall trap, human dung; UNAH (2). Same locality; 14°51'45"N, 87°55'48"W, 730 m; 15.VII.2023; JD Rivera leg.; pitfall trap, human dung; UNAH (4). **Gracias a Dios**: Brus Lagunas, Reserva de Biosfera y el Hombre Río Plátano; 15°41'40"N, 84°33'43"W, 9 m; 8.VII.2023; JD Rivera and R Salomão leg.; pitfall trap, human dung; UNAH (9). Same locality; 15°42'45"N, 84°33'35"W, 13 m; 8.VII.2023; JD Rivera and R Salomão leg.; pitfall trap, human dung; UNAH (12). Same locality; 15°42'28"N, 84°30'22"W, 2 m; 9.VII.2023; JD Rivera and R Salomão leg.; pitfall trap, human dung; UNAH (1). Same locality; 15°42'26"N, 84°32'33"W, 8 m; 8.VII.2023; JD Rivera and R Salomão leg.; pitfall trap, human dung; UNAH (14). **Olancho**: Catacamas, Parque Nacional Sierra de Agalta; 14°55'1"N, 85°52'57"W, 586 m; 3.VII.2023; JD Rivera and R Salomão leg.; pitfall trap, human dung; UNAH (4). Catacamas, Aldea la Concepción; 14°47'2"N, 85°45'32"W, 321 m; 4.VII.2023; JD Rivera and R Salomão leg.; pitfall trap, human dung; UNAH (2).

##### *Coprophanaeus gilli* Arnaud, 1997 (RL, EZ)

*Coprophanaeus henryi* Malý & Pokorný, 2008.

Mexico, Guatemala, Belize, Honduras.

Arnaud 1997; Arnaud 2002b; Edmonds and Zídek 2010; Creedy and Mann 2012; Kohlmann and Solís 2012.

##### *Coprophanaeus kohlmanni* Arnaud, 2002 (EZ)

Honduras, Nicaragua, Costa Rica, Panama.

Arnaud 2002b; Edmonds and Zídek 2010; Kohlmann and Solís 2012.

##### *Phanaeus endymion* Harold, 1863 (CS, RL)

Fig. 5A

**Figure 5.**
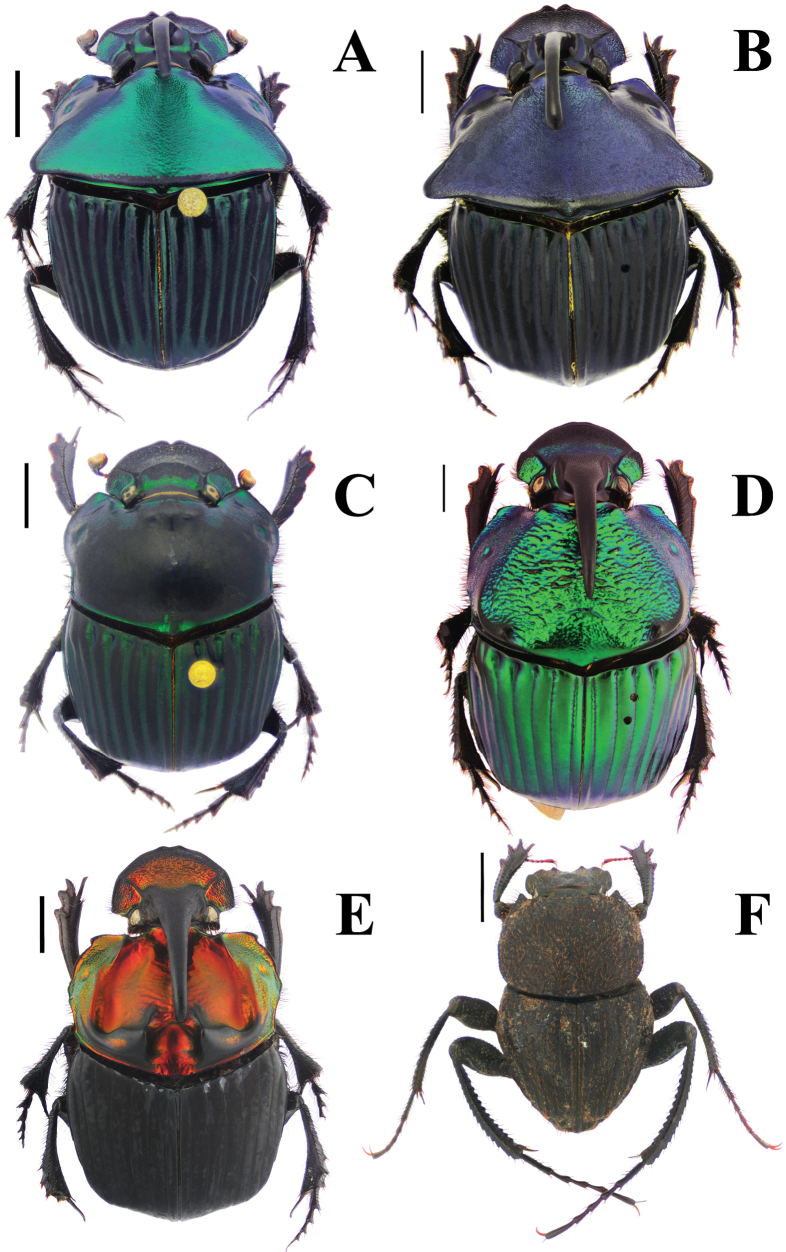
Habitus. A. *Phanaeus endymion*, Valle de Ángeles, Departamento de Francisco Morazán; B. *Phanaeus porioni* (♂) Refugio Hábitat/Especie Colibrí Esmeralda Hondureño (Olanchito), Departamento de Yoro; C. *Phanaeus porioni* (♀), Refugio Hábitat/Especie Colibrí Esmeralda Hondureño (Olanchito), Departamento de Yoro; D. *Phanaeus wagneri*, Refugio Hábitat/Especie Colibrí Esmeralda Hondureño (Olanchito), Departamento de Yoro; E. *Sulcophanaeus chryseicollis* (♂), Parque Nacional Sierra de Agalta, Departamento de Olancho; F. *Sisyphus mexicanus*, Refugio Hábitat/Especie Colibrí Esmeralda Hondureño (Olanchito), Departamento de Yoro. Scale bar: 2 mm.

Mexico, Guatemala, Belize, Honduras.

Harold 1863; Edmonds 1994; Rivera and Cantarero 2011; Creedy and Mann 2012; Edmonds and Zídek 2012; Moctezuma and Halffter 2021a; Moctezuma and Vaz-de-Mello 2021.

**Material examined. Honduras (5): Comayagua**: Siguatepeque, Parque Nacional Cerro Azul Meámbar; 14°53'33"N, 87°51'21"W, 720 m; 15.VII.2023; JD Rivera leg.; pitfall trap, human dung; UNAH (2). Same locality; 14°52'21"N, 87°54'7"W, 993 m; 16.VII.2023; JD Rivera leg.; pitfall trap, human dung; UNAH (3).

##### *Phanaeus excelsus* Bates, 1889 (RL)

Guatemala, El Salvador, Honduras, Nicaragua, Costa Rica.

Bates 1889; Edmonds 1994; Rivera and Cantarero 2011.

##### *Phanaeus eximius* Bates 1889 (RL, EZ)

Guatemala, El Salvador, Honduras, Nicaragua, Costa Rica.

Bates 1889; Edmonds 1994.

###### ﻿ Phanaeus lempira

Taxon classificationAnimaliaColeopteraScarabaeidae

Kohlmann, Solís & Rivera sp. nov.

11E2387E-69E4-5880-B510-854B2DA2D54E

https://zoobank.org/99D4D7FF-F740-4BBC-96C7-3849E710402E

Figs 6, 7

####### Note.

(CS, EZ); Honduras, Mexico.

**Figure 6.**
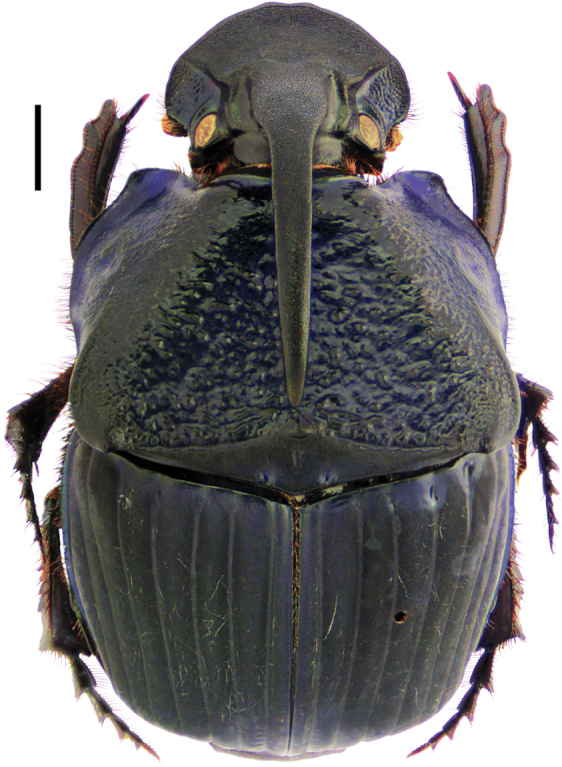
Habitus *Phanaeus lempira* sp. nov. Paratype male (♂), Parque Nacional de Montaña Celaque (14,484624°N, -88,66943°W), Departamento de Lempira, Honduras. Scale bar: 2 mm.

####### Type material.

***Holotype*: male. Honduras.** • **Departamento de Lempira.** Municipio de Gracias. Parque Nacional de Montaña Celaque. Bosque de Pino-Encino. 14,4857°N, -88,6701°W. JD Rivera, RP Salomão, Michel M *leg.* 17.VI.23, 2060 m. ***Allotype*: female. Honduras.** • **Departamento de Lempira.** Municipio de Gracias. Parque Nacional de Montaña Celaque. Bosque de Pino-Encino. 14,4841°N, -88,6688°W. JD Rivera and RP Salomão *leg.* 17.VI.23, 2010 m. ***Paratypes*: Honduras.** • **Departamento de Lempira.** Municipio de Gracias. Parque Nacional de Montaña Celaque. Bosque de Pino-Encino. JD Rivera and RP Salomão, Michel M *leg.*14,5651°N, -88,6329°W. 17.VI.23, 1728 m (1 male); • 14,4928°N, -88,6561°W. 17.VI.23 1721 m (1 male); • 14,4911°N, -88,6569°W. 17.VI.23 1696 m (2 males); • 14,4918°N, -88,6566°W. 17.VI.23 1705 m (1 male); • 14,4922°N, -88,6564°W. 17.VI.23 1704 m (1 male); • 14,4846°N, -88,6694°W. 17.VI.23, 2023 m (2 males); • 14,4849°N, -88,6699°W. 17.VI.23 2037 m (1 male); • 14,5651°N, -88,6323°W. 16.VI.23 1258 m (1 male); • 14,4841°N, -88,6689°W. 17.VI.23 2010 m (2 females). • **Departamento de Francisco Morazán.** Municipio de Tatumbla. El Aguacate, 13°59'N, 87°06'W, 19/7/1991, leg L. Vásquez. EAPZ007.359, (1 male). • **Departamento de Copán.** Municipio de San Pedro. Capucas, 25.11.2018. Leg. D. Díaz, J. Orozco, R. Ortega, (1 male, 1 female).

**Figure 7.**
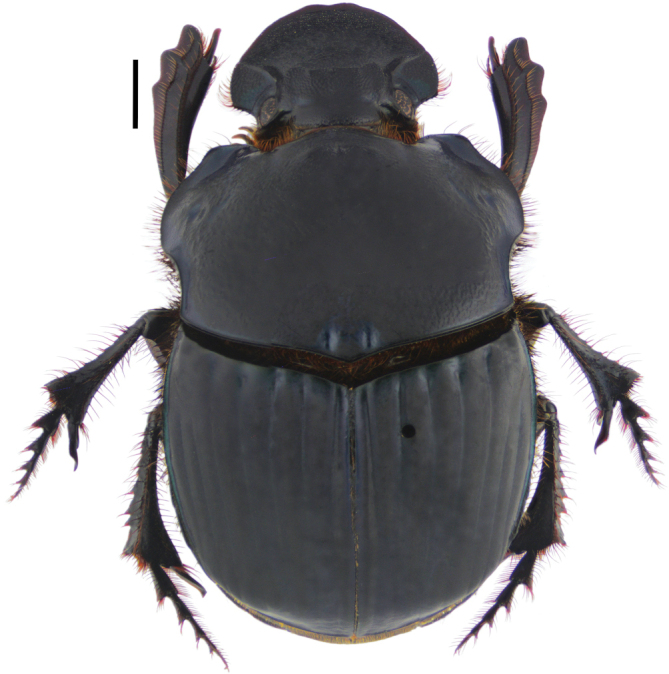
Habitus *Phanaeus lempira* sp. nov. Paratype female, Parque Nacional de Montaña Celaque (14,484624°N, -88,66943°W), Departamento de Lempira, Honduras. Scale bar: 2 mm.

Holotype, allotype, and two male and one female paratypes from Departamento Francisco Morazán and Copán deposited in: Insect Collection, Escuela Agrícola Panamericana El Zamorano, Honduras. One male and one female paratypes from Departamento de Lempira (17-VI-2023, 2024 and 2010 m a.s.l.) are deposited in the National Museum of Costa Rica, San Jose, Costa Rica. Nine male and one female paratypes from Departamento de Lempira were deposited in the Insect Collection of the Hydrobiology Laboratory, School of Biology, Universidad Nacional Autónoma de Honduras.

**Figure 8.**
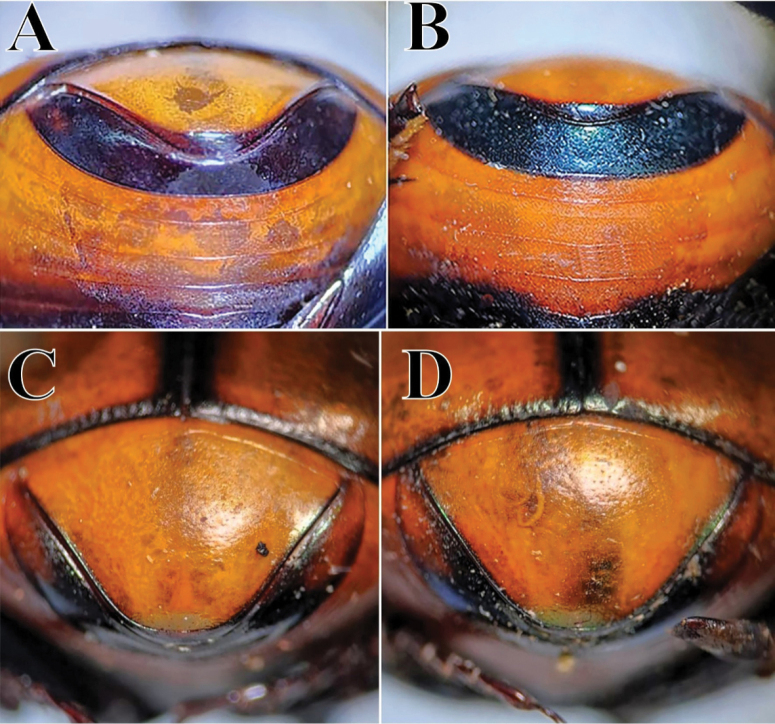
A, B. Last abdominal ventrite of *C. sallei*. A. Male; B. Female, which are always black with yellow borders. C, D. Pygidium of *Canthon sallei*. C. Male; D. Female.

####### Diagnosis.

Large species, dull with dark blue pronotum. Male pronotum covered with vermiculate sculpturing on disk. Male with pronotal anterior carina an inverted V, very strongly developed and raised in the middle. Male with base of pronotal disk with a raised, smooth transverse carina, slightly triangularly arched at the middle, with a well-developed tooth and a mid-sulcus at its base (Fig. 6). Basal fossae clearly impressed. Elytral striae fine, well-defined; interstriae slightly convex and shiny, with coppery-red reflections. Female with metabasitarsus widened apically; outer ridge of ventral surface distinctly more sinuous than inner ridge (Fig. 12).

**Figure 9.**
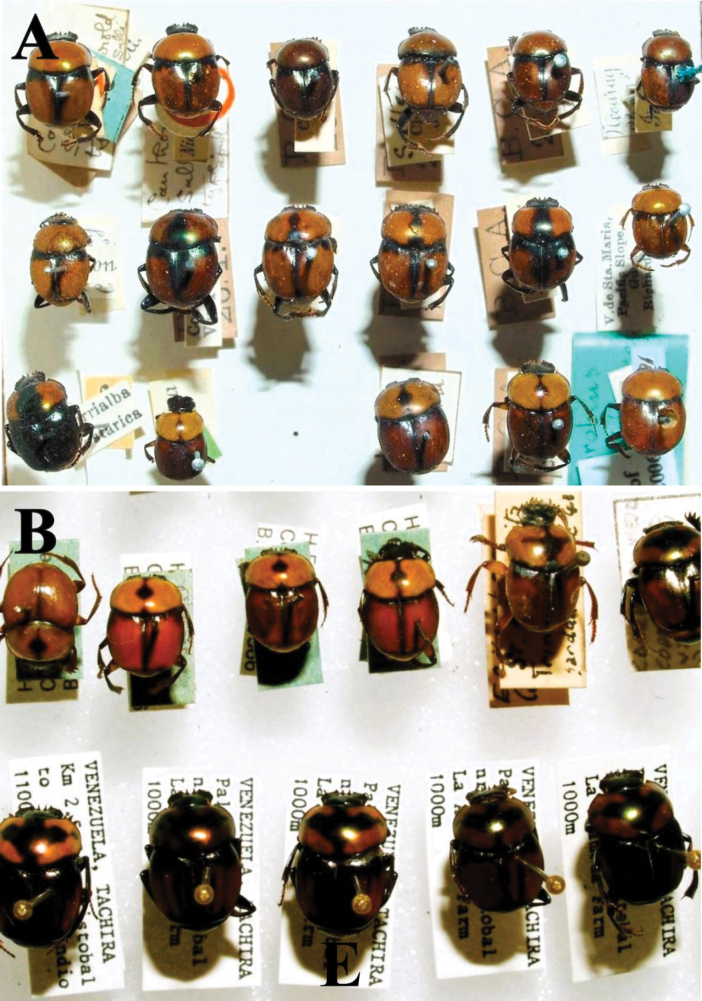
The insect collection of the Natural History Museum, London (photos taken by ÁS in 2009). A. The specimens are *Canthon sallei* from Central America, save one misplaced *C. moniliatus*, third line, second from left on the right of the specimen with the Turrialba, Costa Rica label. The first two specimens on the first row, left, having a round red label, are the syntypes of *C. sallei*. The macular pronotal pattern of the type material exists in Honduras, Nicaragua, and Costa Rica, albeit other macular variations exist for this species as well; B. The four upper left specimens are *Canthon sallei* from Panama, all other seven specimens are *C. triangulatus*. The difference in the pronotal maculae are illustrative.

**Figure 10.**
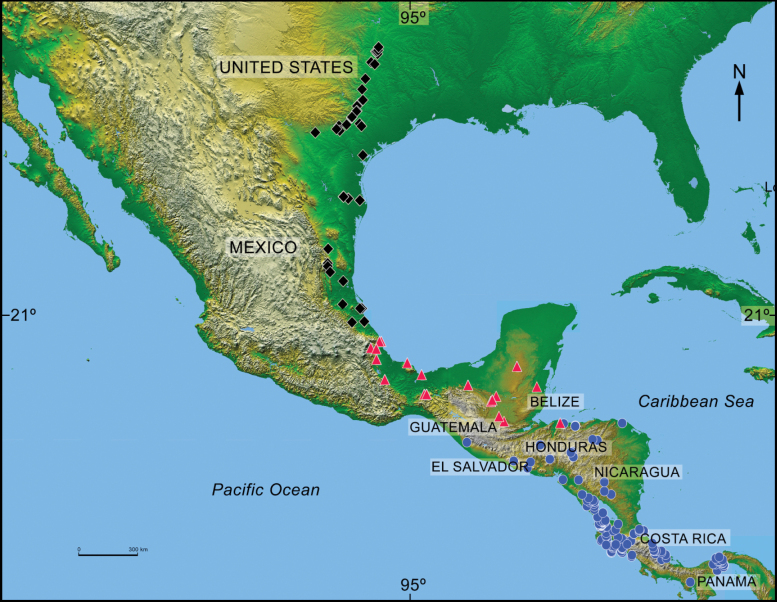
Map of the known distribution of *Canthon cyanellus* (black rhombus), *C. spinosus* (red triangle), and *C. sallei* (blue dot). *Canthon cyanellus* has no subspecies.

**Figure 11.**
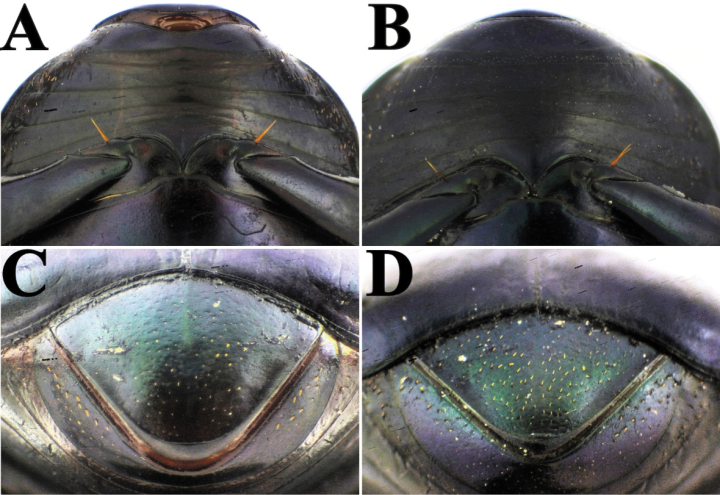
Last abdominal sternite of *Canthon spinosus* A. Male; B. Female, which are somber and unicolorous. Pygidium of *C. spinosus* showing setae C. Male; D. Female.

**Figure 12.**
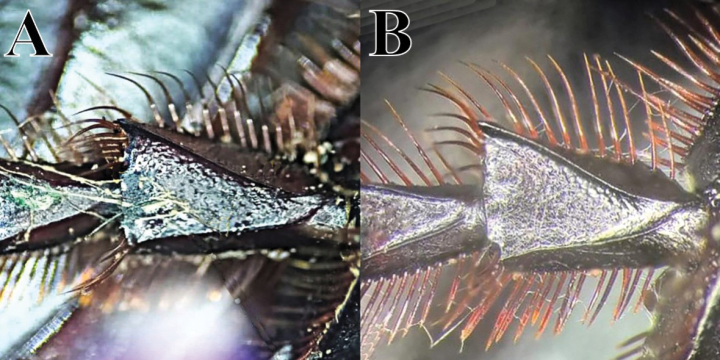
A. Photo of the female metabasitarsus of *Phanaeus melampus*. Texas A&M University Insect Collection, ex F. Psota collection, ex Field Museum, ex David Edmonds collection. Photo courtesy of Mario Cupello, no locality label. B. Photo of the female metabasitarsus of *Phanaeus lempira* sp. nov. Honduras. Parque Nacional de Montaña Celaque. 14,484138°N, -88,668799°W.

####### Description of holotype

**(male, Fig. 6).** Length 22.1 mm. Width 14.9 mm. Dorsum somber, pronotum and pygidium dark blue, elytra appearing black with interstriae having a red-coppery reflection, underside black. Head horn distinctly swollen basally. Pronotum appearing varnished; covered with black-blue vermiculate sculpturing on the disk; anterior carina an inverted V, very strongly developed and raised in the middle; basal medial portion smooth and raised, forming an extended soft triangular arch, acutely denticulate in its anterior center with a clear medial sulcus at its base, basal fossae present. Elytra with fine striae, with obsolete punctures; interstriae finely convex, appearing almost flat, alutaceous, shiny with coppery-red reflections. Profemora coarsely punctate, mesofemora coarsely punctate at apex, metafemora with a few coarse punctures at apex. Pygidium convex, with many coarse punctures and a faint midline.

####### Allotype.

**Female. Fig. 7**. Length 22.1 mm. Width 14.1 mm. Dorsum somber, pronotum with coppery-red reflections on disk and turquoise blue on borders, interstriae with coppery-red reflections and pygidium turquoise blue. Head with very rugose surface; tri-tuberculate cephalic carina with nearly aligned, similar-sized, conical tubercles. Pronotal surface rugose, matte; transverse carina forming an open arch with three conical lobes closely set with a posterior concavity; median lobe higher and more projected than laterals, a small midline carina runs from the median lobe base to the concavity; with two basal impressions. Metabasitarsus widened apically; outer ridge of ventral surface distinctly more sinuous than inner ridge (Fig. 12).

####### Variation.

Males. Length 20.2–23.6 mm. Width 11.9–14.9 mm. Females. Length 20.6–22.1 mm. Width 12.4–14.1 mm.

####### Etymology.

The name is dedicated in honor of the Honduran national hero and symbol of liberty and cultural resistance, who was a captain of the Lenca tribes that fought the Spanish invasion and conquest.

####### Habitat.

This species is distributed between 1528 and 2060 m a.s.l. Pine and pine-oak association are distributed in this park between 1000 and 1500 m a.s.l. on the Atlantic slope, and between 1800 and 2300 m a.s.l. on the Pacific slope (Mejía Ordóñez and House 2002). Six of the seven pine species of the country occur here, *Pinus oocarpa* Schiede ex Schltdl., 1838 being the most common one. Mixed forest (pine, *Liquidambar styraciflua* L., 1753, *Clethra* sp., *Nectandra* sp.) is recorded higher than 1500 m a.s.l. and between 1800 and 2000 m a.s.l. the cloud forest begins on the Atlantic slope of the park, while on the Pacific slope it starts at ca 2300 m a.s.l. The cloud forest is characterized by a significant plant species richness (*Abies guatemalensis* Rehder, 1939, *Alnus* sp., *Pinus pseudostrobus* Lindl., 1839, *Prunus* sp., *Quercus lancifolia* Schltdl. & Cham, 1830, *Taxus globosa* Schltdl., 1838), where tree ferns are very common. It is the highest mountain range in Honduras. This park is home to several endemic species of plants, amphibians, shrews, a scarab gold beetle (*Chrysina pastori* Curoe, 1994), and a scarab flower beetle (*Archedinus antoshkai* Seidel and Arriaga-Varela, 2018).

####### Distribution.

**Figure 13.**
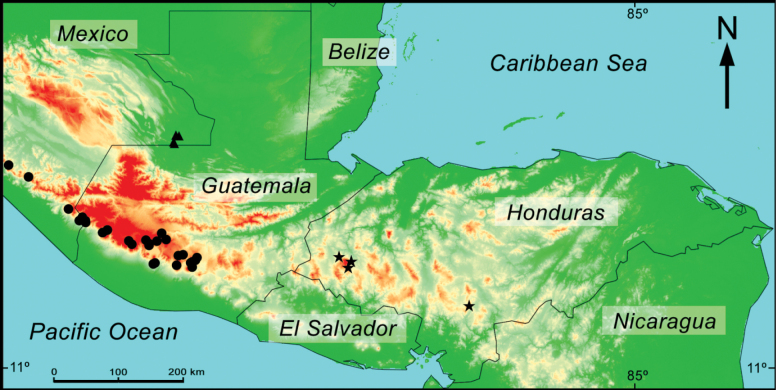
Map of the distribution of *Phanaeus lempira* sp. nov. (stars) and *Ph. guatemalensis* (dots). Triangles represent specimens that were considered initially to belong to *Ph. melampus* by Navarrete-Gutiérrez and Edmonds (2006), but that on photographic inspection show the characteristics of *Ph. lempira*.

*Phanaeus lempira* is closely related to the Mexican endemic *Ph. melampus* Harold, 1863. Edmonds (1994) recorded the latter from three localities in Veracruz and two in Chiapas, and another Chiapas locality was added by Navarrete-Gutiérrez and Edmonds (2006). The nine Navarrete-Gutiérrez and Edmonds specimens were collected at 145 m a.s.l. during February, August, and October in traps baited with human feces in the Montes Azules Biosphere Reserve in tall and medium evergreen tropical forests. Upon request, Navarrete-Gutiérrez provided us with photos of these specimens. Upon close examination, these individuals showed all the characteristics of the new *Phanaeus* species described in this paper. Even though we did not see Edmonds’s (1994) Chiapas specimens, they also come from places covered in wet tropical evergreen forest, El Bosque and Tenejapa, at 1460 m a.s.l., indicating they also most probably belong to this new species. Finally, among the material photographed by Mario Cupello from the Texas A&M University collection, there is one specimen with the characteristics of *Ph. lempira* that bears a label saying only “Tabasco, Messico”. This specimen might have been collected around Teapa, where Tabasco reaches the piedmont of the Chiapas mountains. All this suggests that *Ph. lempira* is distributed from Chiapas to Honduras (Fig. 13), whereas *Ph. melampus* is distributed yonder the Isthmus of Tehuantepec divide – currently known exclusively from Veracruz – and not south of it.

Lizardo et al. (2022: suppl. fig. 1), on predicted and recorded distributions of *Phanaeus* presents a map of the expected distribution of the *Phanaeus amethystinus* species group of which this species is a member. These climatically predicted distributions are somewhat at odds with the actual recorded distributions. For example, their model predicts a possible distribution of *Ph. melampus* (probable sister species of *Ph. lempira*) in the marshy tropical savannah of northeastern Honduras, where no species of *Phanaeus* has been registered at present and where the majority of the collected species belong to the roller guild and some *Onthophagus* taxa as well. These climatic models usually make distribution predictions that are way off the mark in tropical mountainous areas. As Kohlmann et al. (2007) have already discussed, finer-scale systems, like the Life Zones of Holdridge (1967), are better suited for these tropical areas.

####### Phenology.

This species was collected in June, July, and November using traps baited with human feces.

####### Taxonomic remarks.

This species is closely related to *Ph. melampus*, reported from a mountain wet tropical evergreen forest from Veracruz in Mexico collected at 1500 m a.s.l., and to *Ph. guatemalensis* Harold, 1871, reported from forests and adjacent open habitats in the Guatemalan Highlands, distributed from 1500 to 2400 m (Edmonds 1994). The nearest recorded species group specimen to the type locality of this new species is *Ph. guatemalensis*, 230 km away, suggesting a great geographical separation.

**Figure 14.**
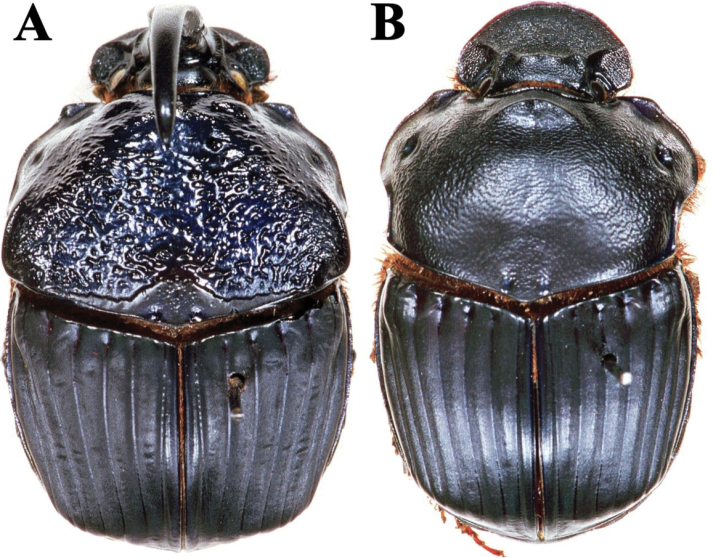
Habitus *Phanaeus melampus* A. Male; B. Female. Veracruz, Orizaba, Mexico. Institut royale des Sciences naturelles de Belgique, through Roger Damoiseau. Photos courtesy of W. D. Edmonds.

The new species *Ph. lempira* can be distinguished from *Ph. melampus* by the shape of the smooth, transverse basal area of the male pronotum, which features a sulcus at the mid-base of the pronotum. In *Ph. lempira*, this area is slightly triangularly arched (Fig. 6), resembling the shape observed in *Ph. amethystinus*. In contrast, *Ph. melampus* exhibits a strongly triangular, punctate area that lacks the sulcus at the base of the medial tooth (Fig. 14A). Additionally, the interstriae of *Ph. melampus* are dull and rarely show a bluish cast, whereas *Ph. lempira* has shiny interstriae with coppery-red reflections. Female specimens of *Ph. melampus* have a semicircular pronotal transverse carina, while *Ph. lempira* features a more open transverse carina (Figs 7, 14B). The female of *Ph. lempira* has a metabasitarsus widened apically, and the outer ridge of the ventral surface is distinctly more sinuous than the inner ridge, similar to *Ph. amethystinus* (Fig. 12B). Conversely, the female of *Ph. melampus* has a narrower metabasitarsus with both ventral ridges running more or less straight (Fig. 12A).

*Phanaeus lempira* can be separated from *Ph. guatemalensis* by the interstriae, flat or weakly convex in *Ph. lempira*, raised mid-longitudinally, appearing costate in *Ph. guatemalensis*. The pronotoum of *Ph. lempira* females have asperate punctures posteromedially, whereas in *Ph. guatemalensis* the area is coarsely punctate to rugo-punctate, appearing roughened.

With this new species, the number of taxa in the *amethystinus* species group (*amethystinus*, *blackalleri*, *guatemalensis*, *melampus*) increases to five. In the work of Lizardo et al. (2022)*Phanaeus genieri*, a member of the *amethystinus* species group, is considered in their distribution prediction analysis, although this species has not yet been reinstated as a valid species. Gillett and Toussaint (2020), based on molecular genetic analysis, considered that this species group appeared during the Miocene around 17 Ma. However, modern species formation seems to have been relatively recent during the Pleistocene, ~ 1 Ma.

### ﻿Key to species of the *amethystinus* group (modified from Edmonds and Zídek 2012)

**Table:** 

1	Punctures of posteromedial portion of female pronotum simple to weakly asperate, weakest in front of basal fossae, area appearing smooth to unaided eye. Elytra dark, somber; metallic color, if any, restricted to margins; pronotum usually dark except for metallic blue lateral margins	**2**
–	Posteromedial portion of female pronotum coarsely punctate to rugo-punctate, area appearing roughened to unaided eye. Elytra in most specimens with metallic color matching that of pronotum and pygidium	**4**
2(1)	Posteromedial portion of female pronotum with simple punctures (×10). Female pronotum bearing a weakly tri-tuberculate, straight transverse ridge whose length clearly exceeds interocular distance; ridge not followed by a distinct concavity. Head horn of large male tapering gradually from base to apex, base not distinctly swollen. Elytral interstriae distinctly convex, weakly shiny. Smooth, transverse, triangular area along posterior margin of male pronotum lacking a tubercle on medial angle. Smaller, length 14–20 mm, usually < 20 mm. Pacific slope of Sierra Madre del Sur in Guerrero and Oaxaca	**Phanaeus (P.) blackalleri Delgado-Castillo**
–	Posteromedial portion of female pronotum with finely asperate punctures (×10). Head horn of large male distinctly swollen basally. Elytral interstriae flat or only weakly convex, dull. Transverse, triangular area along posterior margin of male pronotum with an acute tubercle on medial angle. Larger, length 18–28 mm, usually well over 20 mm	**3**
3(2)	Smooth, slightly triangularly arched basal area of male pronotum features a sulcus at base of medial tooth (Fig. 6), resembling the shape observed in *Ph. amethystinus*. Interstriae with coppery-red reflections. Female specimens feature an open transverse carina (Fig. 7). Female of with metabasitarsus widened apically, outer ridge of ventral surface distinctly more sinuous than inner ridge, similar to *Ph. amethystinus* (Fig. 12B)	**Phanaeus (P.) lempira sp. nov.**
–	Punctate area of highly triangular basal area of male pronotum lacking sulcus at base of medial tooth (Fig. 14A). Interstriae dull and rarely showing bluish cast. Female specimens have a semicircular pronotal transverse carina (Fig. 14B). Female with narrower metabasitarsus with both ventral ridges running more or less straight (Fig. 12A)	**Phanaeus (P.) melampus Harold**
4(1)	At least interstriae 2 and 3, but in most specimens 2–5, highly shiny and raised mid-longitudinally, dull adjacent to striae, appearing costate to unaided eye. Sides of pronotum densely granulate-rugose. Highlands of Guatemala and adjacent Chiapas	**Phanaeus (P.) guatemalensis Harold**
–	All elytral interstriae evenly and in most specimens brightly shiny, evenly convex. Sides of pronotum only moderately densely granulo-rugose. Highlands of central Chiapas, mountains of eastern Oaxaca, Sierra Madre Oriental from Veracruz to southern Tamaulipas	**Phanaeus (P.) amethystinus Harold**

#### *Phanaeus porioni* Arnaud, 2001 (CS, RL, EZ)

Fig. 5B, C

Belize, Honduras.

Arnaud 2001; Edmonds and Zídek 2012; Moctezuma and Halffter 2021a.

**Material examined. Honduras (11): Francisco Morazán**: Distrito Central, Parque Nacional La Tigra; 14°12'42"N, 87°8'51"W, 1464 m; 22.VI.2023; JD Rivera, R Salomão and M Michel leg.; pitfall trap, human dung; UNAH (1). **Lempira**: Gracias, Parque Nacional Montaña de Celaque; 14°33'53"N, 88°37'54"W, 1271 m; 16.VI.2023; JD Rivera, R Salomão and M Michel leg.; pitfall trap, human dung; UNAH (3). **Olancho**: Catacamas, Parque Nacional Sierra de Agalta; 14°54'6"N, 85°52'25"W, 483 m; 3.VII.2023; JD Rivera and R Salomão leg.; pitfall trap, human dung; UNAH (5). Same locality; 14°55'1"N, 85°52'57"W, 586 m; 3.VII.2023; JD Rivera and R Salomão leg.; pitfall trap, human dung; UNAH (2).

#### *Phanaeus wagneri* Harold, 1863 (CS, RL, EZ)

Fig. 5D

Mexico, Guatemala, Honduras, El Salvador, Nicaragua, Costa Rica.

Harold 1863; Bates 1887; Pittier and Biolley 1895; Edmonds 1994; Arnaud 2002a; Edmonds and Zídek 2012; Solís and Kohlmann 2012; Maes et al. 2020; Pablo-Cea et al. 2023.

**Material examined. Honduras (6): Lempira**: Gracias, Parque Nacional Montaña de Celaque; 14°29'5"N, 88°40'11"W, 2030 m; 17.VI.2023; JD Rivera, R Salomão and M Michel leg.; pitfall trap, human dung; UNAH (2). **Yoro**: Olanchito, Refugio Hábitat/Especie Colibrí Esmeralda Hondureño; 15°27'53"N, 86°52'52"W, 412 m; 29.VI.2023; JD Rivera and R Salomão leg.; pitfall trap, human dung; UNAH (4).

#### *Sulcophanaeus chryseicollis* (Harold, 1863) (NR, CS, EZ)

Fig. 5E

*Phanaeus chryseicollis* Harold, 1863.

Mexico, Honduras.

Harold 1863; Bates 1887; Edmonds 2000.

**Material examined. Honduras (9): Olancho**: Catacamas, Parque Nacional Sierra de Agalta; 14°54'6"N, 85°52'25"W, 483 m; 3.VII.2023; JD Rivera and R Salomão leg.; pitfall trap, human dung; UNAH (4). Same locality; 14°55'1"N, 85°52'57"W, 586 m; 3.VII.2023; JD Rivera and R Salomão leg.; pitfall trap, human dung; UNAH (5).

##### ﻿Sisyphini

#### *Sisyphus mexicanus* Harold, 1863 (NR, CS, RL)

Fig. 5F

Mexico, Honduras, El Salvador, Nicaragua, Costa Rica.

Harold 1863; Bates 1887; Haaf 1955; Howden 1966; Halffter 2003; Rivera and Cantarero 2011; Solís and Kohlmann 2012; Capello and Halffter 2019; Maes et al. 2020; Pablo-Cea et al. 2023.

**Material examined. Honduras (535): Yoro**: Olanchito, Refugio Hábitat/Especie Colibrí Esmeralda Hondureño; 15°27'53"N, 86°52'52"W, 412 m; 29.VI.2023; JD Rivera and R Salomão leg.; pitfall trap, human dung; UNAH (232). Same locality; 15°27'9"N, 86°53'41"W, 338 m; 29.VI.2023; JD Rivera and R Salomão leg.; pitfall trap, human dung; UNAH (199). Same locality; 15°25'41"N, 86°53'38"W, 243 m; 30.VI.2023; JD Rivera and R Salomão leg.; pitfall trap, human dung; UNAH (61). Same locality; 15°25'57"N, 86°52'31"W, 255 m; 30.VI.2023; JD Rivera and R Salomão leg.; pitfall trap, human dung; UNAH (43).

**Remarks.** This species has been considered in the past to be a typical inhabitant of tropical rainforests. For instance, Halffter (2003: 44) indicates that it is not an abundant species and that it inhabits evergreen and semideciduous tropical forests and secondary communities in Mexico. However, one of the coauthors (BK) collected it in 1976 in Palma Sola, Veracruz, rolling cow dung balls, interestingly only on paths devoid of vegetation, traversing introduced tropical grass. Recent work confirms the latter observation and suggests the earlier view is incorrect. Alvarado et al. (2018) report a significant number of *S. mexicanus* in their analysis (12,628 specimens of the 169,372 total), this species being the fifth most numerous one after (in decreasing order) Canthon (C.) chiapas (cited as C. (C.) indigaceus chevrolati), *Onthophagus landolti*, *Pseudocanthon perplexus*, and Canthon (Gl.) euryscelis. Most of the specimens were found in dry tropical forest fragments in the north-central region of the Yucatan Peninsula.

The present work arrives at a similar conclusion. *Sisyphus mexicanus* has been caught in significant numbers in Olanchito, an area covered by tropical dry forest. This species is the third most numerous, after *Onthophagus landolti* and Canthon (C.) chiapas. The fourth and fifth most numerous species are Canthon (Gl.) championi and *Onthophagus batesi*. As in the previous example, it is an ecosystem dominated mainly by ball-rolling dung beetles. All this suggests that *Sisyphus mexicanus* is a denizen of the land-locked Central American tropical dry forest. This species has been reported as rare in all other areas (South-Eastern Mexico, El Salvador, Nicaragua, Costa Rica) (Solís and Kohlmann 2012; Maes et al. 2020; Pablo-Cea et al. 2023).

Haaf (1955), in his study of the genus *Sisyphus*, suggested that *S. crispatus* Gory, 1833 is formed by three subspecies, *S. c. crispatus* from South and West Africa, *S. c. hirtus* Wiedemann, 1823 from India and Sri Lanka, and. *S. c. mexicanus* Harold, 1863 from Mexico. Haaf (1955) considered them all members of one species because all three lack any spines or projections on the metafemora and metatibiae in the male specimens. Pablo-Cea et al. (2023) followed this taxonomic arrangement in their catalog of the dung beetle fauna of El Salvador, a taxonomic use which we deem incorrect. Haaf (1955) separated *S. c. crispatus* from *S. c. mexicanus* because the last subspecies have much stronger punctuation and more pointed clypeal teeth than the first ones. Paschalidis (1974), in her M.Sc. thesis, synonymized *S. crispatus* with *S. costatus* (Thunberg, 1818), a South African species living in moist savannas and upland grasslands. However, Paschalidis (1974) made no mention of any of the subspecies, as Haaf (1955) had considered in his study. Subsequently, Daniel et al. (2018) considered *S. crispatus* as a nomen dubium. On the other hand, *S. mexicanus* has been treated as a valid species in all American systematic studies of this taxon (Howden 1965; Halffter 2003). Moreover, Daniel et al. (2018) are not confident in stating which of the two species (*S. caffer* Boheman, 1857, *S. sordidus* Boheman, 1857) might be the possible junior synonym of *S. crispatus*. As Daniel et al. (2018: 21) indicate that the type specimen of *S. crispatus* has been lost and as the original description and image presented are imprecise and insufficient to determine the identity of the species, we then consider here *Sisyphus mexicanus* as a valid taxon at the species level and not as a subspecies of a nomen dubium. Finally, this decision is supported by the photographic comparison of the African and American species (Daniel et al. 2018: fig. 5B, F). *Sisyphus caffer* and *S. sordidus* have denser and longer dorsal setae cover than *S. mexicanus*; both African species have a rounder thorax and completely rounded anterior pronotal humeri, whereas the American species has a less rounded thorax and very pointed anterior pronotal humeri; the African species have dented metatibiae, whereas the American species is truly serrated. These morphological differences make it clear that the African and American species are not conspecific, as Haaf (1955) had suggested, and as such *S. mexicanus* cannot be a subspecies of *S. crispatus*.

##### ﻿Scarabaeinae incertae sedis

#### *Canthidium ardens* Bates, 1887 (CS, RL)

Mexico, Guatemala, Honduras, Nicaragua, Costa Rica, Panama.

Bates 1887; Creedy and Mann 2012; Pittier and Biolley 1895; Howden and Young 1981; Solís and Kohlmann 2004; Kohlmann and Solís 2006; Solís and Kohlmann 2012; Cupello 2018.

**Material examined. Honduras (9): Comayagua**: Siguatepeque, Parque Nacional Cerro Azul Meámbar; 14°52'21"N, 87°54'7"W, 993 m; 16.VII.2023; JD Rivera leg.; pitfall trap, human dung; UNAH (3). **Olancho**: Catacamas, Parque Nacional Sierra de Agalta; 14°55'1"N, 85°52'57"W, 586 m; 3.VII.2023; JD Rivera and R Salomão leg.; pitfall trap, human dung; UNAH (6).

#### *Canthidium centrale* Boucomont, 1928 (CS, RL)

*Canthidium martinezi* Edmonds & Halffter, 1978.

Mexico, Guatemala, Honduras, Nicaragua, Costa Rica, Panama, Colombia, Suriname, French Guiana, Ecuador.

Boucomont 1928; Howden and Young 1981; Medina et al. 2001; Solís and Kohlmann 2004; Kohlmann and Solís 2006; Creedy and Mann 2012; Solís and Kohlmann 2012; Cupello 2018.

**Material examined. Honduras (22): Comayagua**: Siguatepeque, Parque Nacional Cerro Azul Meámbar; 14°53'33"N, 87°51'21"W, 720 m; 15.VII.2023; JD Rivera leg.; pitfall trap, human dung; UNAH (8). Same locality; 14°50'40"N, 87°57'12"W, 748 m; 15.VII.2023; JD Rivera leg.; pitfall trap, human dung; UNAH (3). Same locality; 14°52'50"N, 87°54'59"W, 712 m; 16.VII.2023; JD Rivera leg.; pitfall trap, human dung; UNAH (10). **Yoro**: Olanchito, Refugio Hábitat/Especie Colibrí Esmeralda Hondureño; 15°27'53"N, 86°52'52"W, 412 m; 29.VI.2023; JD Rivera and R Salomão leg.; pitfall trap, human dung; UNAH (1).

#### *Canthidium laetum* Harold, 1867 (CS, RL, EZ)

*Canthidium granivorum* Halffter & Halffter, 1978.

Mexico, Guatemala, Honduras, El Salvador, Nicaragua, Costa Rica.

Harold 1867a; Bates 1887; Solís and Kohlmann 2004; Kohlmann and Solís 2006; Solís and Kohlmann 2012; Cupello 2018; Pablo-Cea et al. 2023.

**Material examined. Honduras (6): Francisco Morazán**: Distrito Central, Universidad Nacional Autónoma de Honduras – Ciudad Universitaria; 14°5'2"N, 87°11'32"W, 990 m; 20.VII.2024; JD Rivera leg.; pitfall trap, human dung; UNAH (5). **Yoro**: Olanchito, Refugio Hábitat/Especie Colibrí Esmeralda Hondureño; 15°27'53"N, 86°52'52"W, 412 m; 29.VI.2023; JD Rivera and R Salomão leg.; pitfall trap, human dung; UNAH (1).

#### *Canthidium moroni* Kohlmann & Solís, 2006 (EZ)

Kohlmann 2003, 2004, included as part of *C. vespertinum*.

Mexico, Guatemala, Honduras.

Kohlmann and Solís 2006; Cupello 2018; Capello and Halffter 2019; Sánchez-Hernández et al. 2022.

#### *Canthidium tuberifrons* Howden & Young, 1981 (NR, EZ)

Honduras, Nicaragua, Costa Rica, Panama.

Howden and Young 1981; Martínez and Halffter 1986; Solís and Kohlmann 2004; 2012, Cupello 2018; Hernández et al. 2020.

#### *Canthidium vespertinum* Howden & Young, 1981 (EZ)

Mexico, Honduras, Costa Rica, Panama.

Howden and Young 1981; Solís and Kohlmann 2004, 2012; Cupello 2018.

#### *Cryptocanthon* sp.* (RL)

Honduras.

Creedy and Mann 2012.

#### *Uroxys boneti* Pereira & Halffter, 1961 (RL)

*Uroxys bidentis* Howden & Young, 1981.

Mexico, Guatemala, Honduras, El Salvador, Nicaragua, Costa Rica, Panama, Colombia, Ecuador.

Pereira and Halffter 1961; Delgado and Kohlmann 2007; Solís and Kohlmann 2012; Solís and Kohlmann 2013.

#### *Uroxys bonplandi* Kohlmann, Solís & Alvarado, 2019 (NR, EZ)

Honduras, Costa Rica.

Kohlmann, Solís and Alvarado 2019.

#### *Uroxys deavilai* Delgado & Kohlmann, 2007 (NR, CS, EZ)

Mexico, Guatemala, Honduras, El Salvador, Nicaragua, Costa Rica.

Delgado and Kohlmann 2007; Solís and Kohlmann 2012, 2013; Pablo-Cea et al. 2023.

**Material examined. Honduras (77): Yoro**: Olanchito, Refugio Hábitat/Especie Colibrí Esmeralda Hondureño; 15°27'53"N, 86°52'52"W, 412 m; 29.VI.2023; JD Rivera and R Salomão leg.; pitfall trap, human dung; UNAH (41). Same locality; 15°27'9"N, 86°53'41"W, 338 m; 29.VI.2023; JD Rivera and R Salomão leg.; pitfall trap, human dung; UNAH (36).

#### *Uroxys microcularis* Howden & Young, 1981 (NR, CS, EZ)

Mexico, Guatemala, Honduras, El Salvador, Nicaragua, Costa Rica, Panama.

Howden and Young 1981; Delgado and Kohlmann 2007; Solís and Kohlmann 2012; 2013, Pablo-Cea et al. 2023.

**Material examined. Honduras (65): Atlántida**: Tela, Parque Nacional Jeannette Kawas; 15°52'0"N, 87°37'0"W, 100 m; 11.X.2024; JD Rivera leg.; pitfall trap, human dung; UNAH (15). **Comayagua**: Siguatepeque, Parque Nacional Cerro Azul Meámbar; 14°50'40"N, 87°57'12"W, 748 m; 15.VII.2023; JD Rivera leg.; pitfall trap, human dung; UNAH (3). Same locality; 14°52'21"N, 87°54'7"W, 993 m; 16.VII.2023; JD Rivera leg.; pitfall trap, human dung; UNAH (24). Same locality; 14°52'50"N, 87°54'59"W, 712 m; 16.VII.2023; JD Rivera leg.; pitfall trap, human dung; UNAH (4). **Francisco Morazán**: Distrito Central, Parque Nacional La Tigra; 14°11'38"N, 87°8'34"W, 1439 m; 22.VI.2023; JD Rivera, R Salomão and M Michel leg.; pitfall trap, human dung; UNAH (2). Same locality; 14°12'42"N, 87°8'51"W, 1464 m; 22.VI.2023; JD Rivera, R Salomão and M Michel leg.; pitfall trap, human dung; UNAH (2). Distrito Central, Universidad Nacional Autónoma de Honduras – Ciudad Universitaria; 14°5'2"N, 87°11'32"W; 20.VII.2024; 990 m; JD Rivera leg.; pitfall trap, human dung; UNAH (7). **Olancho**: Catacamas, Parque Nacional Sierra de Agalta; 14°54'6"N, 85°52'25"W, 483 m; 3.VII.2023; JD Rivera and R Salomão leg.; pitfall trap, human dung; UNAH (6). Same locality; 14°55'1"N, 85°52'57"W, 586 m; 3.VII.2023; JD Rivera and R Salomão leg.; pitfall trap, human dung; UNAH (2).

#### *Uroxys micros* Bates, 1887 (CS, EZ)

Mexico, Guatemala, Honduras, Nicaragua, Costa Rica, Panama, Colombia.

Bates 1887; Howden and Young 1981; Delgado and Kohlmann 2007; Solís and Kohlmann 2012, 2013.

**Material examined. Honduras (54): Comayagua**: Siguatepeque, Parque Nacional Cerro Azul Meámbar; 14°50'40"N, 87°57'12"W, 748 m; 15.VII.2023; JD Rivera leg.; pitfall trap, human dung; UNAH (3). Same locality; 14°52'21"N, 87°54'7"W, 993 m; 16.VII.2023; JD Rivera leg.; pitfall trap, human dung; UNAH (21). Same locality; 14°52'50"N, 87°54'59"W, 712 m; 16.VII.2023; JD Rivera leg.; pitfall trap, human dung; UNAH (6). **Francisco Morazán**: Distrito Central, Parque Nacional La Tigra; 14°11'38"N, 87°8'34"W, 1439 m; 22.VI.2023; JD Rivera, R Salomão and M Michel leg.; pitfall trap, human dung; UNAH (6). **Lempira**: Gracias, Parque Nacional Montaña de Celaque; 14°33'53"N, 88°37'54"W, 1271 m; 16.VI.2023; JD Rivera, R Salomão and M Michel leg.; pitfall trap, human dung; UNAH (4). Same locality; 14°29'5"N, 88°40'11"W, 2030 m; 17.VI.2023; JD Rivera, R Salomão and M Michel leg.; pitfall trap, human dung; UNAH (1). **Olancho**: Catacamas, Parque Nacional Sierra de Agalta; 14°55'1"N, 85°52'57"W, 586 m; 3.VII.2023; JD Rivera and R Salomão leg.; pitfall trap, human dung; UNAH (1). Catacamas, Aldea la Concepción; 14°45'57"N, 85°48'6"W, 320 m; 4.VII.2023; JD Rivera and R Salomão leg.; pitfall trap, human dung; UNAH (2). **Yoro**: Olanchito, Refugio Hábitat/Especie Colibrí Esmeralda Hondureño; 15°27'53"N, 86°52'52"W, 412 m; 29.VI.2023; JD Rivera and R Salomão leg.; pitfall trap, human dung; UNAH (10).

#### *Uroxys nebulinus* Howden & Gill, 1987 (NR, CS)

Honduras, Costa Rica, Panama.

Howden and Gill 1987; Solís and Kohlmann 2012, 2013.

**Material examined. Honduras (4): Comayagua**: Siguatepeque, Parque Nacional Cerro Azul Meámbar; 14°52'21"N, 87°54'7"W, 993 m; 16.VII.2023; JD Rivera leg.; pitfall trap, human dung; UNAH (4).

## ﻿Discussion

Geographical gaps in species number assessments limit and bias the comprehension of large-scale taxonomic and ecological patterns (Meyer et al. 2015). In Central American countries, many biological groups have been scarcely studied, including dung beetles (Howden and Young 1981; Solís and Kohlmann 2012; Maes et al. 2020; Pablo-Cea et al. 2023). Cupello et al. (2023) in their study clearly stated that Honduras was one of the few New World countries that still lacked an inventory of its Scarabaeinae fauna. This is the first large-scale work encompassing the dung beetle diversity in Honduras. New species are registered, and a representative portion of the country is sampled. This is a first step in understanding the magnitude of dung beetle biodiversity in Honduras.

### ﻿Biodiversity

A total of 25 genera and 98 species of dung beetles were recorded for Honduras. Of this total, four species are endemic to the country, four are new species awaiting description, and two have been introduced, *Digitonthophagus gazella* and *Euoniticellus intermedius*. The latest count of Honduran species recorded only 42 species for the country (see Cupello et al. 2023). Therefore, this study more than doubles the previous count. If we compare the species numbers and species density with those of Nicaragua (Maes et al. 2020), we can see that both countries reveal similar values. However, compared to Panama or El Salvador (Howden and Young 1981; Pablo-Cea et al. 2023), the low dung beetle species richness in Honduras mainly suggests that more collecting is needed, as is likely the case with Nicaragua (Table 1). According to the species distribution maps (refer to “Distribution atlas of the dung beetles of Honduras” section), significant information gaps persist in the southern, western, and eastern regions of the country. Future research should aim to address these gaps by focusing on hard-to-access areas such as the Honduran Mosquitia in the east; underexplored cloud forests within the Western Mountain Range and the Montecillos Mountain Range in the west-central-southern region; as well as in the Honduran dry corridor, which spans the Pacific slope and extends from the central-western region to the southernmost parts of the country, to name a few examples. More sampling throughout Honduras will permit a fairer comparison with other countries in the region that have significantly greater research efforts, while also providing a clearer understanding of the true biodiversity potential of Honduras.

**Table 1.** Number of known Scarabaeinae species per genera in Honduras, El Salvador, Nicaragua, Costa Rica, and Panama. *Introduced species. *Copris boucardi* is being cited from El Salvador. This citation is probably a misidentification because this species lives in dry pine-oak forests in Mexico’s Sierra Madre del Sur (Guerrero and Oaxaca) (Delgado and Kohlmann 2001). Table derived from Ratcliffe (2002), Maes et al. (2020), Pablo-Cea et al. (2023), and Hernández et al. (2024a, b).

Genus	Honduras 112,492 km^2^	El Salvador 21,040 km^2^	Nicaragua 130,373 km^2^	Costa Rica 51,100 km^2^	Panama 75,517 km^2^
* Agamopus *	0	1	1	1	1
* Anomiopus *	0	0	0	1	1
* Ateuchus *	6	2	4	11	5
* Bdelyrus *	0	0	0	1	2
* Bradypodidium *	0	0	0	1	1
* Canthidium *	6	2	9	25	19
* Canthon *	10	7	11	19	19
* Copris *	9	6	5	7	4
* Coprophanaeus *	4	2	3	7	6
* Cryptocanthon *	1	0	0	4	4
* Deltochilum *	5	4	6	8	7
* Dichotomius *	5	3	4	11	9
Digitonthophagus*	1	1	1	0	1
Euoniticellus*	1	1	1	1	1
* Eurysternus *	6	1	5	9	8
* Eutrichillum *	0	0	0	1	0
* Malagoniella *	1	1	1	1	0
* Megathoposoma *	1	0	1	1	1
* Onoreidium *	0	0	0	1	1
* Ontherus *	0	0	2	4	3
* Onthophagus *	21	10	19	40	24
* Oxysternon *	0	0	0	1	2
* Phanaeus *	6	4	5	8	4
* Pseudocanthon *	1	1	1	1	1
* Scatimus *	1	1	1	2	2
* Scybalocanthon *	1	0	1	1	1
* Sisyphus *	1	1	1	1	0
* Sulcophanaeus *	1	0	1	2	2
* Sylvicanthon *	1	0	1	1	1
* Trichillidium *	0	0	1	1	2
* Uroxys *	6	3	4	13	12
Total	95	51	88	185	141
Species density per 100 km^2^	0.0845	0.2424	0.0675	0.3627	0.1816

Notably, the majority of Honduran endemic species are found within the cloud forests. These species might also be present in similar forests in neighboring Belize, Guatemala, and Nicaragua because these countries have also been poorly sampled. The endemic species of the genera *Ateuchus*, *Copris*, *Haplogeotrupes*, *Phanaeus*, and *Onthophagus* are associated with this pattern, as the present study indicates. A similar phenomenon occurs in the mountain cloud forests of Mexico and Costa Rica (Kohlmann et al. 2019, 2023), where the greatest dung beetle diversity and endemicity are concentrated in their cloud forests. Such ecosystems are considered ancient forests that have an oligocenic-miocenic origin (Rzedowski 1996).

Regarding Bolboceratidae, the tribe Athyreini is of Neotropical origin, whereas the tribe Ceratotrupini, within the family Geotrupidae, is of Nearctic origin. The low numbers of Athyreini in Honduras suggest that the country has not yet been well-collected (Table 2). On the other hand, the Ceratotrupini show a gradual reduction of north-south diversity, as expected for a Nearctic group (Table 2). Honduras represents the southernmost distribution for this group. It is important to consider that most Bolboceratidae and Geotrupidae of Honduras were sampled with pitfall traps baited with excrement. Previous literature highlights the importance of other sampling methods that can enhance the biodiversity assessment of Bolboceratidae and Geotrupidae beetles, such as malaise traps, light traps, pitfall traps baited with fungi, interception traps, soil samples, and active collection (Ćurčić et al. 2019; Iannuzzi et al. 2021). In this still incipient scenario of Bolboceratidae and Geotrupidae biodiversity assessment in Honduras, it is of noteworthy importance to consider and include different sampling techniques besides pitfall traps.

**Table 2:** The number of species and species density of Athyreini and Ceratotrupini in Oaxaca, Honduras, and Panama (Ratcliffe 2002; Kohlmann et al. 2023).

	Athyreini species number	Ceratotrupini species number	Athyreini density per 100 km^2^	Ceratotrupini density per 100 km^2^
Oaxaca 93,757 km2	5	7	0.0053	0.0075
Honduras 112,492 km2	1	1	0.0009	0.0009
Panama 75,517 km2	7	0	0.0093	0.0000

The low *Canthidium* numbers of Honduras suggest again that the country has not been adequately sampled or has low biodiversity for this genus (Table 3). The *Copris* numbers, in turn, reflect the expected north-to-south biodiversity decrease of a genus of northern origin (Table 3).

**Table 3:** The number of species and species density of *Canthidium* and *Copris* in Oaxaca, Honduras, and Panama (Ratcliffe 2002; Kohlmann et al. 2023).

	*Canthidium* species number	*Copris* species number	*Canthidium* density per 100 km^2^	*Copris* density per 100 km^2^
Oaxaca 93,757 km2	12	12	0.0128	0.0128
Honduras 112,492 km2	6	9	0.0053	0.0080
Panama 75,517 km2	19	4	0.0252	0.0053

### ﻿Doubtful, misidentified, and unconfirmed species

Creedy and Mann (2012) reported an *Ateuchus chrysopyge* in the montane moist forests of Cusuco National Park. Subsequently, Cupello (2022) found that it is a new species, which is in the process of being described. Several specimens from the Cusuco National Park were found in the insect collection of the Escuela Agrícola Panamericana Zamorano and, upon closer inspection, were found to belong to the new *Ateuchus* species discovered by Cupello (2022).

As suggested in Table 1, some dung beetle genera are most probably present in Honduras: *Agamopus*, *Bdelyrus*, and *Ontherus*. Those genera have specific life-history traits, e.g., some species live in *Atta* nests or inside bromeliads and feed on debris (see Huijbregts 1984; Gill 1991) that make it difficult to collect them by standard techniques, such as the pitfall traps. Further collecting with different sampling methods, such as light trap and flight intercept trap, might confirm their presence. *Dichotomius centralis* is most probably present along the dry tropical forest of the Pacific coast, but has not yet been collected.

### ﻿Introduced species

Honduras records two introduced species from Africa, *Digitonthophagus gazella* and *Euoniticellus intermedius*. Both species were introduced into the USA during the 1970s for dung control in Texas and California and have spread southward since (Fincher et al. 1983; Kohlmann 1984). Rivera and Cantarero (2011) cited *D. gazella* for the first time in Honduras. They registered it within cow dung in lowland shrublands and tropical pine forests in Yuscarán, El Paraíso. *Digitonthophagus gazella* must have arrived much earlier because Kohlmann (1994) reported it for neighboring Guatemala in 1987. However, the earliest official record is from 1999, housed in the Zamorano Insect Collection, specifically in the department of Colón, located on the northern coast of Honduras. Rivera and Cantarero (2011) indicate that *D. gazella* and *E. intermedius* show a marked preference for open pastures, thus representing bioindicators of open and degraded environments in such landscapes.

The first record for *E. intermedius* is from El Paraíso, Yuscarán, in 2012, reported by Rivera and Schlein (2014). The species was collected in cow dung in pastures and shrubland. This species may have invaded Honduras earlier because the first record for neighboring El Salvador stems from 2003 (Solís and Kohlmann 2012). In addition, *E. intermedius* was captured from cow dung in cattle pastures at Olanchito, Yoro. This capture represents the northernmost recorded distribution of the species within the country. No specimens were found in forest patches, suggesting that the species is unable to invade preserved environments, even when pastures are in close proximity (see González-Tokman et al. 2024).

### ﻿Distribution atlas of the dung beetles of Honduras

Distribution maps are organized alphabetically by genus, followed by species. Distribution data are derived from the 2023 and 2024 collections, the datasets presented in the articles used for this review (see Checklist, taxonomic notes, and remarks section), and the specimen database of the entomological collection of the Escuela Agrícola Panamericana El Zamorano (Figs 15–25).

**Figure 15.**
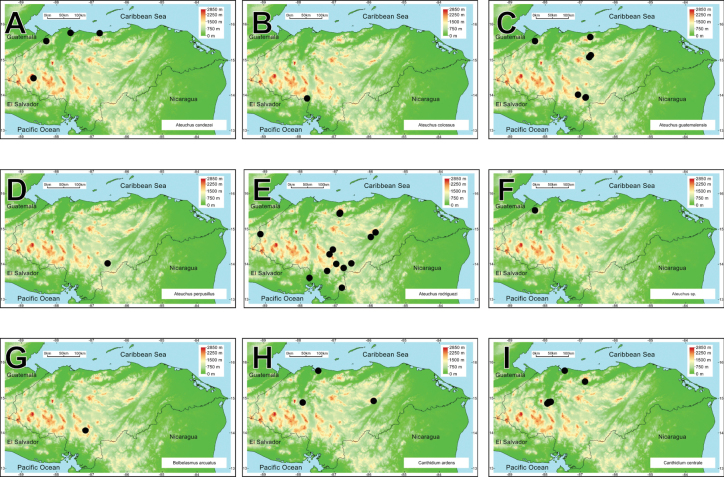
Recorded localities of A. *Ateuchus candezei*; B. *Ateuchus colossus*; C. *Ateuchus guatemalensis*; D. *Ateuchus perpusillus*; E. *Ateuchus rodriguezi*; F. *Ateuchus* sp.; G. *Bolbelasmus arcuatus*; H. *Canthidium ardens*; I. *Canthidium centrale* in Honduras.

**Figure 16.**
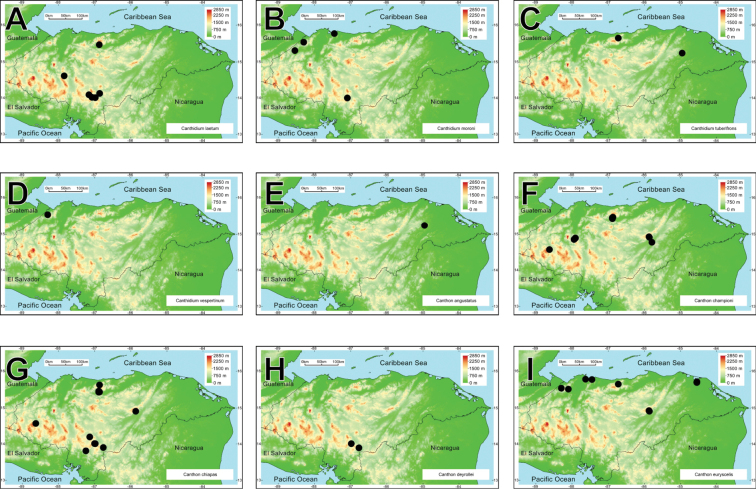
Recorded localities of A. *Canthidium laetum*; B. *Canthidium moroni*; C. *Canthidium tuberifrons*; D. *Canthidium vespertinum*; E. *Canthon angustatus*; F. *Canthon championi*; G. *Canthon chiapas*; H. *Canthon deyrollei*; I. *Canthon euryscelis* in Honduras.

**Figure 17.**
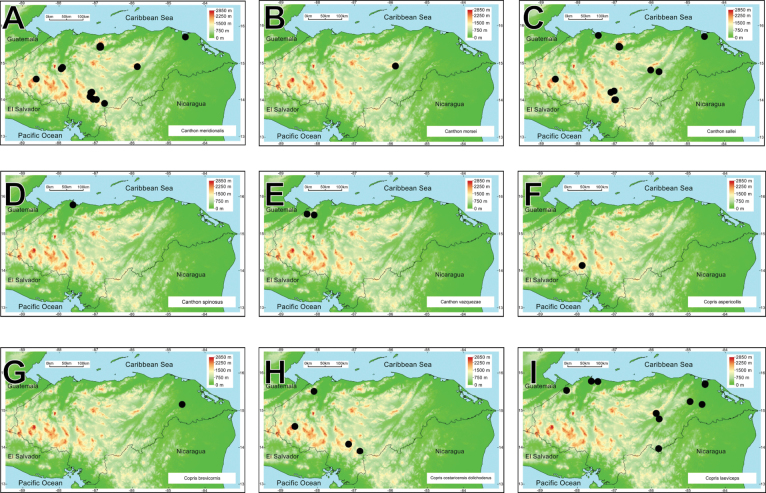
Recorded localities of A. *Canthon meridionalis*; B. *Canthon morsei*; C. *Canthon sallei*; D. *Canthon spinosus*; E. *Canthon vazquezae*; F. *Copris aspericollis*; G. *Copris brevicornis*; H. *Copris costaricensis dolichocerus*; I. *Copris laeviceps* in Honduras.

**Figure 18.**
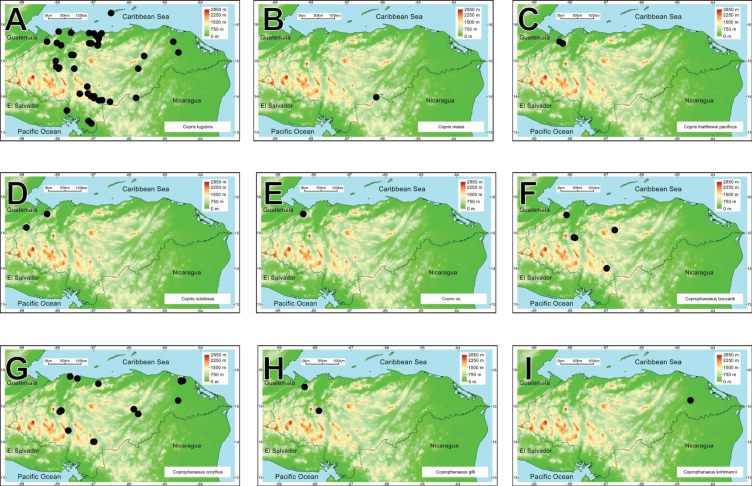
Recorded localities of A. *Copris lugubris*; B. *Copris maesi*; C. *Copris matthewsi pacificus*; D. *Copris nubilosus*; E. *Copris* sp; F. *Coprophanaeus boucardi*; G. *Coprophanaeus corythus*; H. *Coprophanaeus gilli*; I. *Coprophanaeus kohlmanni* in Honduras.

**Figure 19.**
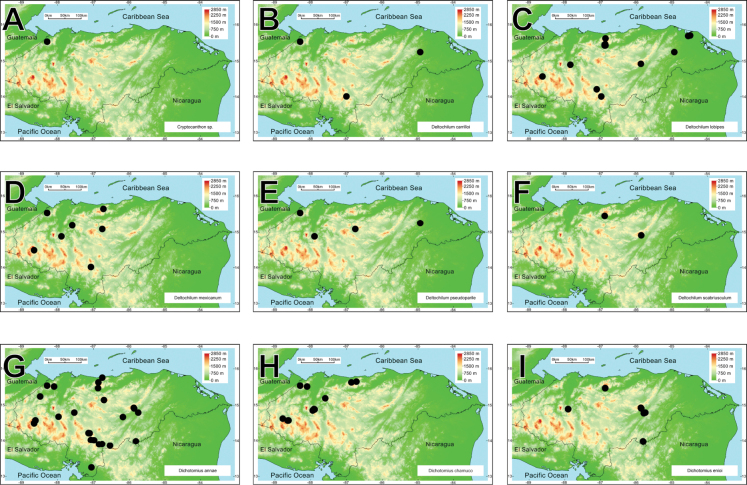
Recorded localities of A. *Cryptocanthon* sp.; B. *Deltochilum carrilloi*; C. *Deltochilum lobipes*; D. *Deltochilum mexicanum*; E. *Deltochilum pseudoparile*; F. *Deltochilum scabriusculum*; G. *Dichotomius annae*; H. *Dichotomius chamuco*; I. *Dichotomius enioi* in Honduras.

**Figure 20.**
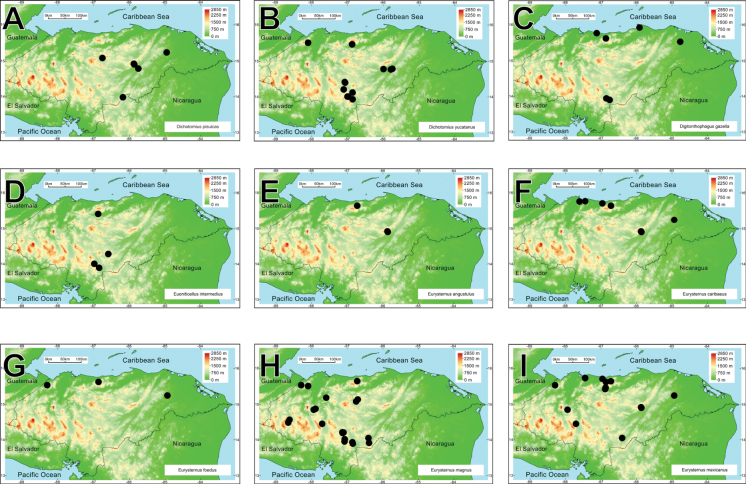
Recorded localities of A. *Dichotomius pisuicas*; B. *Dichotomius yucatanus*; C. *Digitonthophagus gazella*; D. *Euoniticellus intermedius*; E. *Eurysternus angustulus*; F. *Eurysternus caribaeus*; G. *Eurysternus foedus*; H. *Eurysternus magnus*; I. *Eurysternus mexicanus* in Honduras.

**Figure 21.**
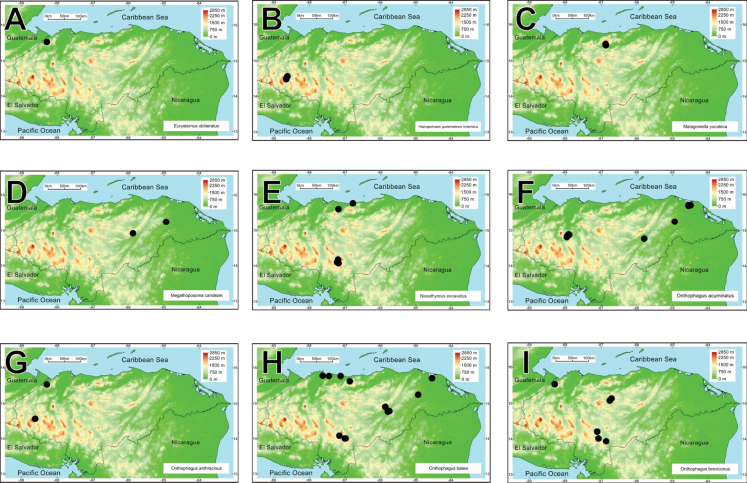
Recorded localities of A. *Eurysternus obliteratus*; B. *Haplogeotrupes guatemalensis tridentatus*; C. *Malagoniella yucateca*; D. *Megathoposoma candezei*; E. *Neoathyreus excavatus*; F. *Onthophagus acuminatus*; G. *Onthophagus anthracinus*; H. *Onthophagus batesi*; I. *Onthophagus breviconus* in Honduras.

**Figure 22.**
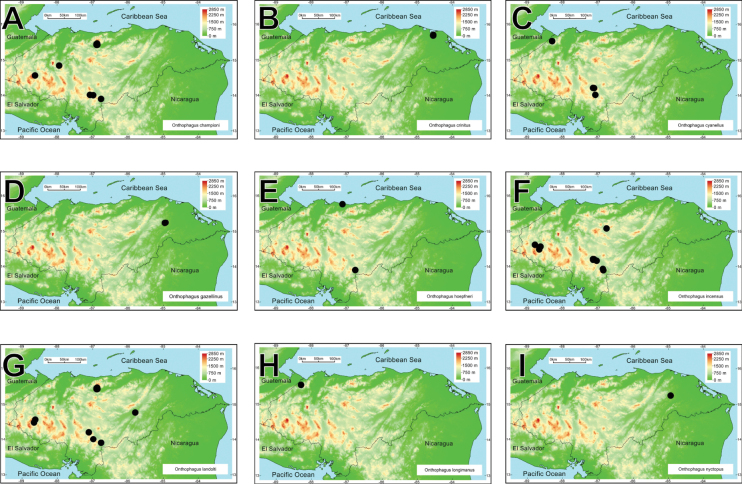
Recorded localities of A. *Onthophagus championi*; B. *Onthophagus crinitus*; C. *Onthophagus cyanellus*; D. *Onthophagus gazellinus*; E. *Onthophagus hoepfneri*; F. *Onthophagus incensus*; G. *Onthophagus landolti*; H. *Onthophagus longimanus*; I. *Onthophagus nyctopus* in Honduras.

**Figure 23.**
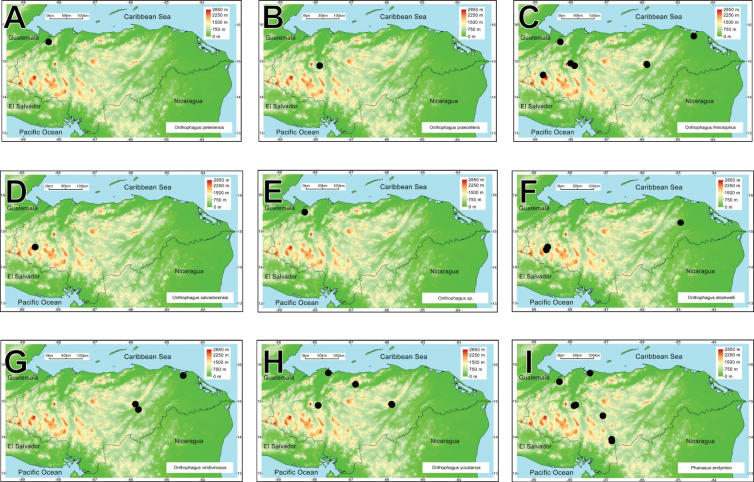
Recorded localities of A. *Onthophagus petenensis*; B. *Onthophagus praecellens*; C. *Onthophagus rhinolophus*; D. *Onthophagus salvadorensis*; E. *Onthophagus* sp.; F. *Onthophagus stockwelli*; G. *Onthophagus viridivinosus*; H. *Onthophagus yucatanus*; I. *Phanaeus endymion* in Honduras.

**Figure 24.**
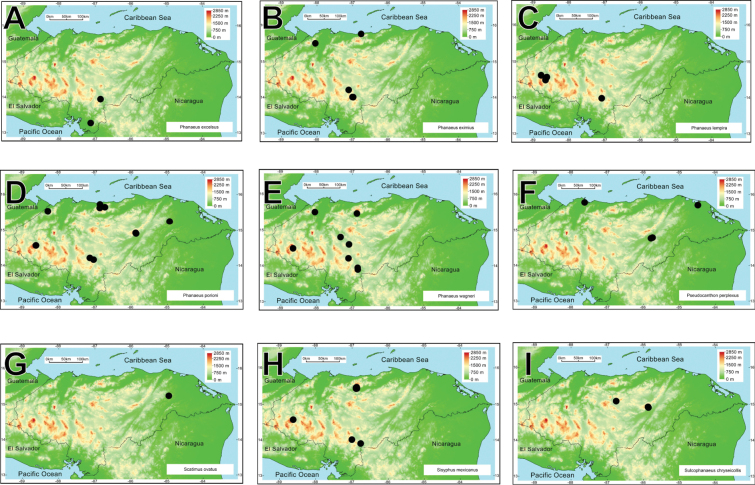
Recorded localities of A. *Phanaeus excelsus*; B. *Phanaeus eximius*; C. *Phanaeus lempira* sp. nov.; D. *Phanaeus porioni*; E. *Phanaeus wagneri*; F. *Pseudocanthon perplexus*; G. *Scatimus ovatus*; H. *Sisyphus mexicanus*; I. *Sulcophanaeus chryseicollis* in Honduras.

**Figure 25.**
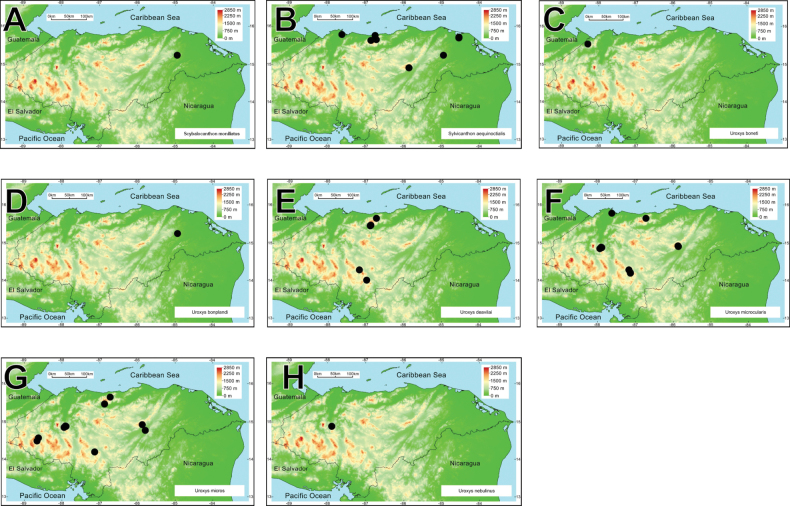
Recorded localities of A. *Scybalocanthon moniliatus*; B. *Sylvicanthon aequinoctialis*; C. *Uroxys boneti*; D. *Uroxys bonplandi*; E. *Uroxys deavilai*; F. *Uroxys microcularis*; G. *Uroxys micros*; H. *Uroxys nebulinus* in Honduras.

## Supplementary Material

XML Treatment for Phanaeus lempira

## ﻿Additional information

### Use of AI

No use of AI was reported.

### Author ORCIDs

José D. Rivera-Duarte https://orcid.org/0000-0001-7610-8446

Renato P. Salomão https://orcid.org/0000-0001-9826-7472

Ángel Solís https://orcid.org/0000-0001-8622-4555

Mauricio Michel https://orcid.org/0009-0009-2331-6810

Marcela I. Matamoros https://orcid.org/0000-0002-2928-206X

Bert Kohlmann https://orcid.org/0000-0002-3441-3933

### Data availability

All of the data that support the findings of this study are available in the main text.
